# Photocyclization of Alkenes and Arenes: Penetrating Through Aromatic Armor with the Help of Excited State Antiaromaticity

**DOI:** 10.3390/chemistry7030079

**Published:** 2025-05-09

**Authors:** Nikolas R. Dos Santos, Judy I. Wu, Igor V. Alabugin

**Affiliations:** 1 Department of Chemistry and Biochemistry, Florida State University, Tallahassee, FL 32306, USA; 2 Department of Chemistry, University of Houston, Houston, TX 77204, USA

**Keywords:** photocyclization, antiaromaticity, arenes

## Abstract

This review focuses on photocyclization reactions involving alkenes and arenes. Photochemistry opens up synthetic opportunities difficult for thermal methods, using light as a versatile tool to convert stable ground-state molecules into their reactive excited counterparts. This difference can be particularly striking for aromatic molecules, which, according to Baird’s rule, transform from highly stable entities into their antiaromatic “evil twins”. We highlight classical reactions, such as the photocyclization of stilbenes, to show how alkenes and aromatic rings can undergo intramolecular cyclizations to form complex structures. When possible, we explain how antiaromaticity develops in excited states and how this can expand synthetic possibilities. The review also examines how factors such as oxidants, substituents, and reaction conditions influence product selectivity, providing useful insights for improving reaction outcomes and demonstrating how photochemical methods can drive the development of new synthetic strategies.

## 1. Introduction

Photochemistry provides a unique platform for accessing chemical transformations that are challenging or unattainable through thermal methods. The beauty of photochemistry is in converting stable functionalities into their reactive “electronic isomers” using light as the only external reagent. Photocyclization reactions have been a cornerstone of synthetic chemistry, serving as powerful tools for constructing complex molecular frameworks. Despite their importance, many reaction pathways remain underexplored, and the mechanisms underlying these transformations are not always fully understood.

In this review, we will concentrate on intramolecular transformations of conjugated π-systems that combine alkene and arene moieties, two of the key organic functional groups.

Both alkenes and arenes undergo photoexcitation via the involvement of π-systems, typically through the promotion of an electron from the highest occupied molecular orbital (HOMO) to the lowest unoccupied molecular orbital (LUMO) upon absorption of ultraviolet (UV) or visible light.

Due to the non-optimal overlap of orbitals in a π-bond, alkenes are high-energy functionalities. For example, ethylene stores ~25 kcal/mol of strain energy [1] while ethyne accumulates ~60 kcal/mol [2] in comparison to their respective alkane reference points. In alkenes, the vertical promotion of an electron to the ethene π* orbital adds ~100 kcal/mol of energy and greatly weakens the C=C bond [3]. Even if some of this energy is quickly dissipated via geometric relaxation at the excited state surface, the overall process creates an even higher “electronic isomer” of the alkene with increased reactivity and different spin and charge distribution [4,5]. Furthermore, excitation that is coupled to a spin flip (intersystem crossing) can convert an alkene into its triplet state with an even more pronounced diradical character and relatively long lifetimes. After absorbing energy and vertical promotion from the ground state, molecules in the newly formed excited states, both singlets and triplets, undergo subsequent significant geometric changes, including twisting and pyramidalization. Twisting can lead to an increased radical character while pyramidalization can also create charge separation [6,7] (“spontaneous polarization”). Twisting is typical for both singlets and triplets, whereas pyramidalization and polarization are more common in singlets.

Unlike alkenes, arenes are protected by symmetry-enforced cyclic delocalization (“aromaticity”). In the ground state, arenes show an aromatic character and are kinetically resistant towards many reactive species. Photochemical excitation offers a conceptually distinct and practical way to activate these stable molecules in a unique “atom-economic” way. Recent advancements in this area have been driven by the development of excited-state aromaticity and antiaromaticity concepts, as described by Baird’s rules [8,9]. Notably, the 4n + 2 aromatic systems, including benzene itself, become antiaromatic and highly reactive in the lowest π, π* states upon excitation. To relieve antiaromaticity, excited state arenes undergo unique geometric transformations [10]. Substituents around an aromatic ring also can take part in relieving the excited-state antiaromatic character. For instance, in the excited state, styrene alleviates antiaromaticity by twisting the exocyclic double bond. A deeper understanding of such structural changes can provide rational guidelines for designing new photochemical transformations.

The goal of this review is to present an overview of known transformations that involve both alkenes and arenes. Such transformations can often pierce though the protective shield of aromaticity and lead to the formation of extended cyclic systems. While we aim to highlight the role of antiaromaticity where relevant, we recognize that theoretical and computational underpinnings of many experimental observations are often lacking. Nonetheless, we believe that summarizing experimental trends provides a foundation for future theoretical studies.

This review is not comprehensive—because the experimental body of work is quite large, we had to select only the representative examples. Furthermore, we intentionally excluded photoredox [11,12] and charge-transfer processes to maintain the focus and manage the scope. These processes, which involve species such as radical ions, differ significantly from the chemistry of excited states [13,14]. By narrowing the focus, we aim to provide a detailed examination of photochemical transformations involving aromatic systems.

### • Escape from Antiaromaticity Converts Benzene into a Highly Reactive Species

We will begin with a short preface discussing the role of aromaticity in the photochemical reactions of aromatics substituted with double and triple bonds. Benzene, the prototypical aromatic molecules in its ground state, exhibits strong antiaromaticity in the S_1_ and T_1_ states. This reversal of electronic properties was derived qualitatively for the lowest triplet state (T_1_) by Baird [8,15], and later extended to the lowest singlet excited state (S1) of benzene through quantum chemical computations [16-20].

The interplay of excited-state aromaticity and antiaromaticity was shown to be important for photophysical and photochemical phenomena [21,22]. The excited-state antiaromaticity of benzene leads to structural and electronic changes that alleviate this destabilization. This escape often leads to dramatic structural distortions, such as the formation of a fused bicyclic structure, which can be trapped by external reagents [10].

**Scheme 1.**
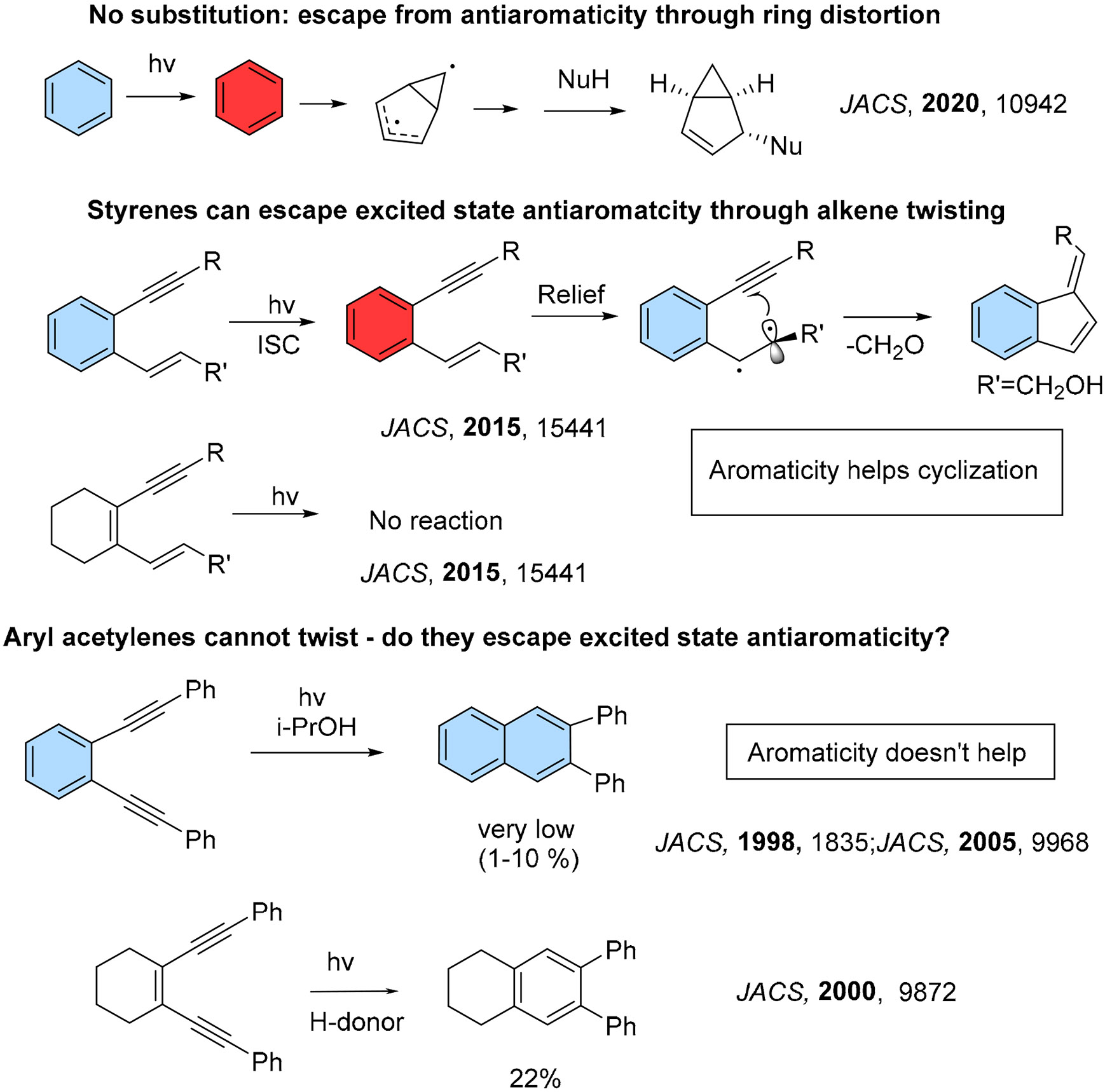
Selected effects of aromatic rings on photochemical reactivity.

However, when an alkene is attached to benzene, alkene twisting relieves antiaromaticity and the arene ring remains intact [23-26]. The twisted alkene is a diradical and can react with a pendant functionality, for example, initiating the C1–C5 cycloaromatization of enynes (Scheme 1) [24].

Alkynes, on the other hand, cannot undergo such twisting, which makes their analogous cycloaromatization highly inefficient (e.g., the photo-Bergman cyclization of a 1,2-diethynyl chromophore) [27-30]. The role of aromaticity is further illustrated by comparing the effects of replacing benzene with cyclohexene in enyne and enediyne reactions (Scheme 1). In an enyne cyclization, replacing benzene with cyclohexene halts the reaction, while in an enediyne cyclization, the opposite effect is observed and the product yield increases. Unfortunately, the influence of excited-state aromaticity on the photo-Bergman cyclization has not been studied. However, for the C1–C5 cycloaromatization, the evolution of antiaromaticity has been analyzed in detail, and the qualitative picture presented in the scheme below is fully supported by computational analyses [24].

#### 1.1. Photochemical Cyclizations Involving Tethered Alkenes and Arenes

Although cyclization reactions can be promoted in many ways, photochemical excitation has several distinct advantages. The use of light as a traceless selective reagent allows one to activate stable functional groups under relatively mild conditions and in a potentially environmentally friendly fashion. Importantly, reactions that are symmetry-forbidden in the ground state may become symmetry-allowed in specific excited states.

**Scheme 2.**
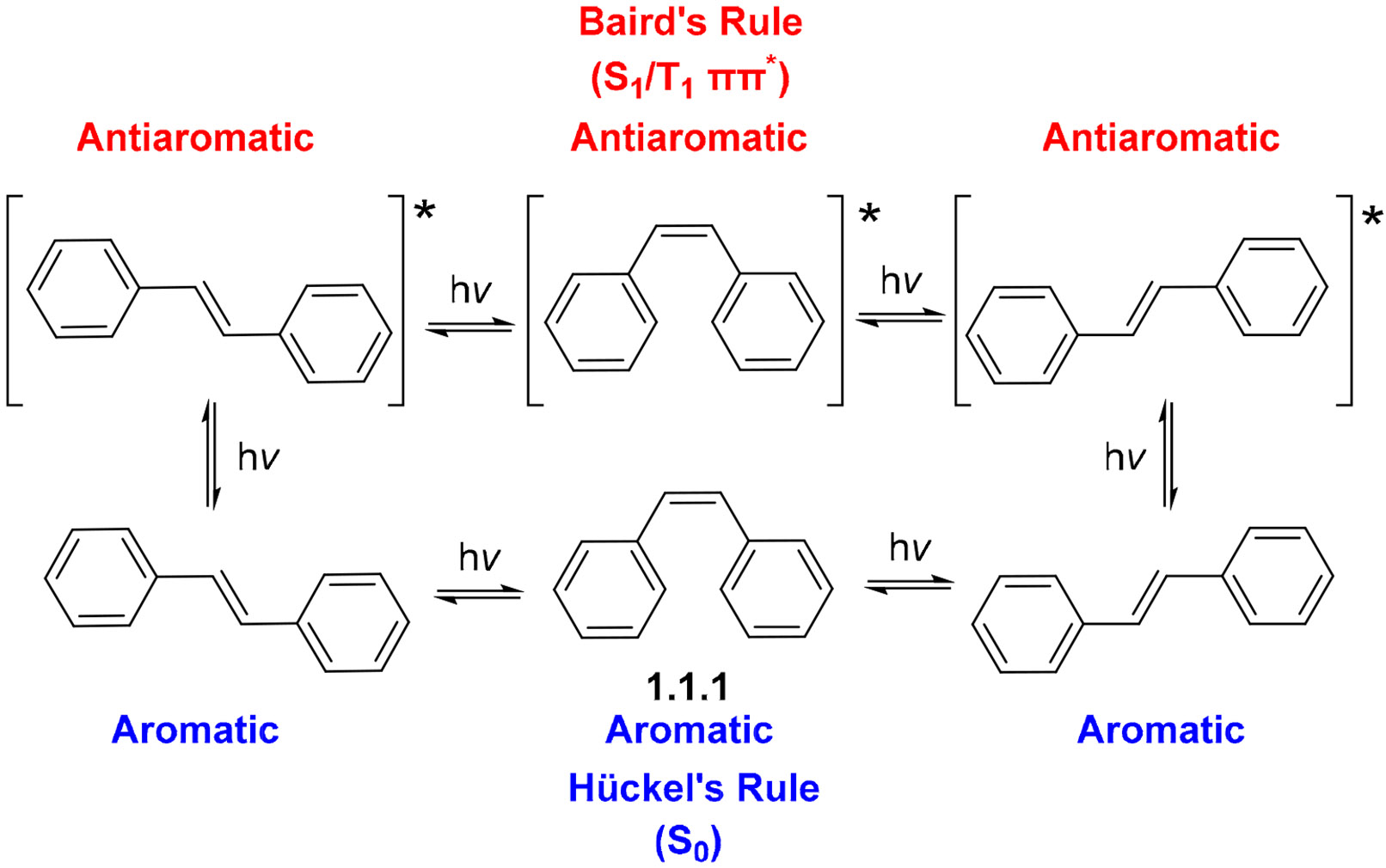
*Cis-trans* isomerization of double bonds in stilbene and summary of Baird’s rules.

Another useful aspect of photochemistry is the ability to interconvert *cis* and *trans* double bonds in alkenes. This isomerization has been extensively studied and shown to occur rapidly under photochemical excitation, rendering *cis* and *trans* double bonds equivalent as synthetic precursors for photocyclization reactions (Scheme 2) [31-33]. Under such conditions, alkenes become stereochemically labile and can adjust to react with their cyclization partner via a transition state geometry that is favorable for the reaction.

As mentioned above, arenes can be converted from aromatic to antiaromatic in photochemical reactions; for example, benzene becomes antiaromatic and highly reactive in the lowest ππ* excited states (Scheme 2). The reversal of the Hückel rules in excited states is known as Baird’s rules [8,9,34,35], a key concept that facilitates the analysis of the photocyclization of aromatic compounds.

The broad goal of this review is to provide a general survey of photochemical reactions where alkenes and arenes react intramolecularly to form a cycle. Specifically, we will focus on the classic photochemical reaction where 1,2-diphenylethene (stilbene) **1.1.1** cyclizes via a 6π-electrocyclization to form dihydrophenanthrene [36]. The resulting dearomatized intermediate has a fleeting existence, and, if not trapped by aromatization, will often undergo an electrocyclic ring-opening back into the stilbene.

**Scheme 3.**
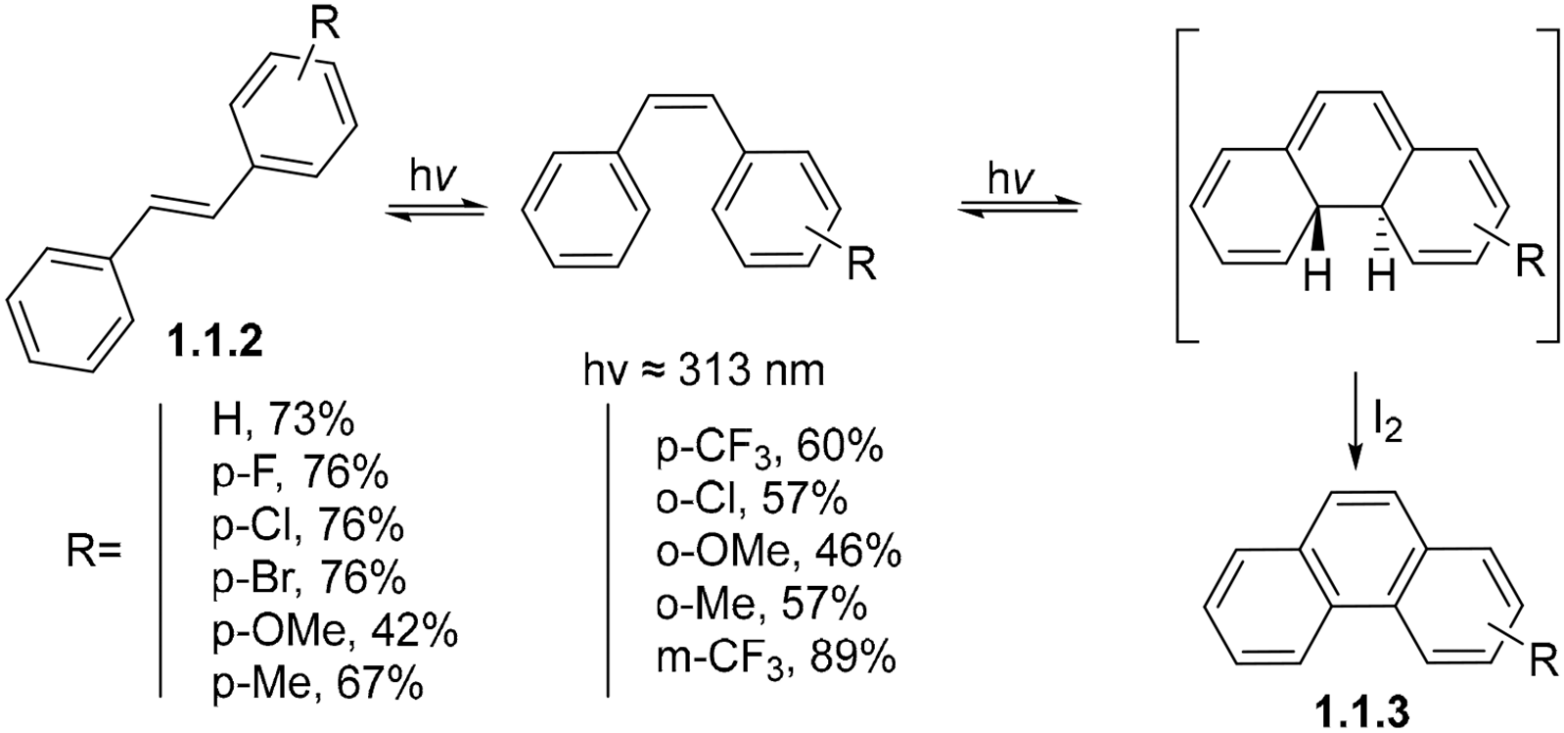
The broad substituent scope of the stilbene photocyclization.

In 1964, Mallory found a way to avoid the ring opening and develop what is now known as the Mallory reaction (Scheme 3) [37,38]. When stilbenes **1.1.2** react in the presence of trace amounts of iodine and O_2_, the dearomatized intermediate is trapped to produce phenanthrene **1.1.3** in good yields.

Upon photoexcitation, I_2_ is homolytically cleaved into radicals responsible for oxidation via radical hydrogen abstractions. The hydrogen iodide formed by this process is then oxidized back to I_2_ by O_2_, replenishing the iodine catalyst. This transformation was compatible with F, Cl, Br, OMe, Me, CF_3_, Ph, and CO_2_H substituents on the benzene ring. On the other hand, *o*-iodo-substituted stilbenes suffered the loss of iodine in the reaction, while stilbenes with nitro, acetyl, or dimethylamino groups did not react.

**Scheme 4.**
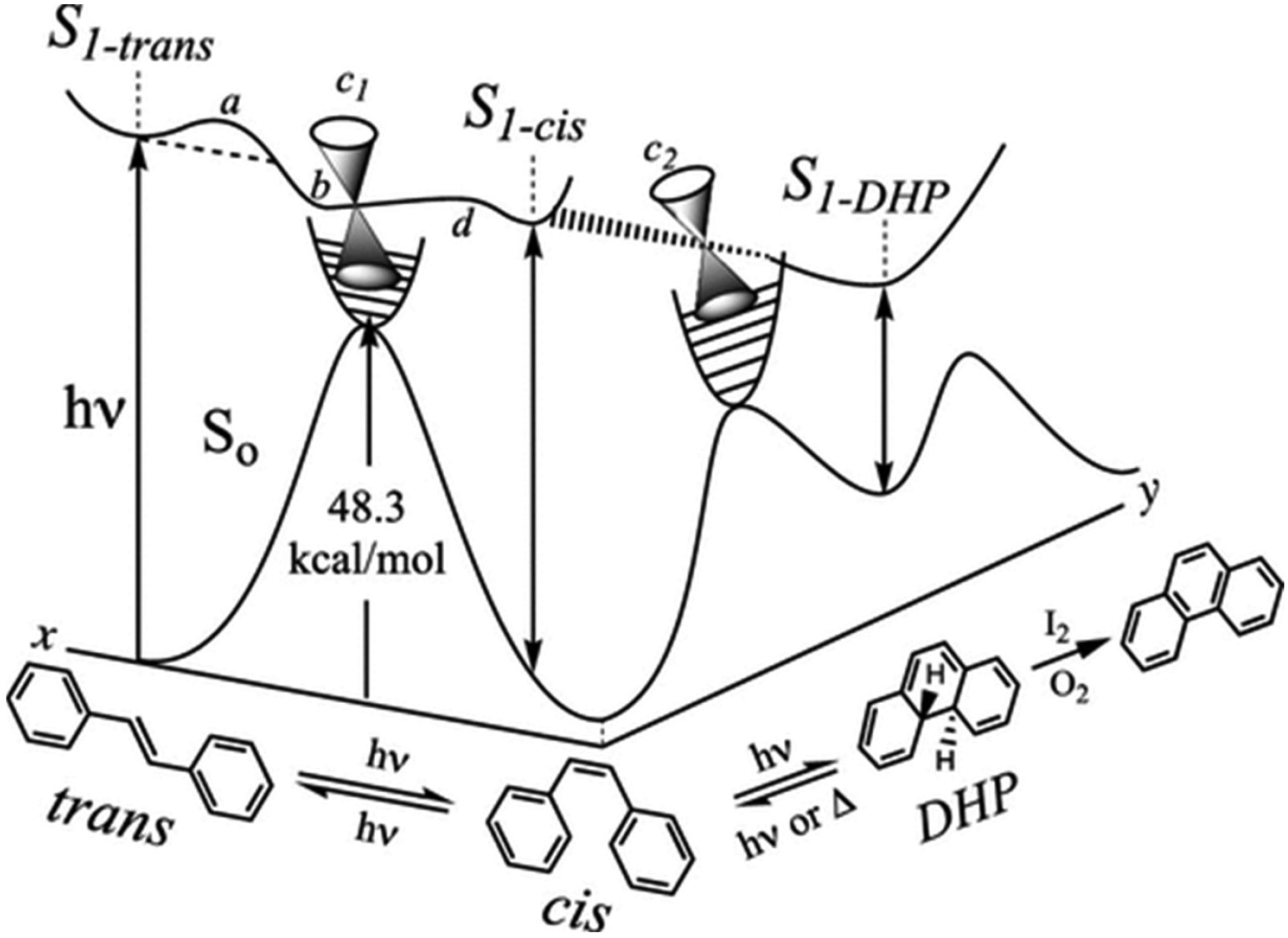
Mechanism and potential energy surface (PES) for Mallory reaction. Copied with permission from [39].

The computed potential energy surface (PES) for the Mallory reaction, derived from the work of Hulley and Clennan [39], illustrates the key mechanistic features of this process for the parent stilbene (Scheme 4). This reaction is relatively insensitive to triplet sensitizers and oxygen, and begins with the excitation of *trans*-stilbene to its first excited singlet state (S1). The excited molecule crosses a small energy barrier (~3 kcal/mol [40]) to reach a twisted intermediate state (also called the phantom state [41], which is close to a conical intersection (c1)) [42]. At this point, it can return to the ground state (S_0_) and form both *trans*- and *cis*-stilbenes in similar amounts.

*cis*-Stilbene can also be excited to S1, where it faces two possible paths: it can either return to the conical intersection (c1) or undergo a conrotatory cyclization to form *trans*-4a,4b-dihydro-phenanthrene (DHP). This cyclization is photochemically allowed and energetically downhill. Once formed, the excited DHP relaxes back to the ground state via another conical intersection (c2).

Alternatively, DHP can form directly from the excited *trans*-stilbene (S1) without leaving the excited state surface, although this is less common. In the final step, DHP is oxidized by either I_2_ or O_2_ to produce phenanthrene.

DHP can also follow competing reactions, such as hydrogen migration to form dihydrophenanthrene isomers or dissociation through cyclo-reversion to regenerate the starting material [43,44]. These multiple branching pathways on the potential energy surface explain the modest quantum yields (0.1–0.5) observed for the Mallory reaction.

Mallory cyclizations were shown to depend strongly on the nature of the chromophore, especially the position of conjugating substituents and the presence of fused aromatic rings. To address that, two reactivity parameters, free valence numbers (F*_r_) and localization energies (L_rs_), have been considered. These parameters are conceptually different since F*_r_ is based on the electronic structure of the excited state (i.e., the reactant in the photocyclization), while L_rs_ is based solely on the structure of the ground state cyclization product. They can be derived from Hückel Molecular Orbital (HMO) theory [45] or higher-level singlet-state wave functions [46,47].

More specifically, F_r_ is defined as F_max_ – F_r_, where F_max_ is the *maximum* possible π-bonding capacity of the atom and F_r_ is the sum of the π-bond orders of all bonds at the atom. This estimates how much bonding potential is still “available” at a given atom. The F*_r_ indexes provide information on which atoms in the molecule become more reactive upon excitation. More specifically, it quantifies the availability of an atomic center to form new bonds in a given electronic state (typically, the first excited state).

Localization energy L_r_ is the energy cost (loss of delocalization stabilization) defined as the decrease in π delocalization energy when the π orbital at atom r is removed from the conjugated system (e.g., to form an s-bond in the photocyclization product). A lower L_r_ means that removing that atom’s electron from delocalization costs little energy. Localization energies can be calculated either for the ground state (L) or for an excited state (L*). Since photocyclizations form a new σ-bond, and hence remove *two* atoms from conjugation, localization energies for evaluating cyclizations have two indexes *r* and *s*, corresponding to the atoms C_r_ and C_s_ connected with the new C–C bond (L_rs_)

The empirical rule for using the localization energy (L*) as a reactivity parameter is that photocyclizations only occur when L* < 3.45β. Even though this approach includes energy difference between two actual molecular species (aromatic precursor and dearomatized cyclic product), the L* values have been used less often.

Laarhoven [45,48,49] has suggested a set of rules for predicting the regioselectivity of such photochemical cyclizations in non-symmetric systems using the F*_r_ indexes. In Mallory photocyclization, for instance, the site with the highest value for the (F*_r_ + F*_s_) sum at the two reacting atoms *r* and *s* indicates the highest reactivity. When multiple reactive positions exist, the value of F*_r_ + F*_s_ helps to predict which site will control selectivity.

**Scheme 5.**
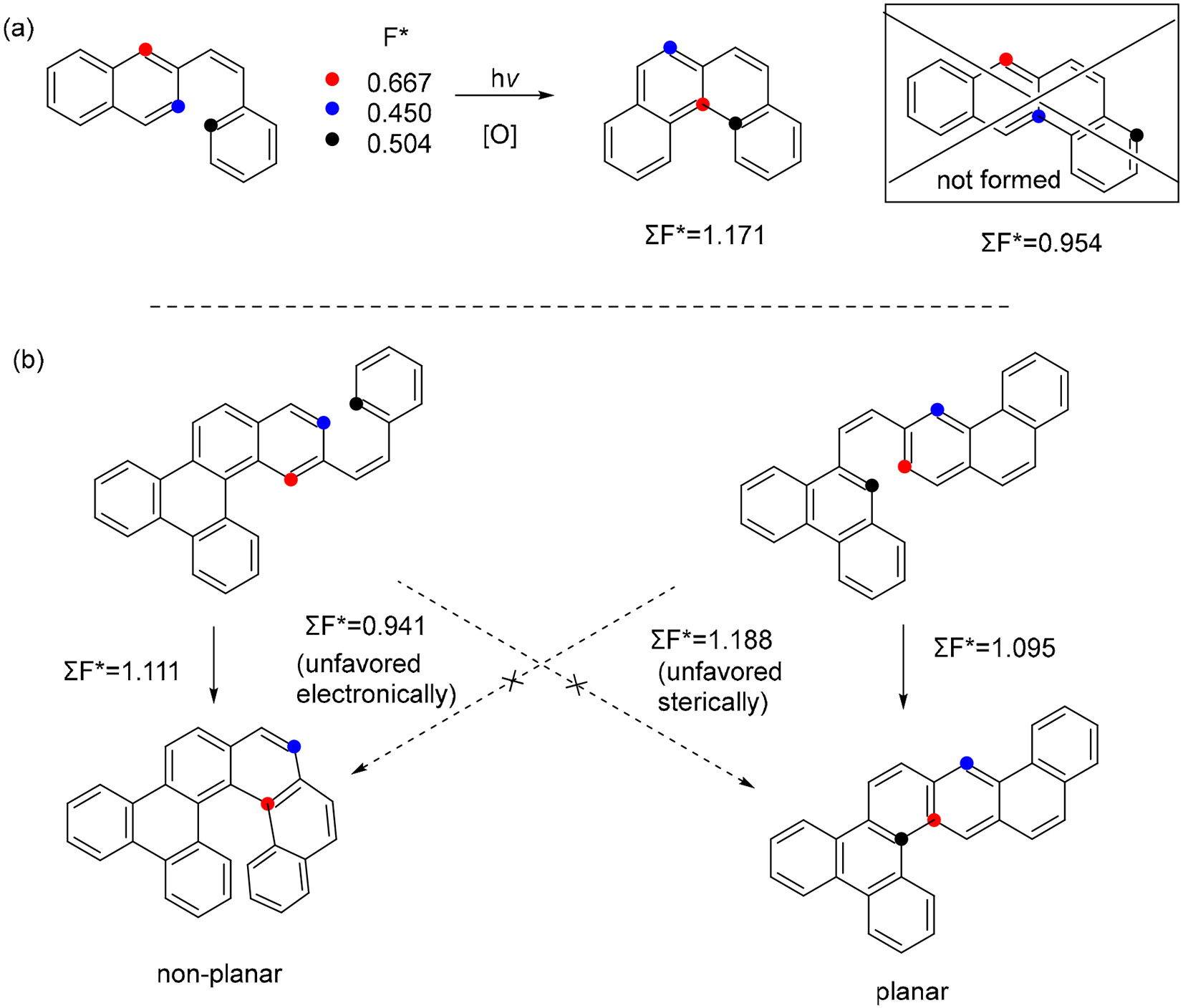
The calculated F* values correlate with the experimental results in Mallory cyclizations. (**a**) Reactivity of naphthyl-substituted styrene. (**b**) Competition of steric and electronic factors in more complex substrates.

The rules can be formulated as follows: (1) photocyclizations do not occur when F*_r_ + F*_s_ < 1.0; (2) one product is formed when the difference in ∑F* between the reacting sites is more than 0.1; and (3) when there is a choice between planar or non-planar products, selectivity favors the planar product. Scheme 5a shows an example of how these calculations can predict the reactivity of the naphthyl-substituted substrate where the reaction can take place at either the α- or the β-positions of the naphthalene ring. The sum of the F*_r_ and F*_s_ indexes is larger for the attack at the α-position. Indeed, the experiment showed that cyclization takes place only there.

Scheme 5b shows a more complex competition wherein a sterically unfavorable helicene formation is made possible by the electronic factors (left). The photocyclization of an isomeric reactant (right) illustrates that if the formation of *both* regioisomers is favorable, the sterically unfavorable non-planar helicene isomer is not formed.

**Scheme 6.**
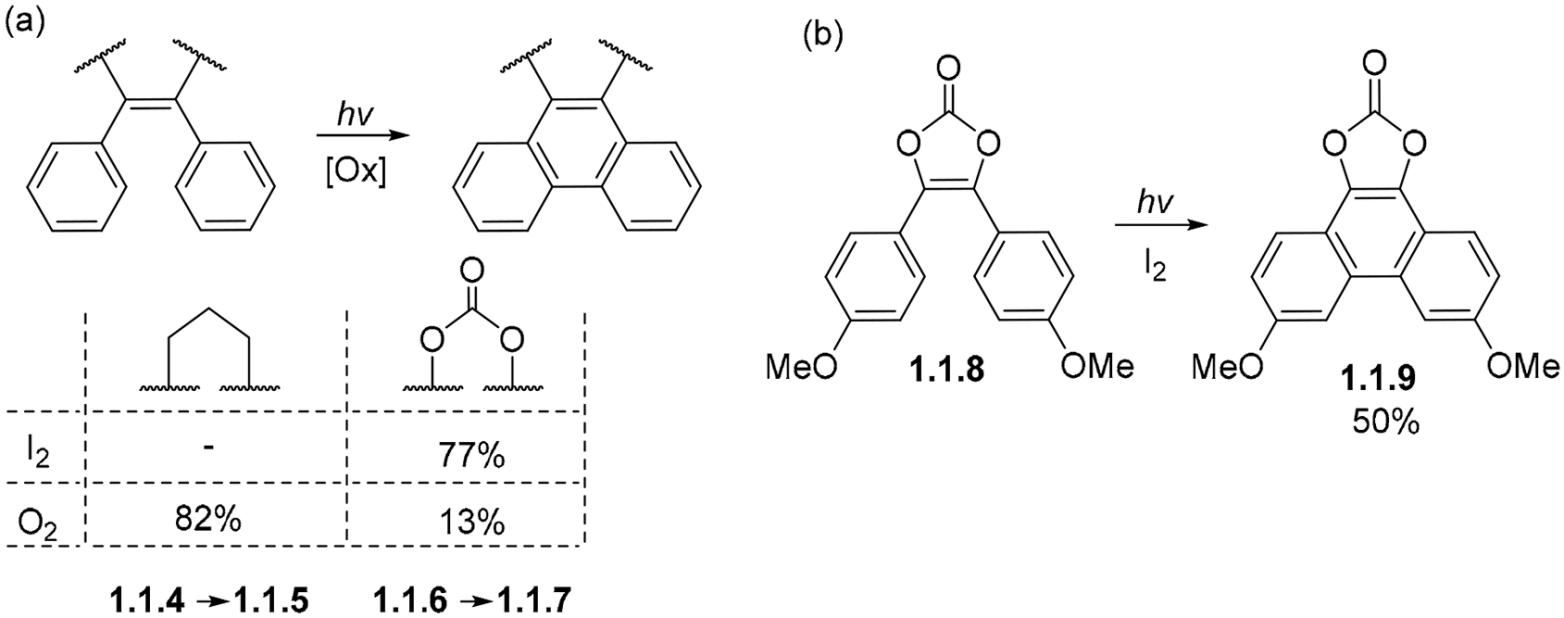
(**a**) Comparison of I_2_ vs. O_2_ as oxidants in photocyclization cascades of strained stilbenes. (**b**) Cyclization of vinylene carbonate under the oxidizing conditions.

Stilbene derivatives containing additional rings may also undergo oxidative photocyclization when these rings do not impose structural constrains that would force the *ortho* carbon atoms too far apart from one another, causing the cyclization to lose in competition with the decay of the excited stilbene to the ground state. 1,2-diphenylcyclopentene **1.1.4** (Scheme 6a) is an example wherein the additional ring does not impose a sufficient strain penalty, thus forming 9,10-cyclopentenophenanthrene **1.1.5** in an 82% yield [38,50].

Another interesting example is the cyclization of vinylene carbonate **1.1.6**, which yields phenanthrene **1.1.7** (Scheme 6a) in a 77% yield with I2 in cyclohexane and a 13% yield with O_2_ in EtOH, while its dimethoxy substituted counterpart **1.1.8** produces **1.1.9** at 50% upon irradiation in the presence of I2 (Scheme 6b) [51].

**Scheme 7.**
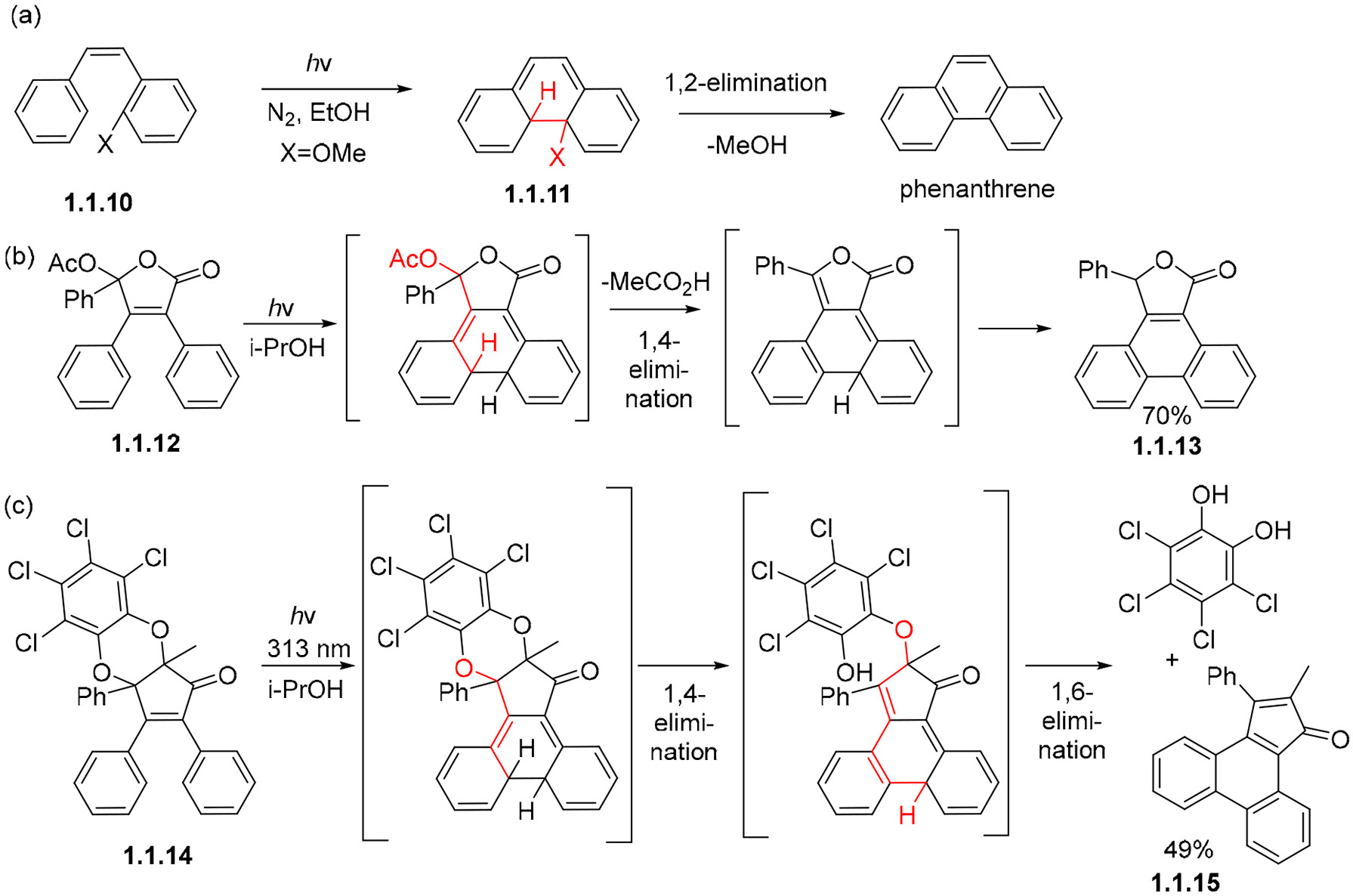
Rearomatizing by trapping the dihydro intermediate via elimination. Atoms and bonds involved in non-oxidative aromatization are shown in red. (**a**) Photocyclization of *o*-OMe-stilbene to phenanthrene. (**b**) Photocyclization of acetoxy lactone via elimination of acetic acid. (**c**) Photocyclization of stilbene derivative via elimination of tetrachlorocatechol.

In the absence of oxidants, the initial cyclization products may also be trapped by elimination if a suitably placed leaving group is present. For example, *o-*OMe-stilbenes **1.1.10** give exclusively phenanthrenes **1.1.11** in N_2_ atmosphere via the loss of MeOH (Scheme 7a) [52]. These leaving groups do not necessarily need to be placed in the ortho position of the stilbene, allowing creativity to play a role in the synthesis of new molecules. As such, the conversion of acetoxy lactone **1.1.12** to aromatized cyclic product **1.1.13** involves the 1,4-elimination of acetic acid followed by tautomerization (Scheme 7b) [53]. Another creative use of unusually placed leaving groups is shown in the non-oxidative conversion of stilbene **1.1.14** to phenanthrene **1.1.15,** where 1,4-elimination is followed by 1,6-elimination and tetrachlorocatechol serves as a leaving group twice (Scheme 7c) [54,55].

**Scheme 8.**
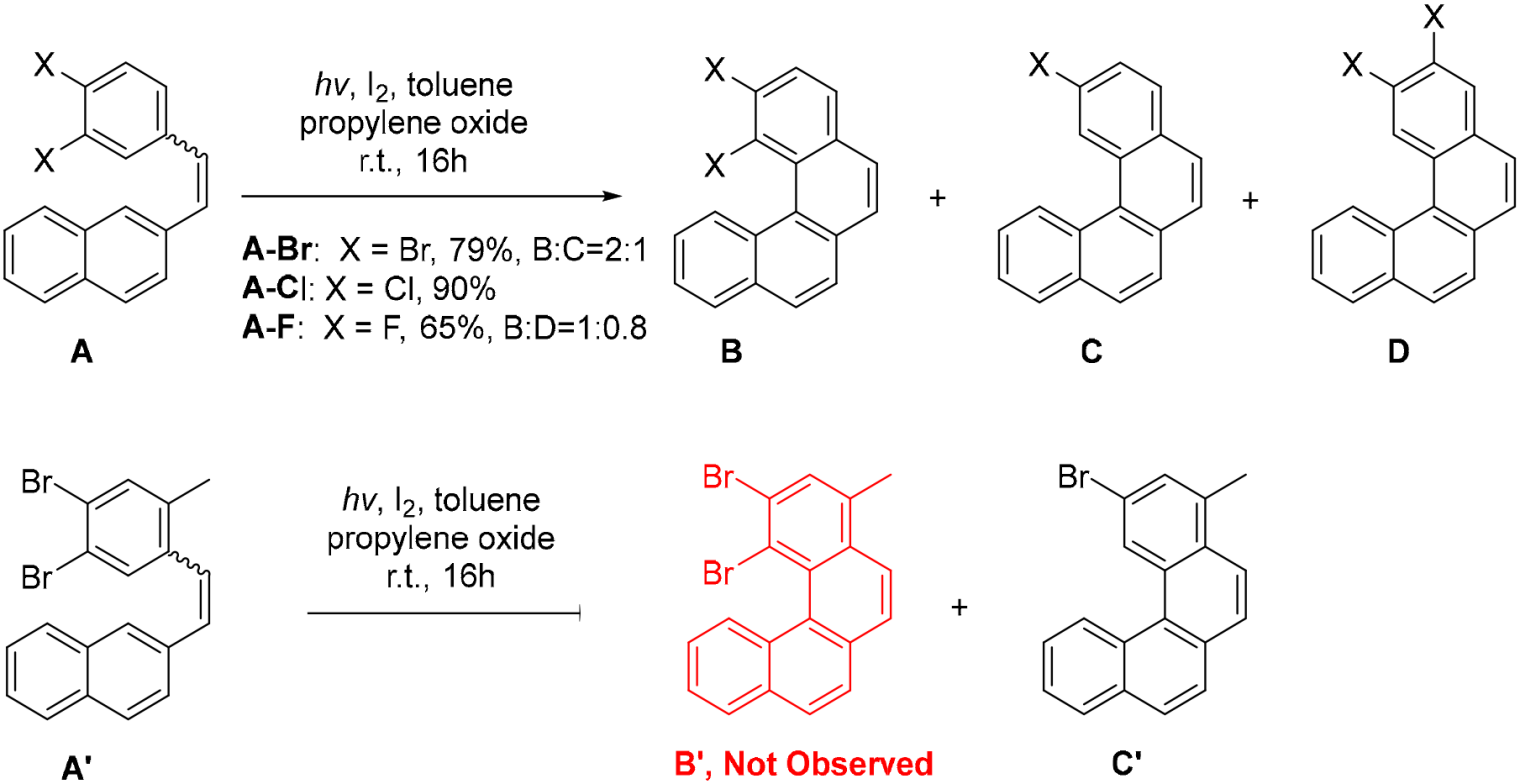
Competition of aromatization mechanisms in the photocyclization of 3,4-dihalostyrylnaphthalenes.

The effects of *meta*- and *para*-halogen-substituted stilbene derivatives were also studied, giving unique results. Avarvari et al. discovered that the photocyclization of 3,4-dihalostyrylnaphthalenes has different selectivity depending on which halogens are present [56]. The Mallory-type photocyclization of **A–Br** yields products **B–Br** and **C–Br** in a 2:1 ratio (Scheme 8). In this instance, the usual Mallory mechanism, where aromatization is achieved by oxidation via with I_2_, is still favored. However, the observed formation of product **C–Br** indicates that aromatization via the release of HBr is possible. On the other hand, the cyclization of **A–Cl** yields the formation of three products, one of which (**D–Cl**) did not have an analog in the reaction of the brominated stilbene **A–Br**. Due to the complexity of the NMR and similar polarities, it was difficult to determine the product ratios for this reaction. Surprisingly, the photocyclization of **A–F** leads to the formation of **B–F** and **D–F** in a ratio of 1:0.8. Based on these results, while the brominated stilbenes give preference to a loss of two hydrogen atoms instead of HBr, when X=F, the formation of HF is much more unfavorable and only di-fluorinated products are formed as a result of **A–F** photocyclization. Another interesting result was derived when one of the possible photocyclization directions was blocked by an ortho Me group. Such modified substrate **A’** provided a single cyclized product **C’** with the concomitant loss of HBr (Scheme 8).

**Scheme 9.**
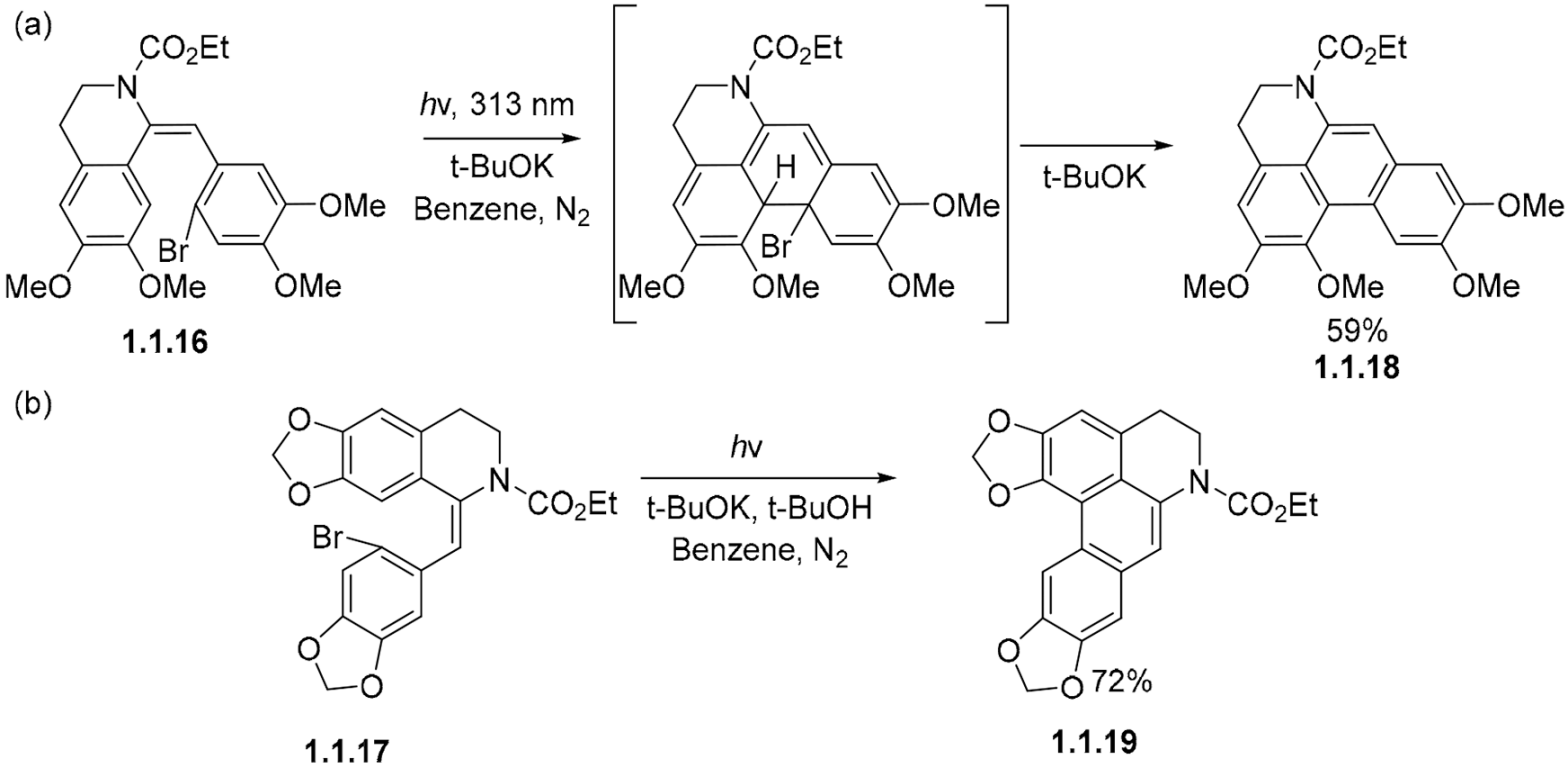
(**a**,**b**) Photocyclizations using base to assist with rearomatization.

A base can also be used to facilitate the trapping of substituted dihydrophenanthrene (DHP) intermediates, as illustrated by the conversions of **1.1.16** (Scheme 9a) and **1.1.17** (Scheme 9b) into **1.1.18** and **1.1.19**, respectively [57]. When bromine is placed in the ortho position of the stilbene bridge, the reaction starts via electrocyclization, and continues by the elimination of the bromine leaving group, resulting in aromatization. This shows an important difference in reactivity as a function of having different halogens as leaving groups. In the next section, the reactivity of *o*-iodostilbenes will be shown to be initiated by the homolytic cleavage of the C–I bond, giving rise to an aryl radical which adds into the opposing benzene ring. This reactivity contrasts the reaction of their bromo analogs, which proceed via the electrocyclization of the stilbene moiety into dihydrophenanthrene followed by the elimination of the halogen leaving group. It is worth noting that the reactivity of Cl-substituted substrates is similar to that of the shown Br-substituted compounds, as seen in the work of Morin et al. [58].

**Scheme 10.**
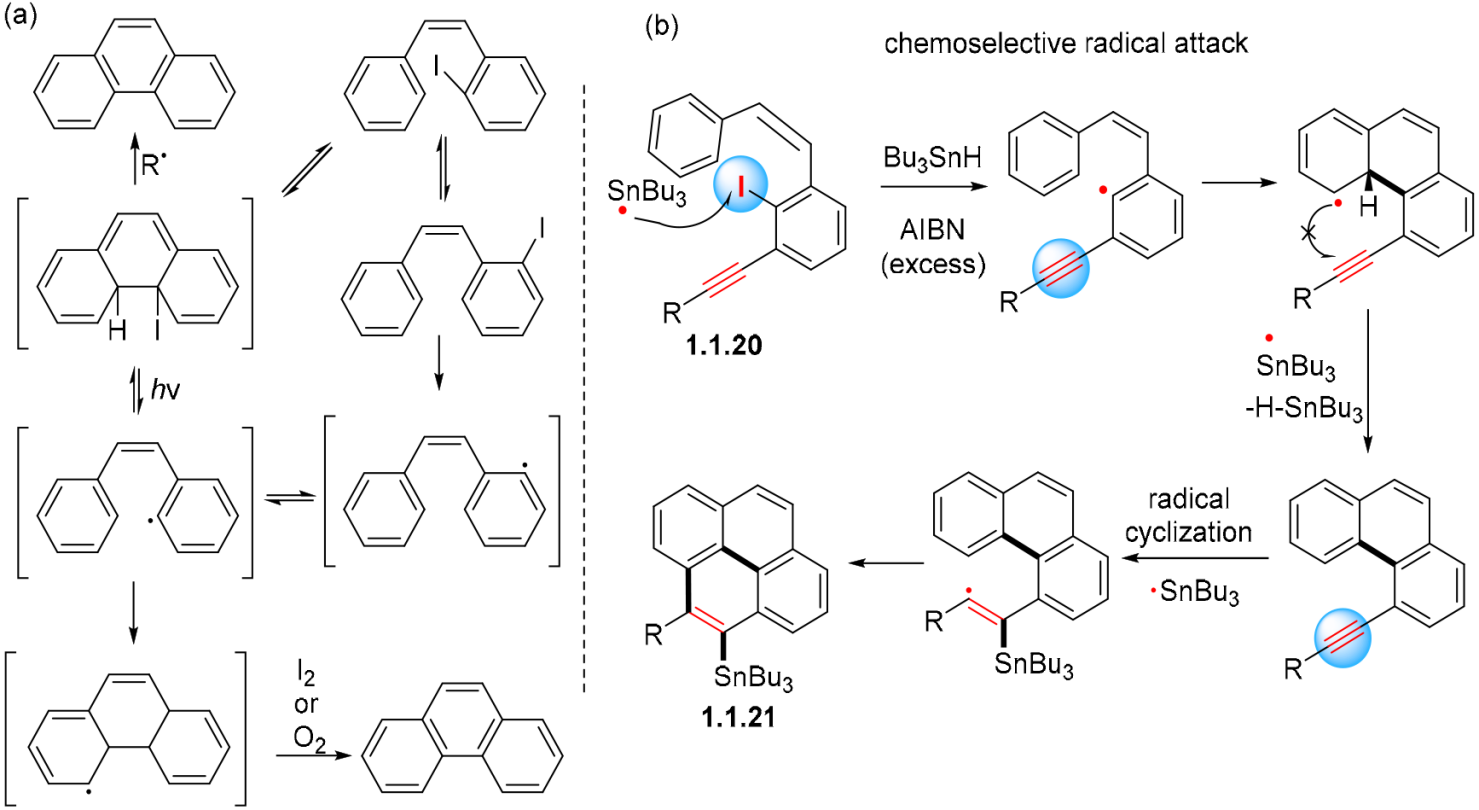
(**a**) Free radical arylations in stilbene photocyclization. (**b**) Example of analogous free radical arylation.

We have mentioned previously that iodo-substituted phenanthrenes can form phenanthrenes by the loss of iodine. It was suggested that the C–I bonds are cleaved photolytically, leading to an intramolecular free-radical arylation (Scheme 10a). Such arylations are known in ground state radical reactions where they can indeed serve for the preparation of phenanthrenes [59,60]. Furthermore, the selective activation of the Ar–I bond in the presence of an alkyne can be useful for the initiation of more extended cascades, as illustrated by the transformation of enyne **1.1.20** into pyrene **1.1.21** (Scheme 10b). A photochemical version of this process would provide a green alternative to such transformations.

**Scheme 11.**
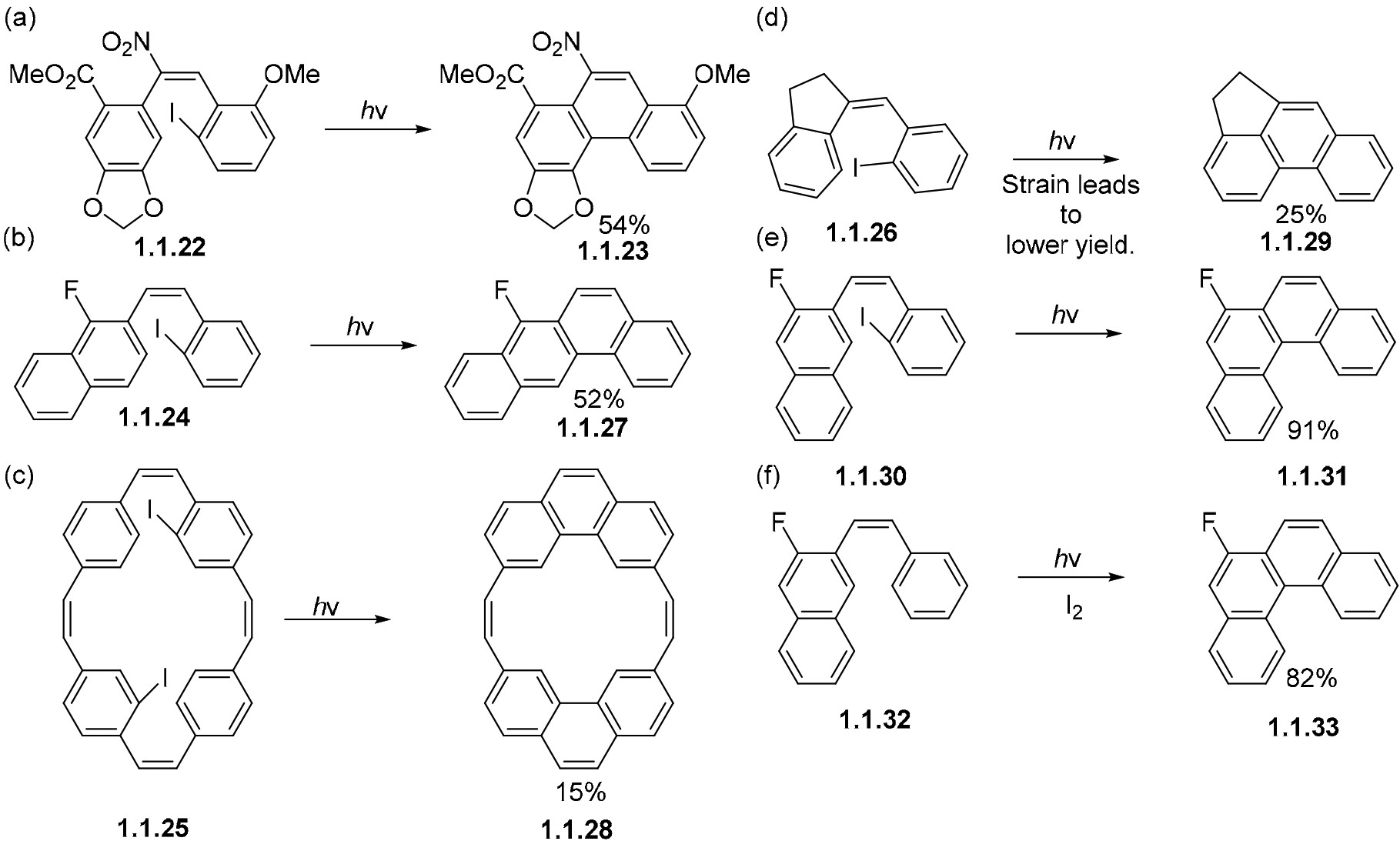
Photocyclizations of *o*-iodo stilbenes. *hv* = 313 nm. (**a**) Photocyclization of *o*-iodonitrostilbene **1.1.22**. (**b**–**e**) Photocyclization of other *o*-iodostilbenes. (**f**) Lack of *o*-iodo substituent in stilbene **1.1.32** requires addition of I_2_ for aromatization to occur.

Additional examples include the transformation of nitrostilbene **1.1.22** to its corresponding phenanthrene **1.1.23** (Scheme 11a) [61], which does not occur through electrocyclization. This puzzling behavior has been well recorded in multiple nitro stilbene derivatives [38,61]. Additionally, attempts to cyclize the 3- and 4-iodo-substituted nitrostilbenes did not afford any phenanthrene products. Non-oxidative conversions of *o*-iodo stilbenes **1.1.24**, **1.1.25**, and **1.1.26** to phenanthrenes **1.1.27** [62], **1.1.28** [63], and **1.1.29** [64] (Scheme 11b-d) are also possible. The same transformations fail under oxidative conditions. Interestingly, the introduction of *o*-iodo substituents sometimes offers a better alternative to oxidative cyclizations, as shown in the comparable transformations of **1.1.30** and **1.1.31** to **1.1.32** and **1.1.33,** respectively (Scheme 11e,f) [65].

**Scheme 12.**
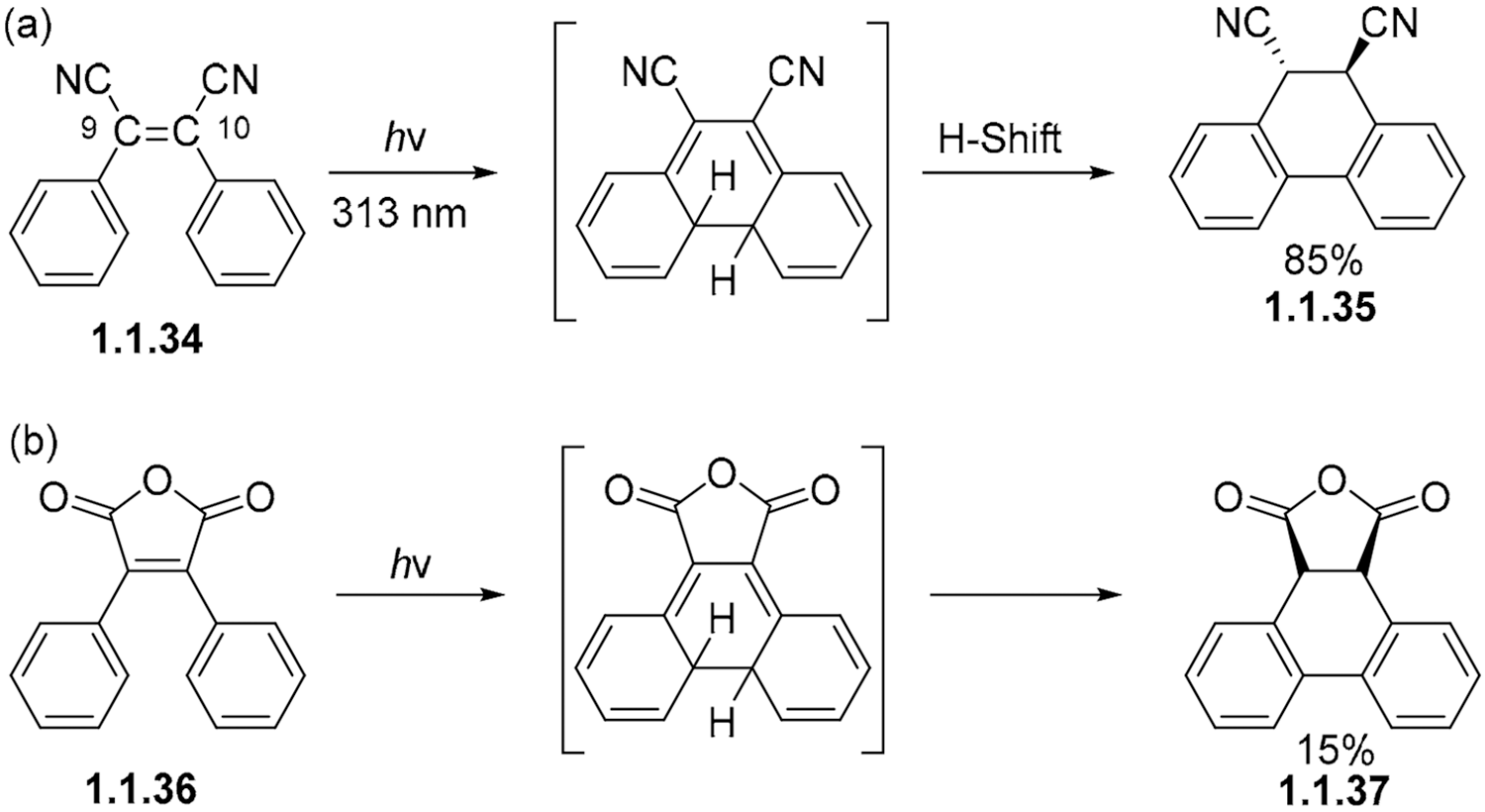
The photocyclization of stilbenes with electron-withdrawing substituents can lead to partial aromatization via 1,3-hydrogen shift. (**a**) Photocyclization of cyano substituted stilbene. (**b**) Photocyclization of diphenylmaleic anhydride.

Since the dihydrophenanthrene intermediate is quite unstable and can open back into the starting stilbene, the non-oxidative photocyclization has to be made irreversible by subsequent steps [37,38]. For example, a series of 1,3-H shifts can give a partially aromatized product with biphenyl moieties incorporated into the tricyclic core [52]. These transformations require suitable groups at the C-9 and/or C-10 positions of the starting stilbene. Such groups include electron-deficient carbonyl or cyano substituents. For example, we can cite the conversion of dinitrile **1.1.34** to *trans*-9,10-dicyano-9,10-dihydrophenanthrene **1.1.35** [66,67] (Scheme 12a) and diphenylmaleic anhydride **1.1.36** to anhydride **1.1.37** (Scheme 12b) [66].

**Scheme 13.**
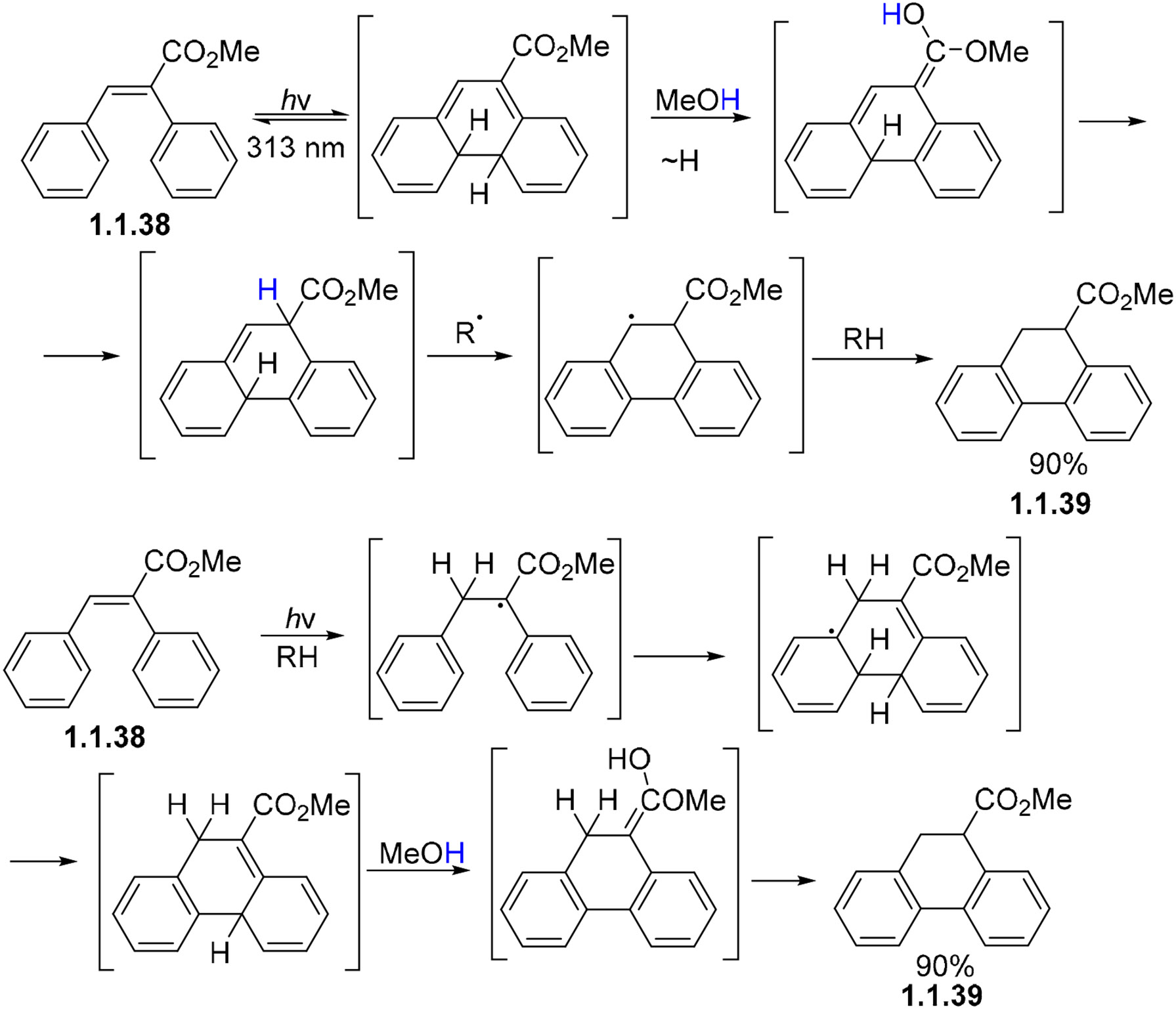
Suggested mechanism of prototropic aromatization after photocyclization in methanol.

On the other hand, the conversion of methyl α-phenylcinnamate **1.1.38** to 9-carbomethoxy-9,10-dihydrophenanthrene **1.1.39** (Scheme 13) follows a different pathway where, after electrocyclization, the resulting intermediate undergoes proton exchange with MeOH, followed by a radical hydrogen abstraction–addition to give the final dihydrophenanthrene product [68]. It is unclear, however, what the source of radical species under anaerobic conditions is.

**Scheme 14.**
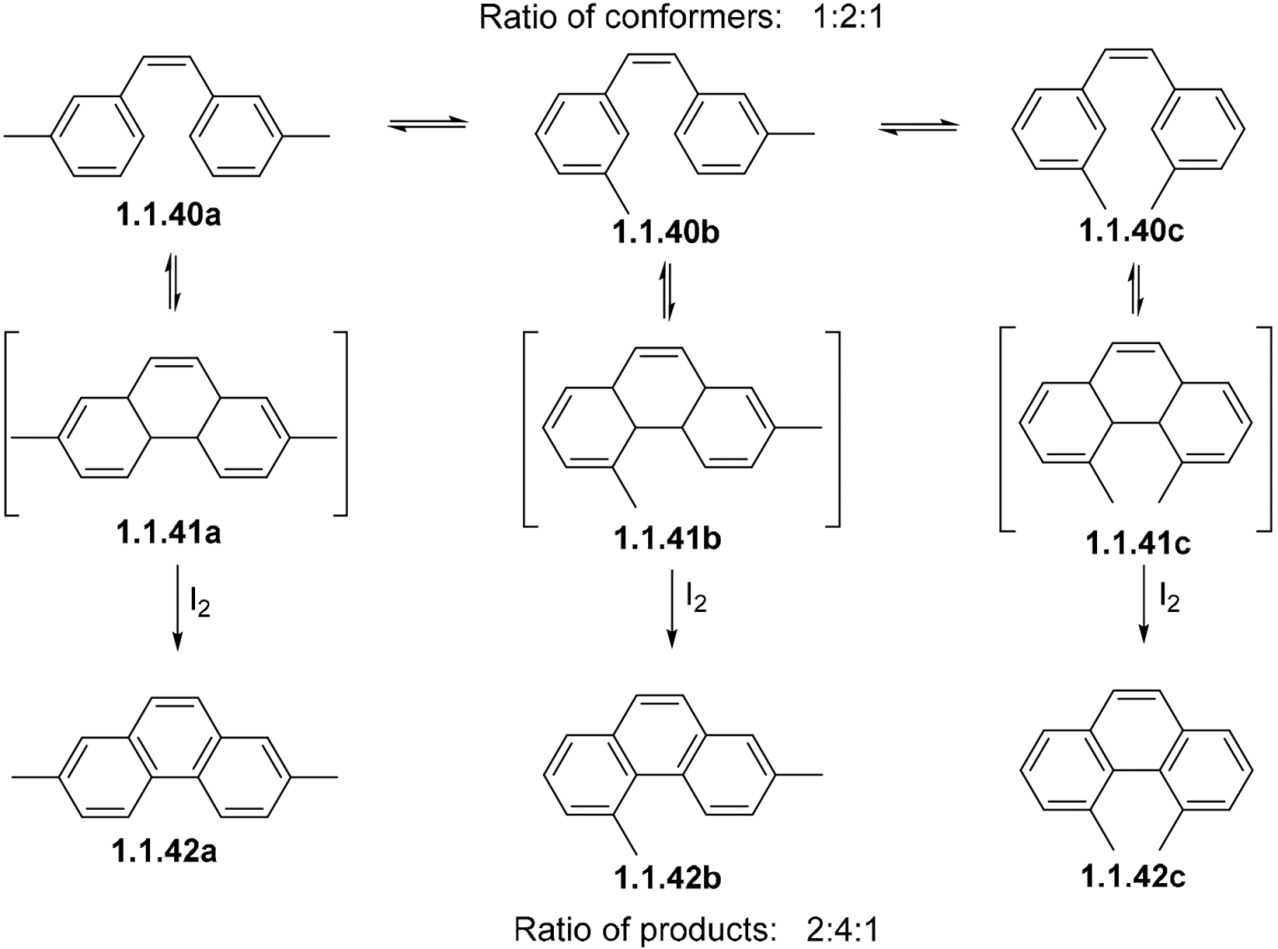
Alkene isomerization in *meta*-substituted stilbenes.

The photocyclization of *meta*-substituted stilbenes can lead to multiple products. In such cases, steric factors can be used as a tool to control the selectivity of cyclization. This effect is shown by the competition of dimethyl stilbene conformers **1.1.40a**, **1.1.40b**, and **1.1.40c** (Scheme 14) in photocyclization. In the presence of I_2_, the oxidative cyclization traps intermediate **1.1.41** to give a 2:4:1 ratio of products **1.1.42a**, **b**, and **c**, respectively. While the 1:2 ratio of products **1.1.42a**:**b** parallels the relative abundance of their precursors **1.1.40a** and **1.1.40b**, Me-Me crowding lowers the yield of **1.1.41c**. Another factor contributing to this selectivity is the greater propensity of **1.1.41c** to undergo ring opening back to **1.1.40c** [69].

**Scheme 15.**
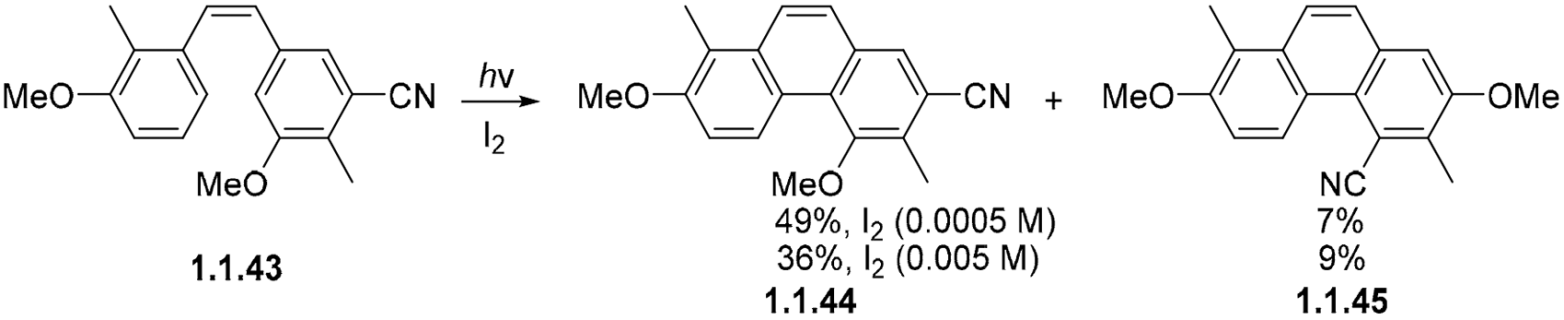
Photocyclizations where oxidant concentration affects selectivity.

Different concentrations of I_2_ can also influence selectivity, as shown in [70,71] in cyanosubstituted **1.1.43**, which gives products **1.1.44** and **1.1.45** in great selectivity (7:1 ratio) with low concentrations of iodine (Scheme 15) [72]. When the I2 concentration is increased to 0.005 M, this selectivity diminishes to 4:1. The iodine concentration affects the kinetics of equilibrium in the mechanism of this reaction. When the iodine concentration is lower, the system has more time to equilibrate between the two possible cyclizations, which gives a higher quantity of the more stable dihydrophenanthrene product.

**Scheme 16.**
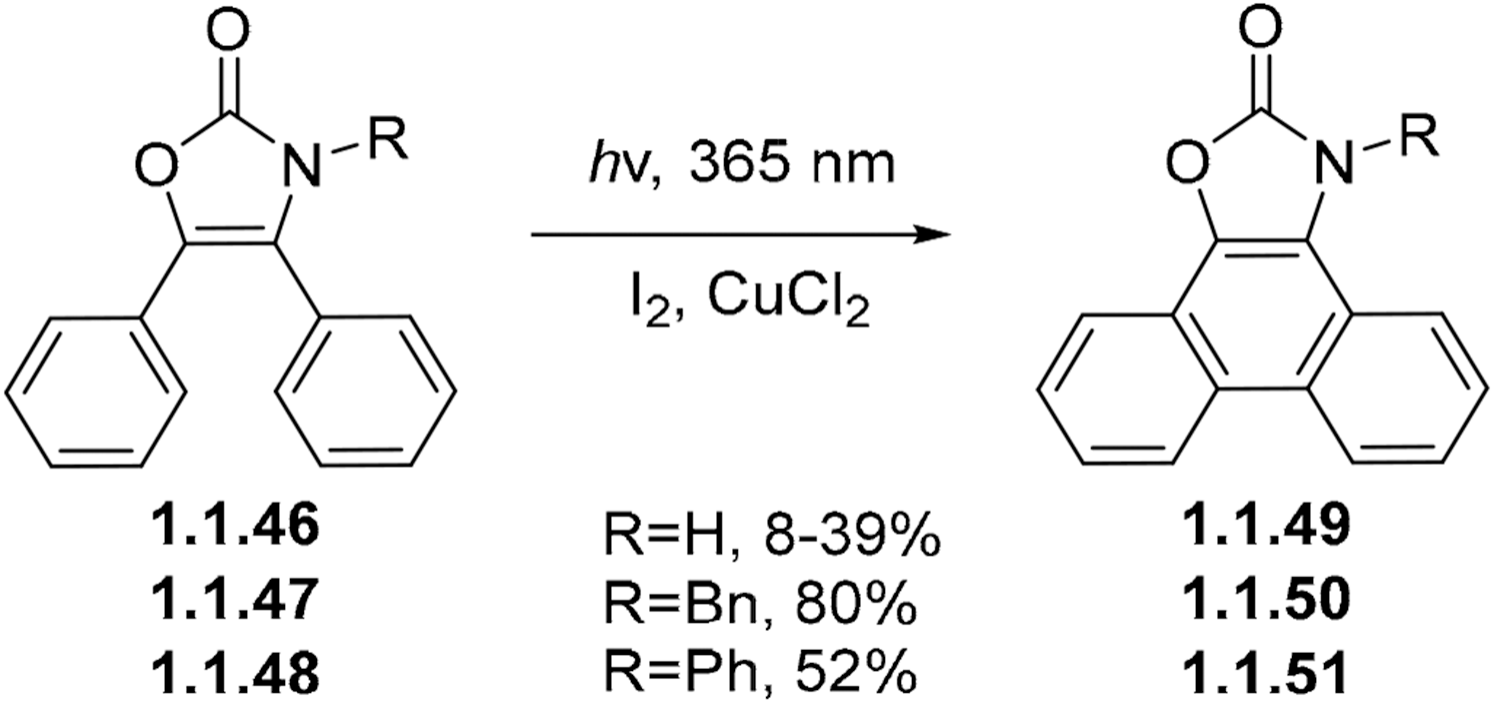
Oxidative photocyclization in the presence of copper chloride.

The role of other redox reagents in Mallory-type photocyclizations has been explored in a few cases. For example, metal salts can be used in addition to I_2_, as illustrated by the oxidative photocyclization of oxazolone **1.1.46**, **1.1.47**, and **1.1.48** (Scheme 16). Here, the cyclization in the presence of I_2_ alone does not afford the desired product. In this instance, only traces of the corresponding phenanthrene product were found. With the addition of CuCl_2_, the reaction proceeds to phenanthro [9,10-d]oxazol-2-ones **1.1.49**, **1.1.50**, and **1.1.51** in 8–39%, 80%, and 52% yields, respectively [73,74].

**Scheme 17.**
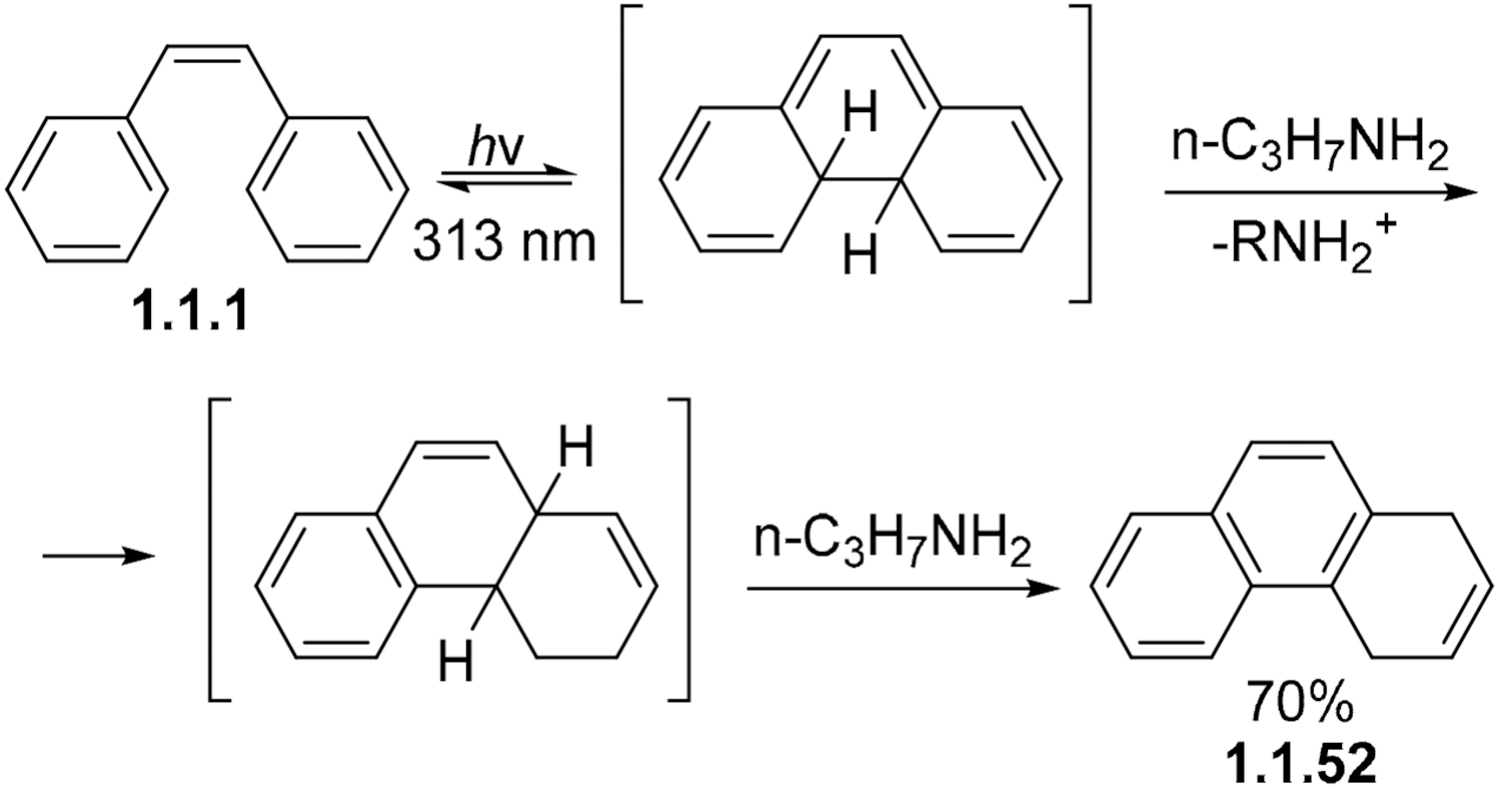
The effect of n-propylamine in the photocyclization of 1,2-diarylethylene leads to unique product formation.

The role of external additives can expand beyond oxidation. For example, non-conjugated dihydrophenanthrenes can also be prepared from *cis*-stilbenes in the presence of *n*-propylamine. This non-oxidative photocyclization of **1.1.1** yields 1,4-dihydrophenanthrene **1.1.52** as a major product (Scheme 17) [75]. The formation of the non-conjugated alkene is interesting, but the mechanism of this process is unclear. This unusual reductive photocyclization seems to be limited to 1,2-diarylethylenes.

#### 1.2. Photocyclizations Involving Extended Polyaromatics

**Scheme 18.**
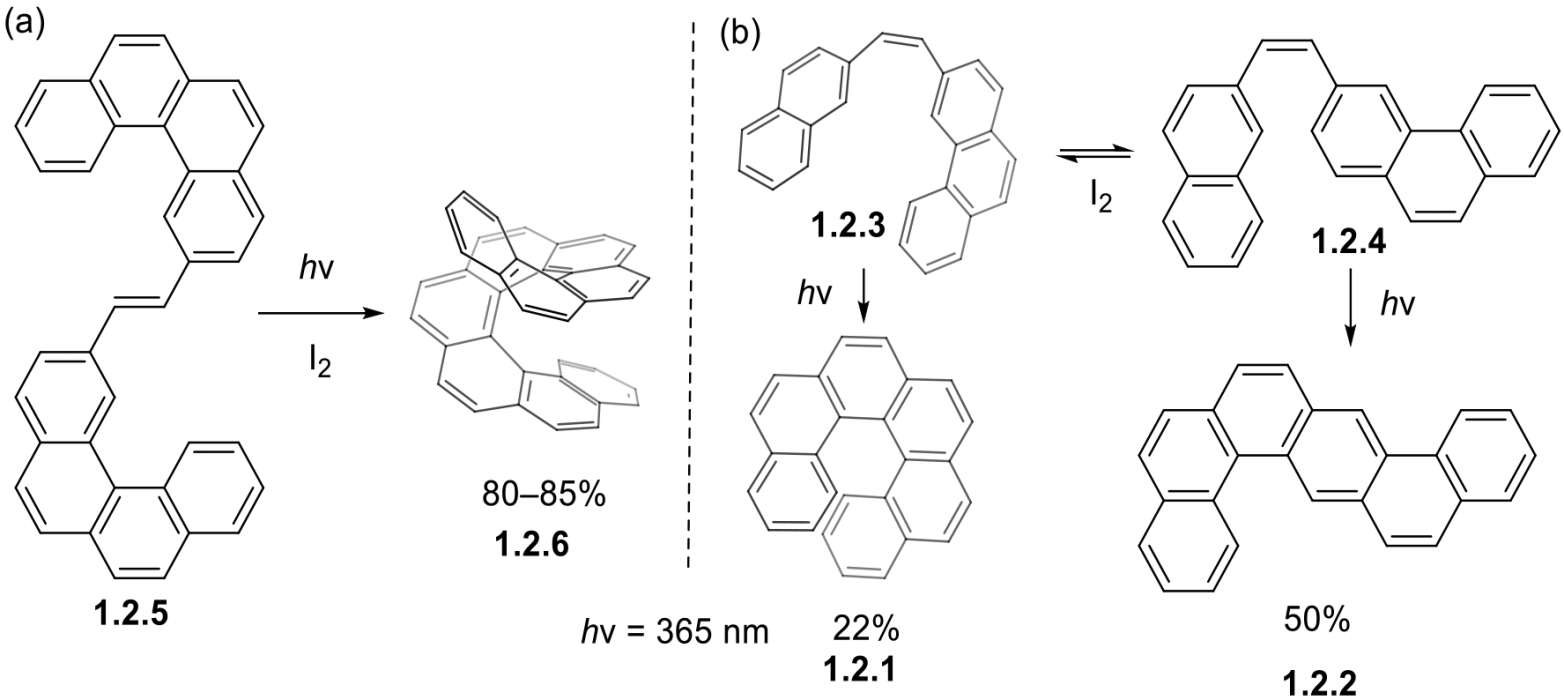
Synthesis of helicenes via oxidative photocyclization of stilbenoids. (**a**) Photocyclization leading to selective formation of [8]helicene. (**b**) Photocyclization of **1.2.3** leads to formation of a mixture of two products.

In photocyclizations that produce extended polyaromatic frameworks, steric constraints may overcome electronic preferences. One example is the competing formation of polyaromatic hydrocarbons (PAH) **1.2.1** and **1.2.2** from conformers **1.2.3** and **1.2.4** (Scheme 18b) [76]. The formation of **1.2.1** creates more steric hindrance between the aromatic rings, which forces distortion while the formation of **1.2.2** does not, leading to its formation in a 50% yield as the major product. However, when both pathways are sterically hindered, electronic preferences dominate, as exemplified by the formation of [8]helicene **1.2.5** from di-3-benzo[c]phenanthrylethylene **1.2.6** in 80–85% yields (Scheme 18a) [77,78].

**Scheme 19.**
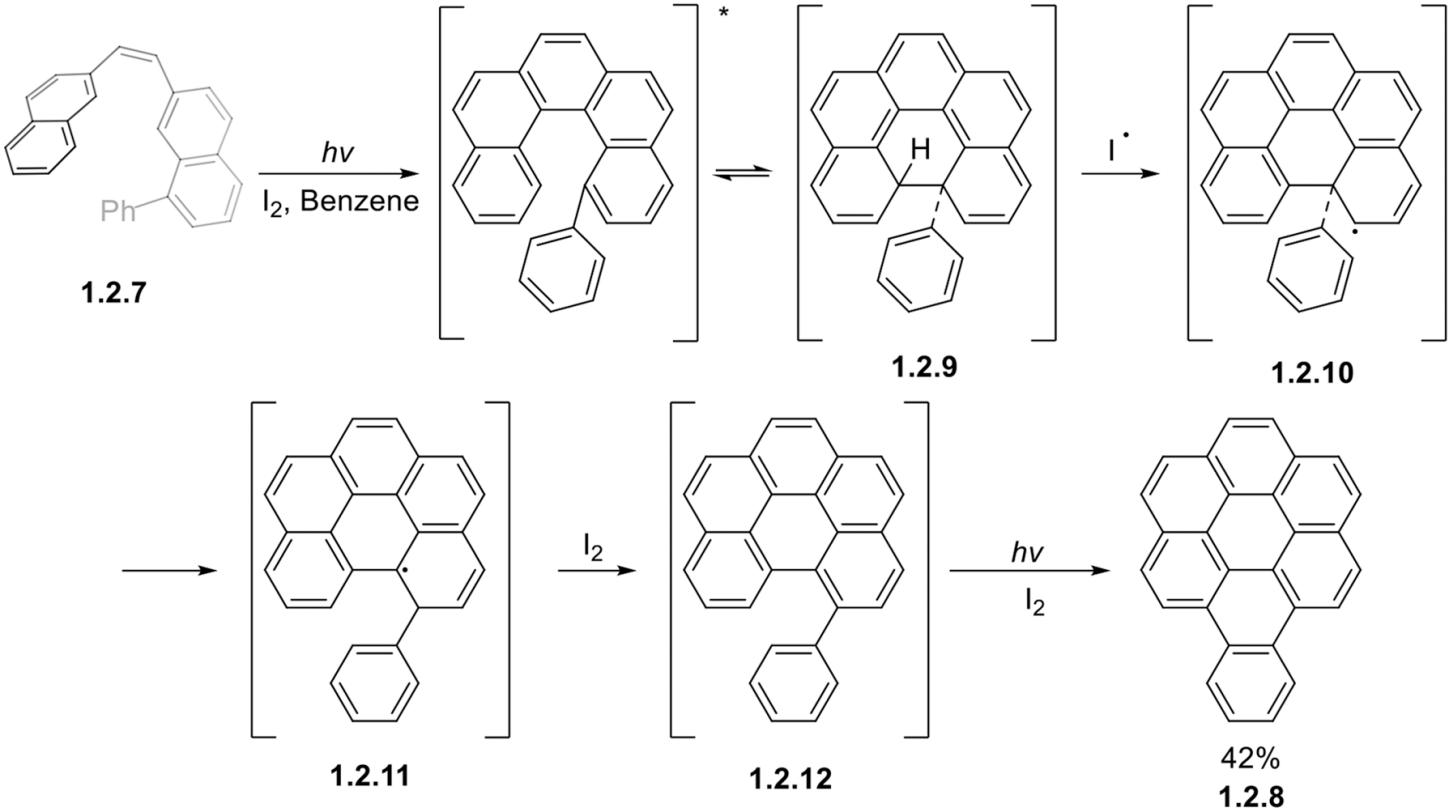
Radical-mediated phenyl migration in oxidative photocyclizations.

In rare cases, photocyclizations may also give rise to the migration of substituents other than phenyl. Generally, this is observed only for the groups attached to the carbon atoms at which bond formation takes place. Such is the case for the conversion of dinaphthyl ethylene **1.2.7** to benzo[*a*]coronene **1.2.8** via dihydro intermediate **1.2.9**. This intermediate is suggested to form a radical **1.2.10** via H-atom abstraction. The migration of a Ph group forms a rearranged radical **1.2.11**, which can aromatize to form **1.2.12**, a suitable starting material for the final cyclization, giving the product **1.2.8** in a 42% yield after in situ oxidation (Scheme 19) [79]. At this point, one also cannot exclude direct cyclization from radical **1.2.11**, followed by iodine-assisted aromatization.

**Scheme 20.**
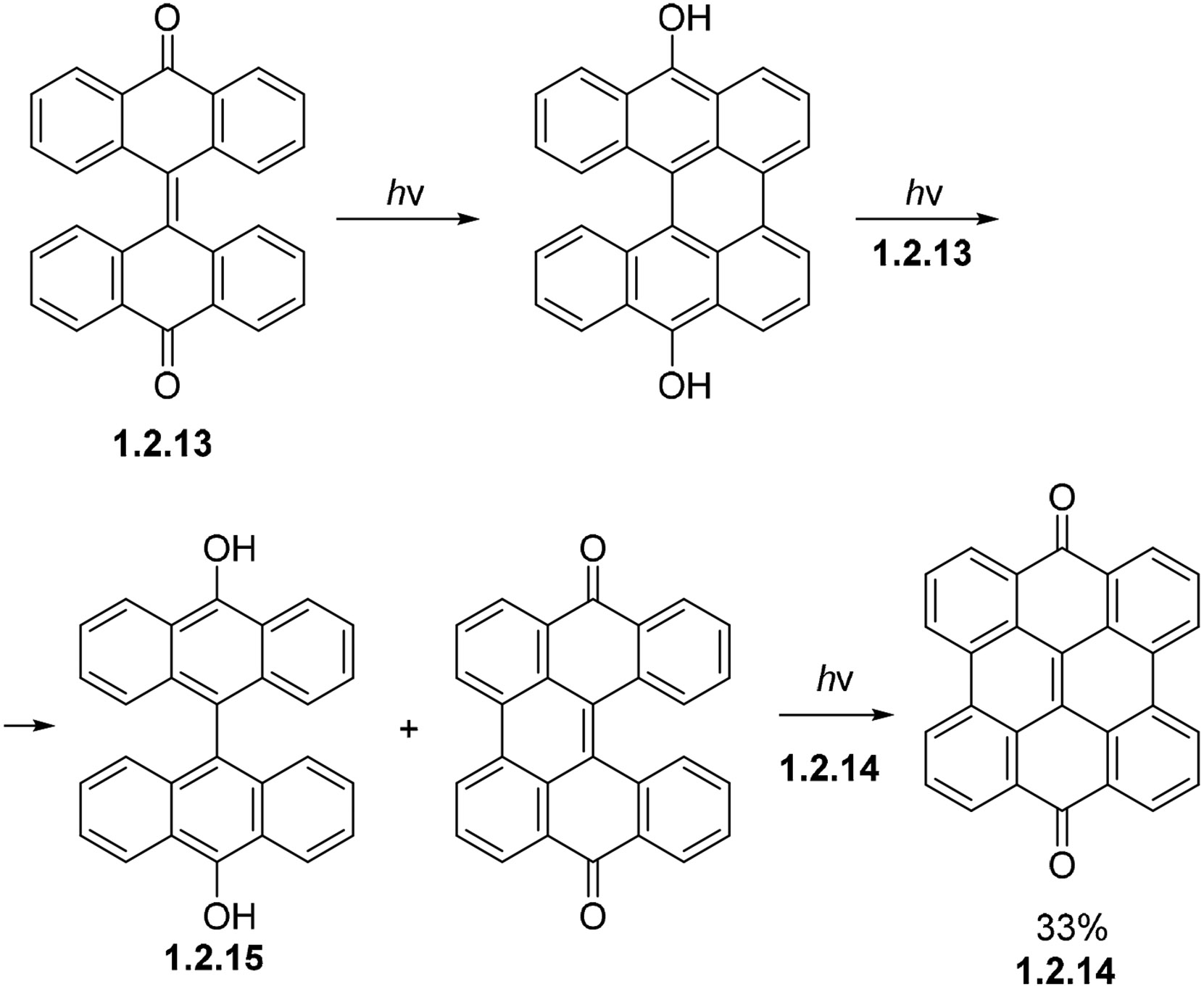
The non-oxidative photocyclization of dianthrone **1.2.14** uses a sacrificial starting material as a hydrogen acceptor for aromatization.

Dianthrones are fully compatible with oxidative photocyclizations. Dianthrone **1.2.13** forms the doubly cyclized product **1.2.14** with a maximum yield of 33%. Here, the intermediates are transformed into the target product by using the starting material **1.2.13** for hydrogen transfers, and dianthranol **1.2.15** is formed as a by-product (Scheme 20). This synthesis, however, can be carried out in almost quantitative yields in the presence of dissolved molecular oxygen [80]. The addition of O_2_ would serve as a radical source sparing the consumption of the starting material.

#### 1.3. Photochemical Ring Closures of Fully Aromatic Precursors

**Scheme 21.**
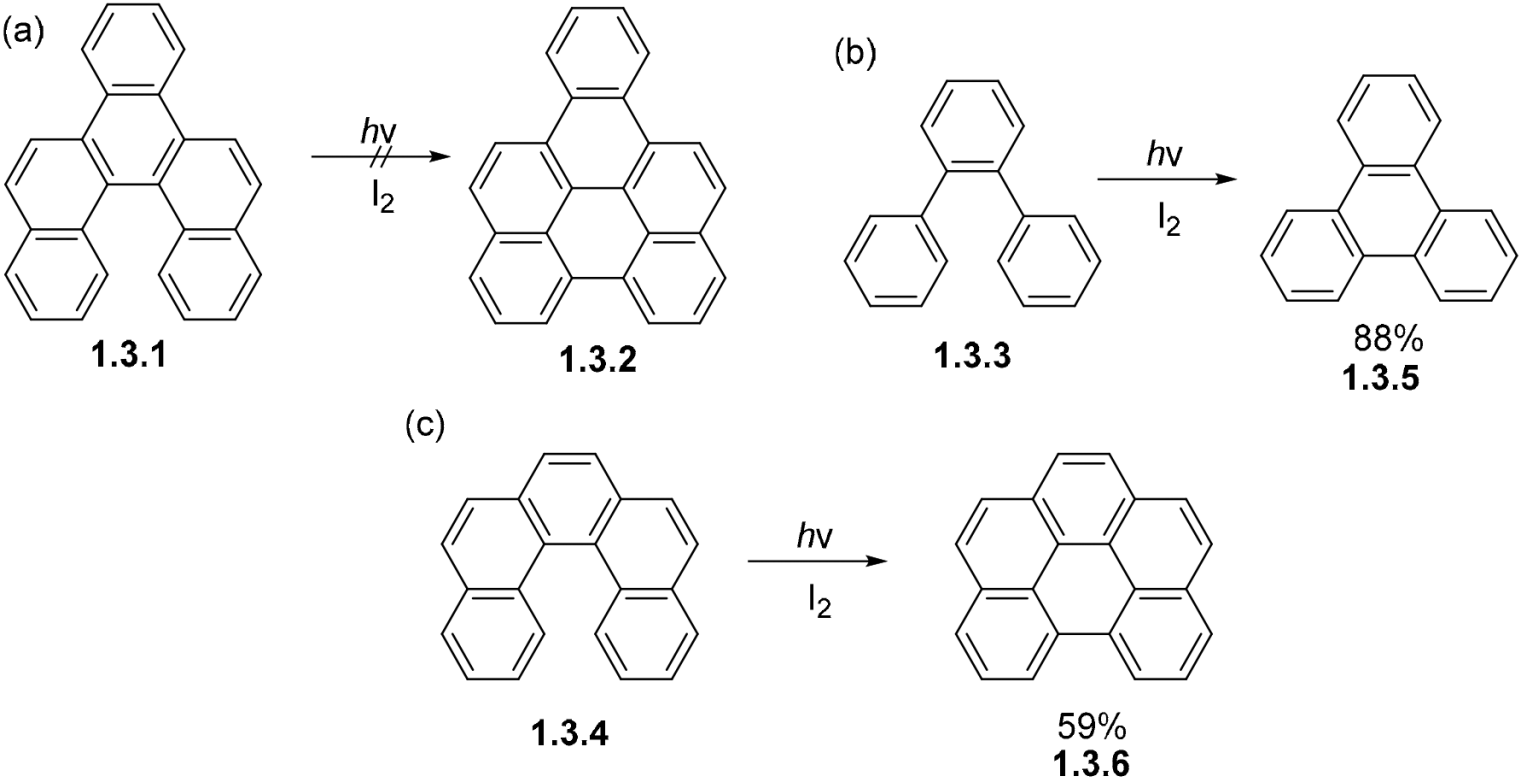
Varied oxidative photocyclization of PAHs show varying reactivity with different annelation patterns- (**a**) the photocyclization fails to proceed for the larger PAH **1.3.1.** but occurs for the smaller PAHs **1.3.3** and **1.3.4** (**b**,**c**).

PAHs with varying annelation patterns show varying photocyclization reactivity. For example, **1.3.1** fails to undergo oxidative photocyclization to **1.3.2**, while **1.3.3** and **1.3.4** readily form the cyclized products **1.3.5** and **1.3.6** in 59% and 88% yields under similar conditions (Scheme 21a-c). Molecular orbital calculations suggest that the coefficients for HOMO and LUMO at the carbons that undergo bond formation become more antibonding after excitation for **1.3.1**, but are more bonding for **1.3.4** (Scheme 21c) [52].

#### 1.4. Single Aromatic Ring and Two Alkenes

**Scheme 22.**
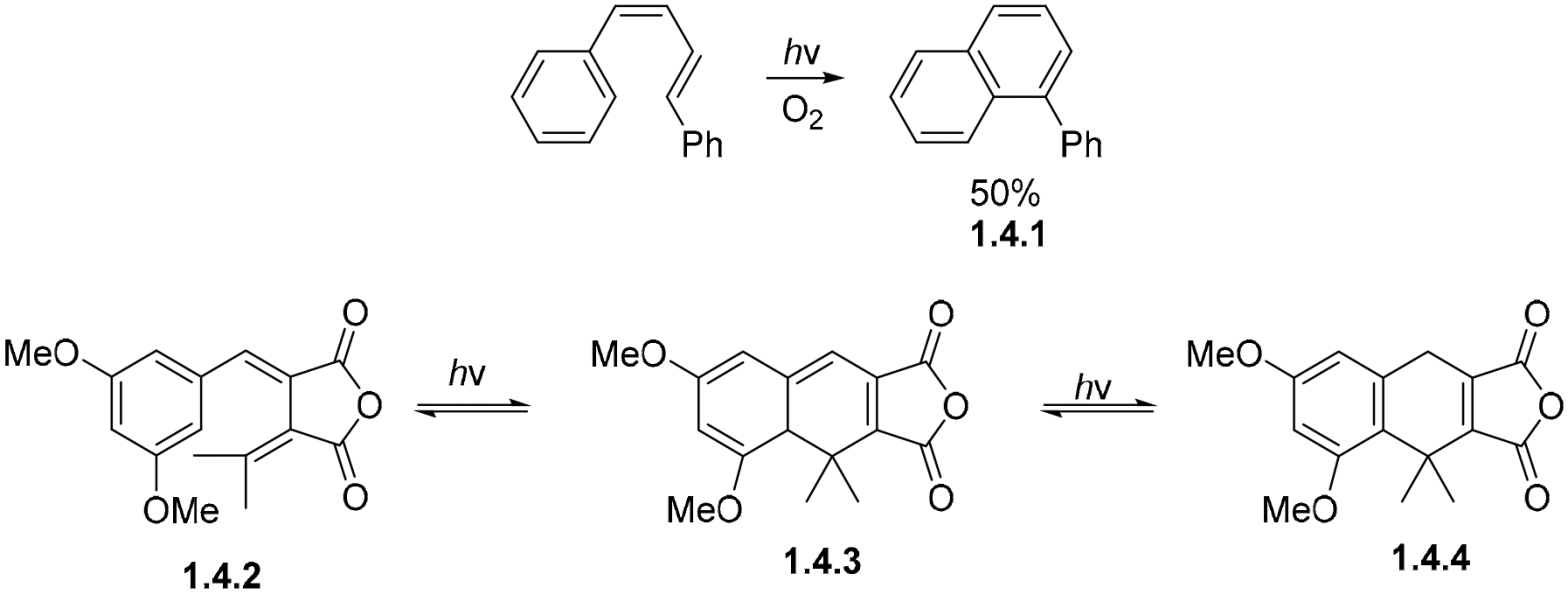
Photocyclizations of 1-aryl-1,3-butadienes.

Dienes with terminal or internal aryl substituents can undergo similar electrocyclizations, as illustrated by the transformation of diphenylbutadiene to phenyl-substituted naphthalene **1.4.1**. The product is formed in a 50% yield under UV irradiation in the presence of oxygen [81] (Scheme 22). This example requires oxygen as an oxidant to form a fully aromatized naphthalene product. Full aromatization is impossible for the cyclization of a 1-aryl diene **1.4.2**. The intermediate 1,8a-dihydronaphthalene **1.4.3** is converted to 1,4-dihydronaphthalene **1.4.4** after prolonged irradiation at 366 nm. Aromatization proceeds via a 1,3-hydrogen shift [82].

**Scheme 23.**
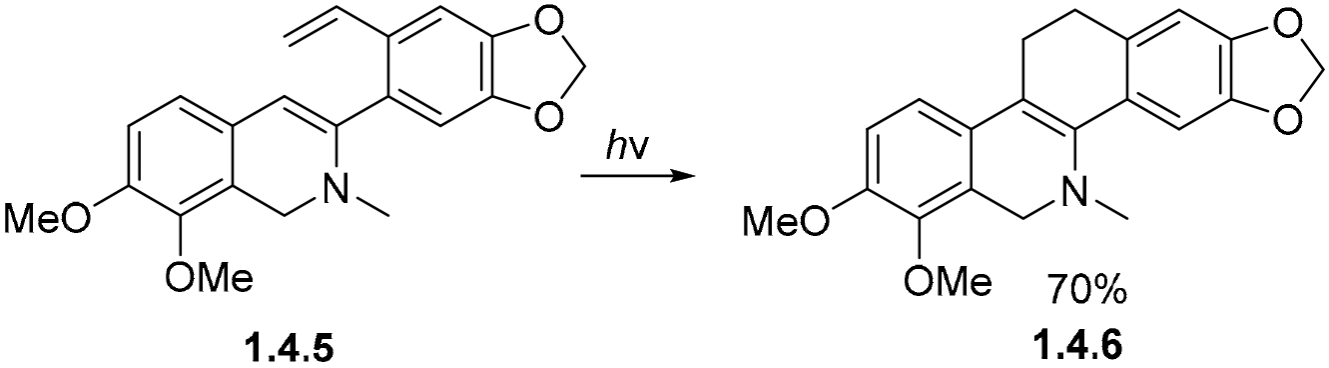
Diene photocyclization proceeds via H-shifts to form a stable product.

On the other hand, the photocyclization of *o*-divinylbenzenes such as **1.4.5** proceeds via the formation of 2,3-dihydronapthalene [83]. Due to the absence of an oxidant, such as O_2_ or I_2_, the reaction is completed via a 1,5-hydrogen shift, returning conjugation to the molecule to yield the tetracyclic product **1.4.6** in a 70% yield (Scheme 23).

**Scheme 24.**
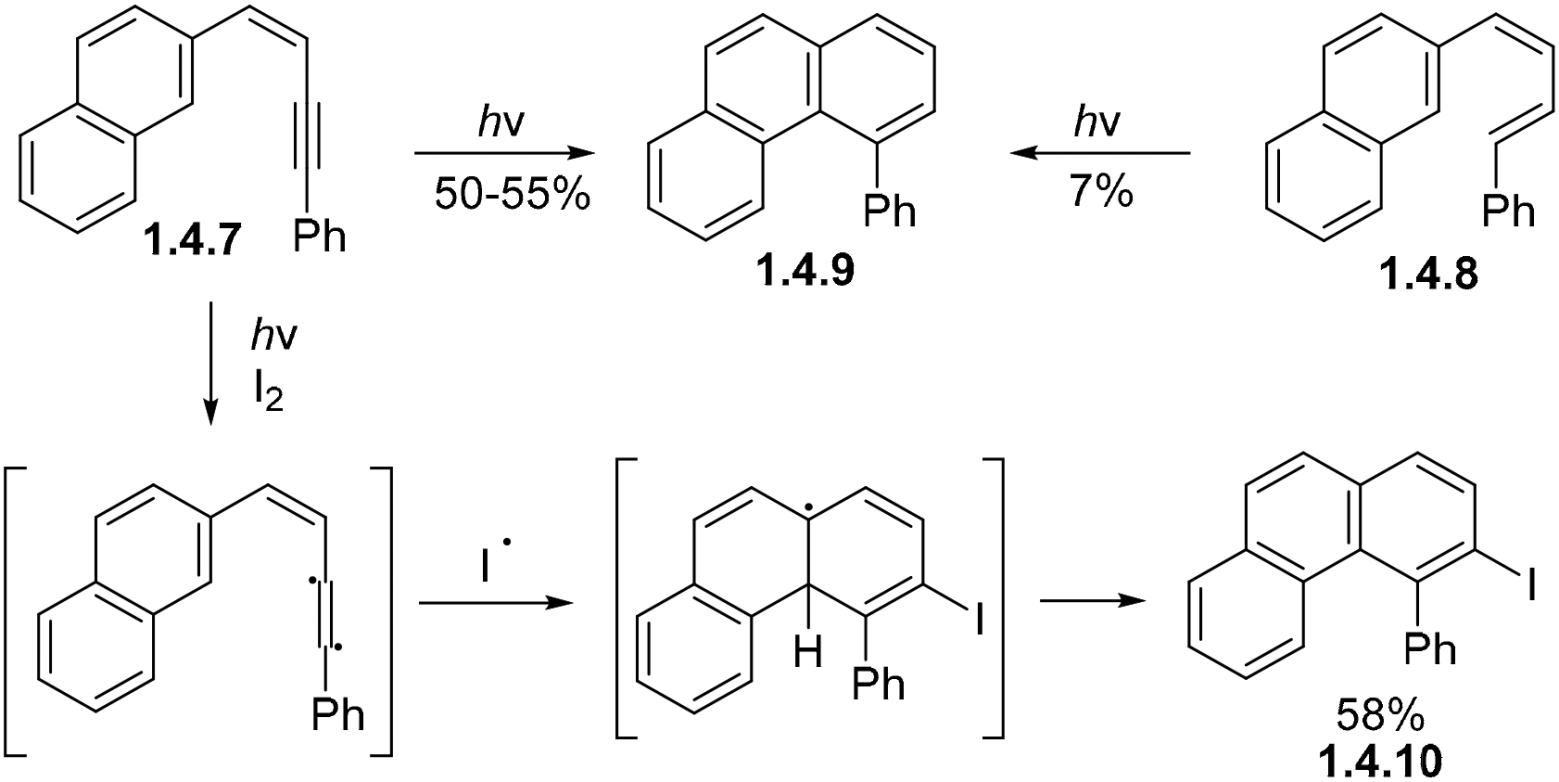
Comparison of naphthyl enyne and diene photocyclizations.

Enynes such as **1.4.7** and dienes such as **1.4.8** [79,84] can undergo non-oxidative photocyclization to give the same phenanthrene **1.4.9** product (Scheme 24). The cyclization of **1.4.7** produces **1.4.9** in a 50–55% yield under non-oxidative conditions, while the cyclization of **1.4.8** produces **1.4.9** in only a 7% yield under these same conditions. These enyne cyclizations are believed to proceed via a free-radical mechanism followed by hydrogen abstraction from the solvent. These reactions can lead to interesting products; for example, **1.4.7** gives **1.4.10** in 58% yield under oxidative conditions with iodine. This product is formed via a series of radical intermediates beginning at the triple bond [81].

**Scheme 25.**
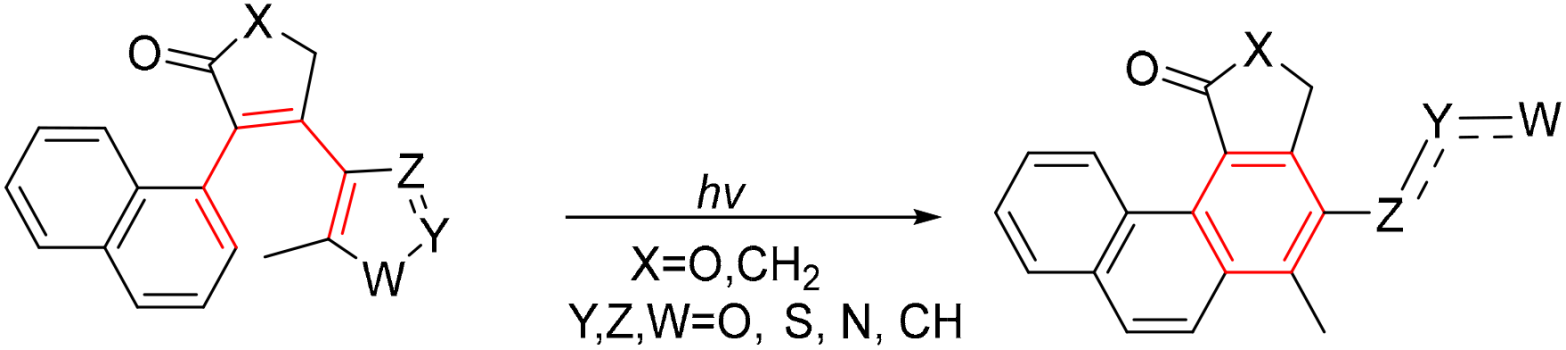
Phenanthrene formation via the photocyclizations of dienyl naphthalenes.

The annealing of a diene to naphthalene can lead to photochemical cyclizations forming phenanthrenes. This process occurs via 6-π electrocyclization forming a dihydrophenanhrene intermediate (Scheme 25). This intermediate is unstable and can either revert back to the starting napththalene, or in this case, it undergoes C–W bond cleavage heterolytically, with aromatization as a driving force [85]. This bond cleavage pushes the reaction in an irreversible direction, bypassing the need for an oxidant for aromatization.

**Scheme 26.**
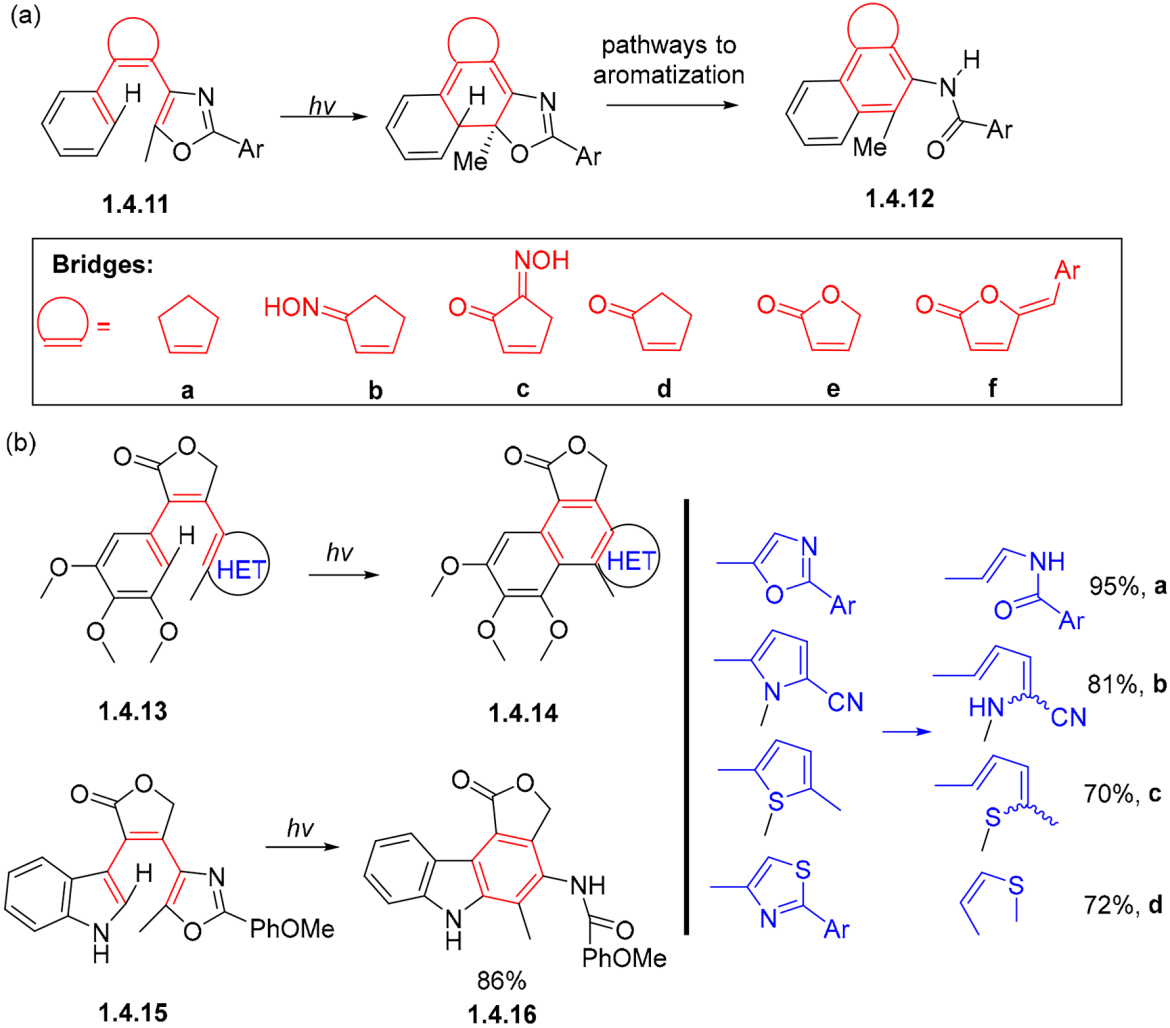
The introduction of heterocyclic units allows naphthalene and carbazole formation with rearomatization via C–X (where X=N, O, S) bond cleavage: (**a**) variations in the bridge moiety, (**b**) variations in the terminal hetaryl group.

A skeletal photorearrangement followed 6π-electrocyclization of heteroaromatic diarylethenes **1.4.11a–f** with various alkene substitutions. Interestingly, deprotonation was found to be the most efficient among the alternative aromatization pathways following 6π-electrocyclization. As the result, electronegative substitution next to the phenyl group reduced the reaction time and increased the product yield of substrates **1.4.12a–f** (Scheme 26a) [86]. This photorearrangement was later expanded to other heteroaromatic rings containing nitrogen, sulfur, and oxygen (**1.4.13a–d**, Scheme 26b) to form products **1.4.14a–d** in yields of 70–95% [87]. These photorearrangements also tolerate indoles such as **1.4.15**, leading to the formation of carbazole products **1.4.16** in an 86% yield.

**Scheme 27.**
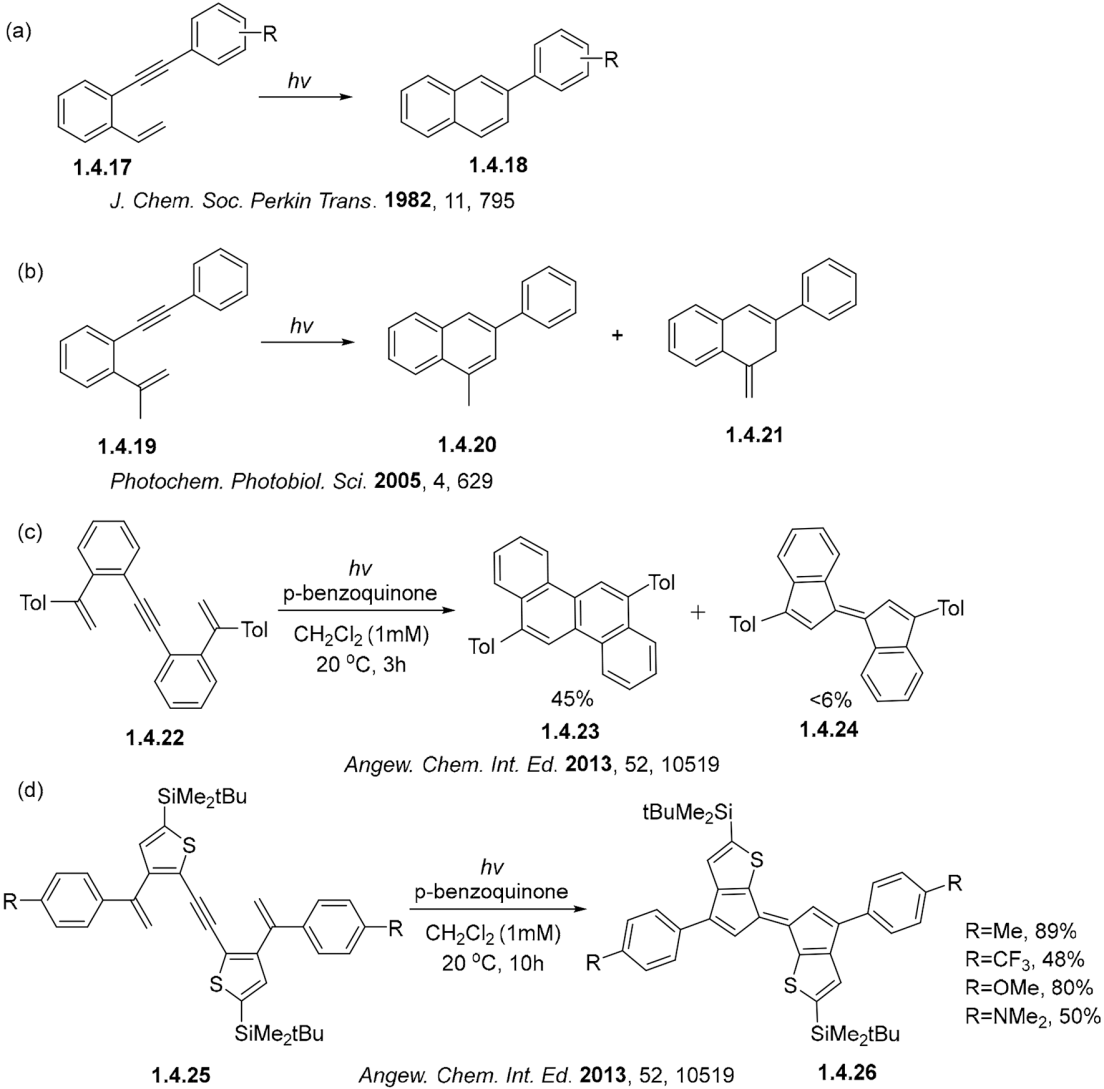
Comparison of regioselectivity of enyne photocyclizations: (**a**,**b**) Selective formation of six-membered cycles [89,91], (**c**) the first indications that five-membered ring formation is possible, (**d**) complete switch towards the C1–C5 cyclization [90].

The photocyclization of benzannelated enynes can occur via either C_1_C_5_– or C_1_C_6_– forming pathways. Many early examples demonstrated the exclusive formation of six-membered rings via C_1_C_6_ photocyclization; for example, the transformation of **1.4.17** to **1.4.18** (Scheme 27a), **1.4.19** to **1.4.20** and **1.4.21** (Scheme 27b), and **1.4.22** to **1.4.23** and **1.4.24** (Scheme 27c) [24,88-91]. Among the early reports, only one example of a C_1_C_5_ photocyclization in a thiophene-containing starting material, **1.4.25** to **1.4.26** (Scheme 27d), was described by Fukazawa and coworkers in [76].

**Scheme 28.**
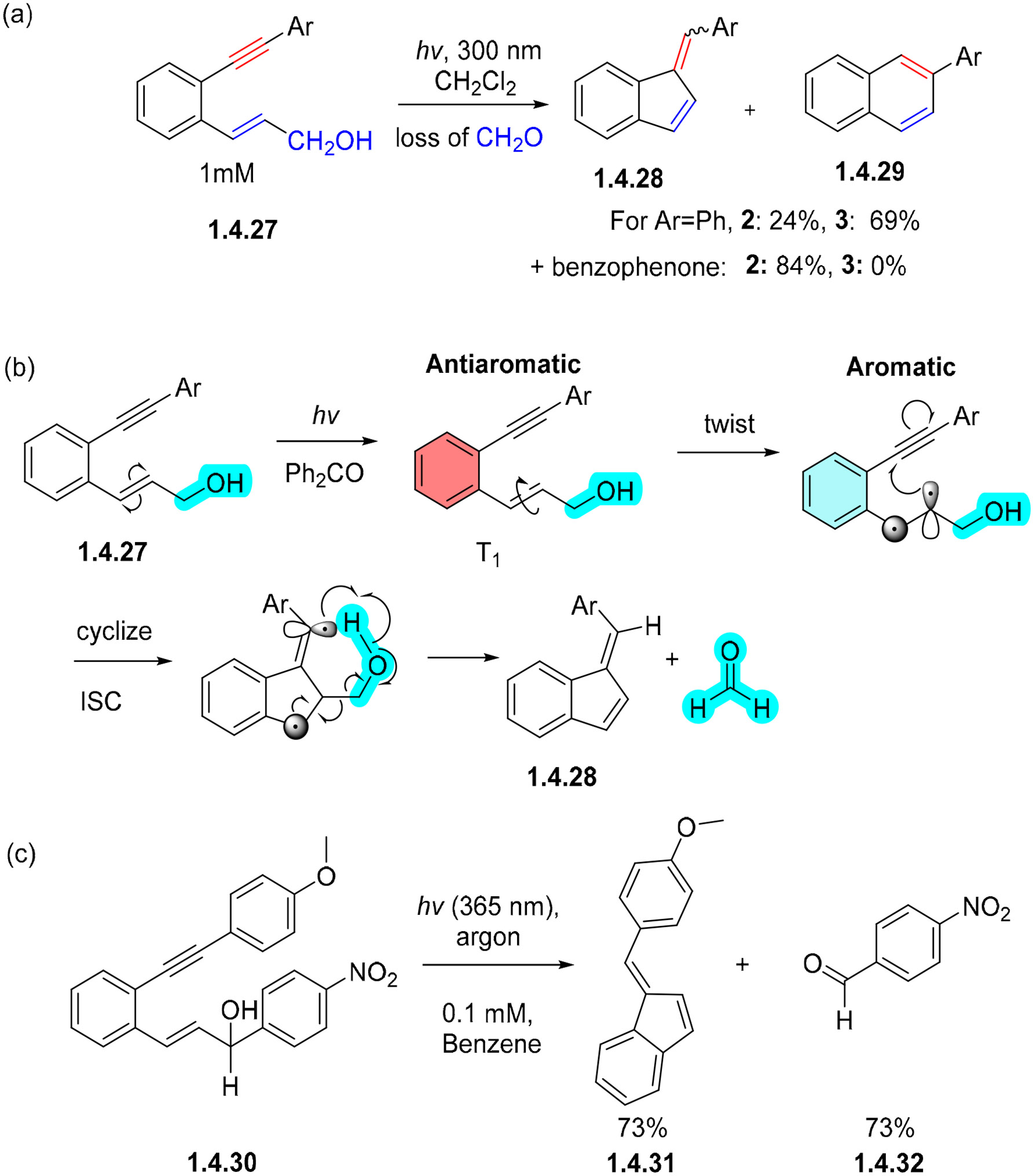
(**a**) Effect of triplet sensitization on the ratio of C1–C5 photocyclization of aromatic enynes. (**b**) Mechanism of C1–C5 cyclization with the loss of formaldehyde. (**c**) Enyne precursor releases an aldehyde via C1–C5 photocyclization.

Alabugin and coworkers found that the photochemical C1–C5 cycloaromatizations of benzannelated enynes can be efficient upon triplet sensitization with benzophenone. An example showing the transformation of **1.4.27** to **1.4.28** and **1.4.29** is shown in Scheme 28a [24]. Although the thermal C1–C5 cycloaromatization reaction of such enynes exhibits high activation barriers (>40 kcal/mol), photocyclization forming fulvenes can proceed readily in ambient conditions. Computational analysis revealed that, in the triplet state, the benzene fragment is antiaromatic. In order to escape antiaromaticity, the electrons in the π bond of the alkene decouple, allowing the alkene moiety to twist. This twist causes the benzene fragment to regain its aromatic character within the excited state. This new enyne photocyclization found an interesting application in the photochemical uncaging of formaldehyde, aldehydes, and ketones. For example, the C1–C5 photocyclization of substrate **1.4.30** simultaneously releases fulvene **1.4.31** and aldehyde **1.4.32,** both in a 73% yield (Scheme 28b) [92]. The release of formaldehyde is promising for applications in biology [93].

**Scheme 29.**
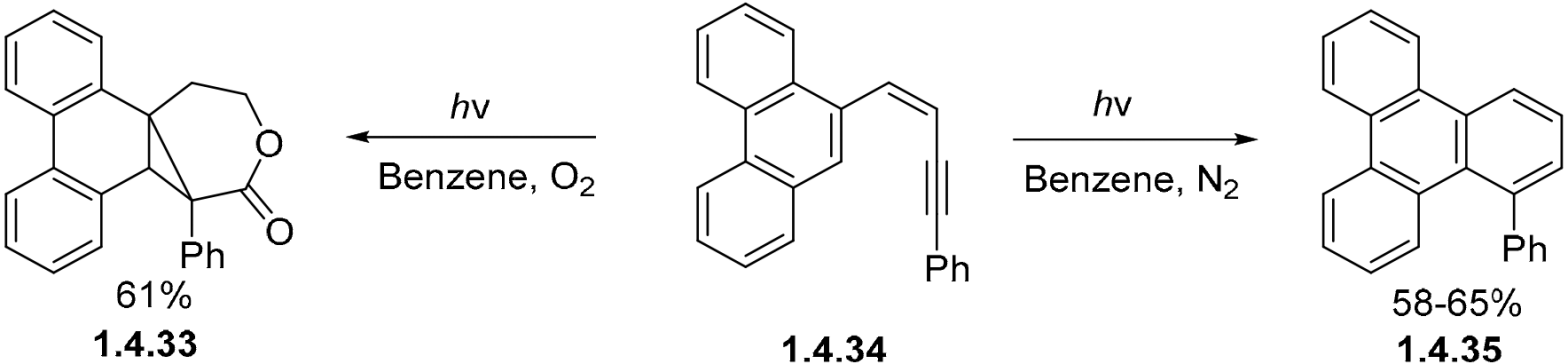
(**Left**) Enyne photocyclization diverged into seven-membered ring formation via the addition of O_2_. (**Right**) Enyne cage precursor releases aldehyde via non-oxidative photocyclization.

While the cyclization of enyne **1.4.33** (Scheme 29) in benzene under a nitrogen atmosphere gives the expected 1-phenyltriphenylene product **1.4.34** in good yields, another unconventional product is formed for the same enyne when reacted under oxidative conditions with oxygen, which form predominantly lactone **1.4.35** in 61% yield [94]. In this instance, cyclization can occur via either C1–C5 or C1–C6 pathways [24].

#### 1.5. Alkene Photocyclizations Involving Biphenyl and Phenanthrene Systems

**Scheme 30.**
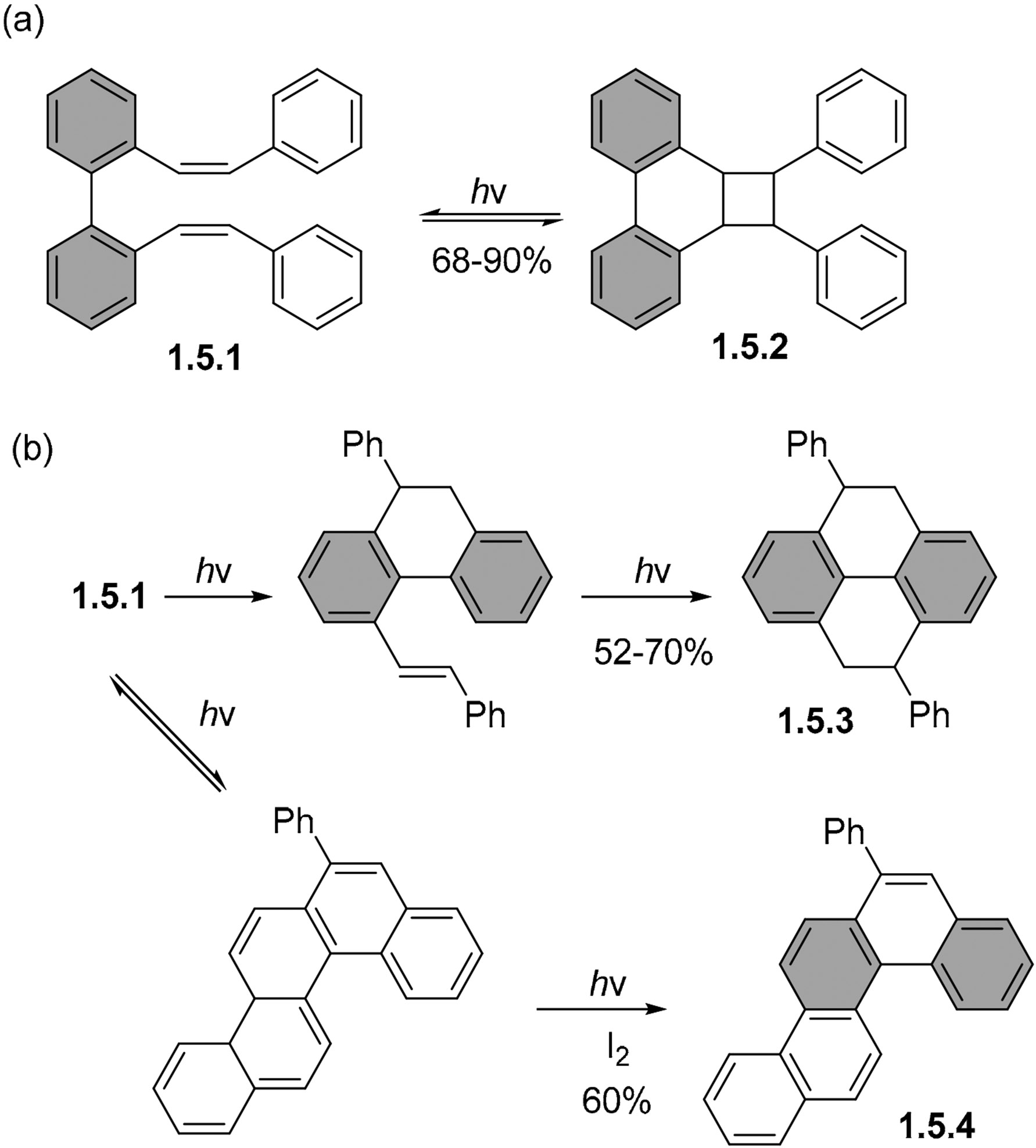
Changes in products in the photocyclization of 2,2′-bis-styrylbiphenyl (300 nm irradiation, quartz vessel, deaerated hexanes). Irradiation times: 15 min (**top/a**), 6 h (**center/b**) and 8 h (**bottom/b**) in the presence of one equivalent of iodine).

2,2′ -Distyrylbiphenyl **1.5.1** can undergo several possible transformations, with selectivity determined by reaction conditions (Scheme 30a). The short irradiation of this substrate under non-oxidative conditions gives predominantly the product of a [2 + 2] cycloaddition (**1.5.2**) between the double bonds of the styryl groups in up to 90% yields.

Upon the longer irradiation of **1.5.1**, tetrahydropyrene **1.5.3** becomes the major observable product in up to 70% yields (Scheme 30b). These results suggest the reversibility of the strained [2 + 2] product formation, which allows the more stable tetrahydropyrene to become the final product after photoequilibration. The partially closed tetrahydrophenanthrene intermediate was shown to form the same final product tetrahydropyrene **1.5.3** [68,95]. However, the photocyclization of the same bis-stilbene in the presence of iodine gives predominantly the chrysene product **1.5.4** in a 60% yield. The different regioselectivity of the ring closure and the lack of pyrene product under these conditions suggest that the mechanism of these photochemical transformations is likely to be quite complex. In addition to the reversibility of the cyclization steps, the oxidant is certainly involved in the reaction mechanism by intercepting the partially aromatized intermediates.

**Scheme 31.**
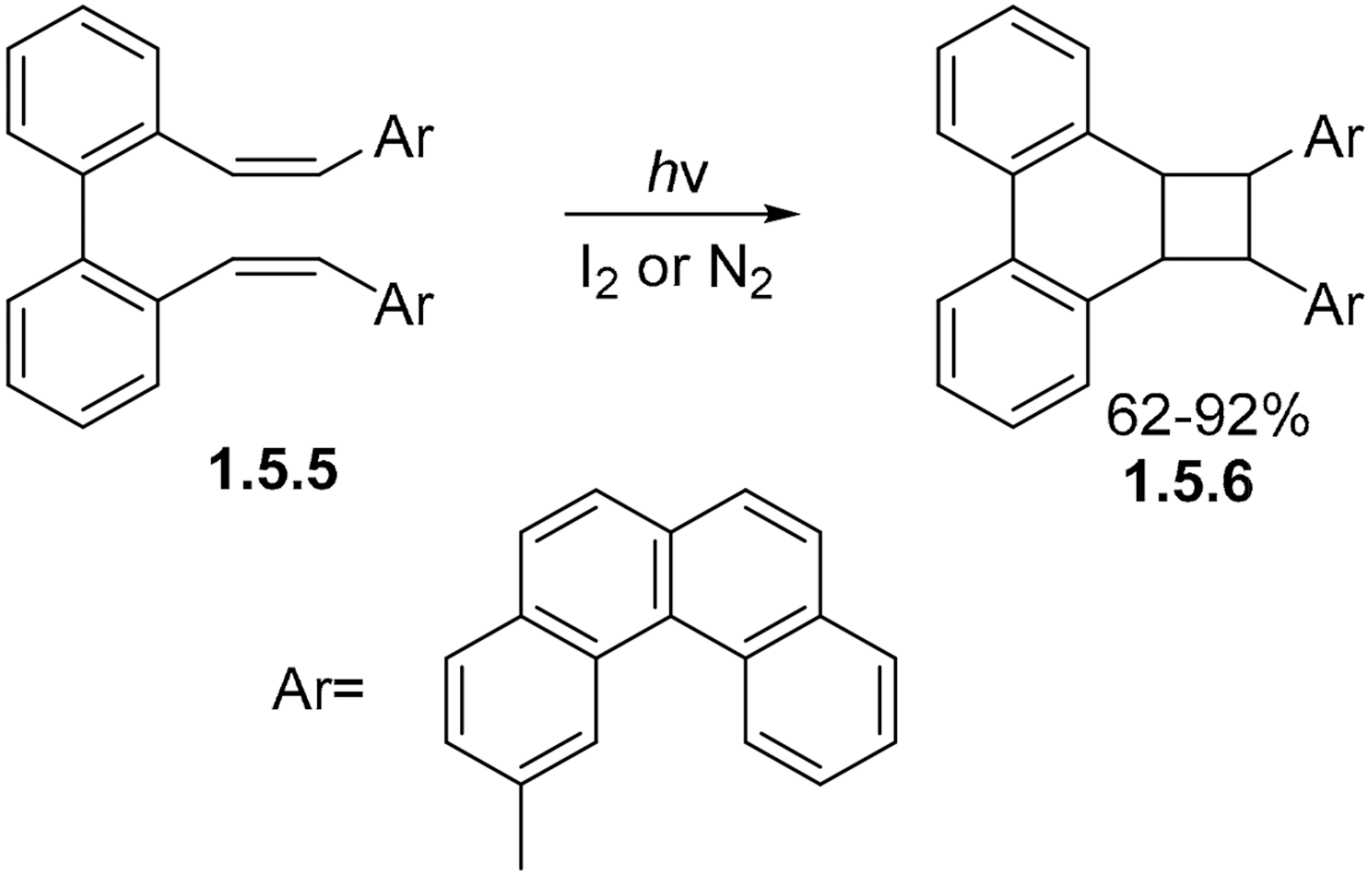
[2 + 2] cycloaddition in photocyclization of a biphenyl stilbene analog.

The change of the terminal Ph groups to a bulkier helicene moiety in the biphenyl bis-styrene **1.5.5** disfavors most of the above cyclizations, and only the product of [2 + 2] cycloaddition **1.5.6** is observed under both oxidative and non-oxidative conditions upon irradiation in a quartz vessel by a 300 nm lamp in deaerated hexanes under nitrogen (Scheme 31) [68,95]. However, only relatively short irradiation times (1 h) were applied. This short irradiation time favors the formation of the kinetic product.

**Scheme 32.**
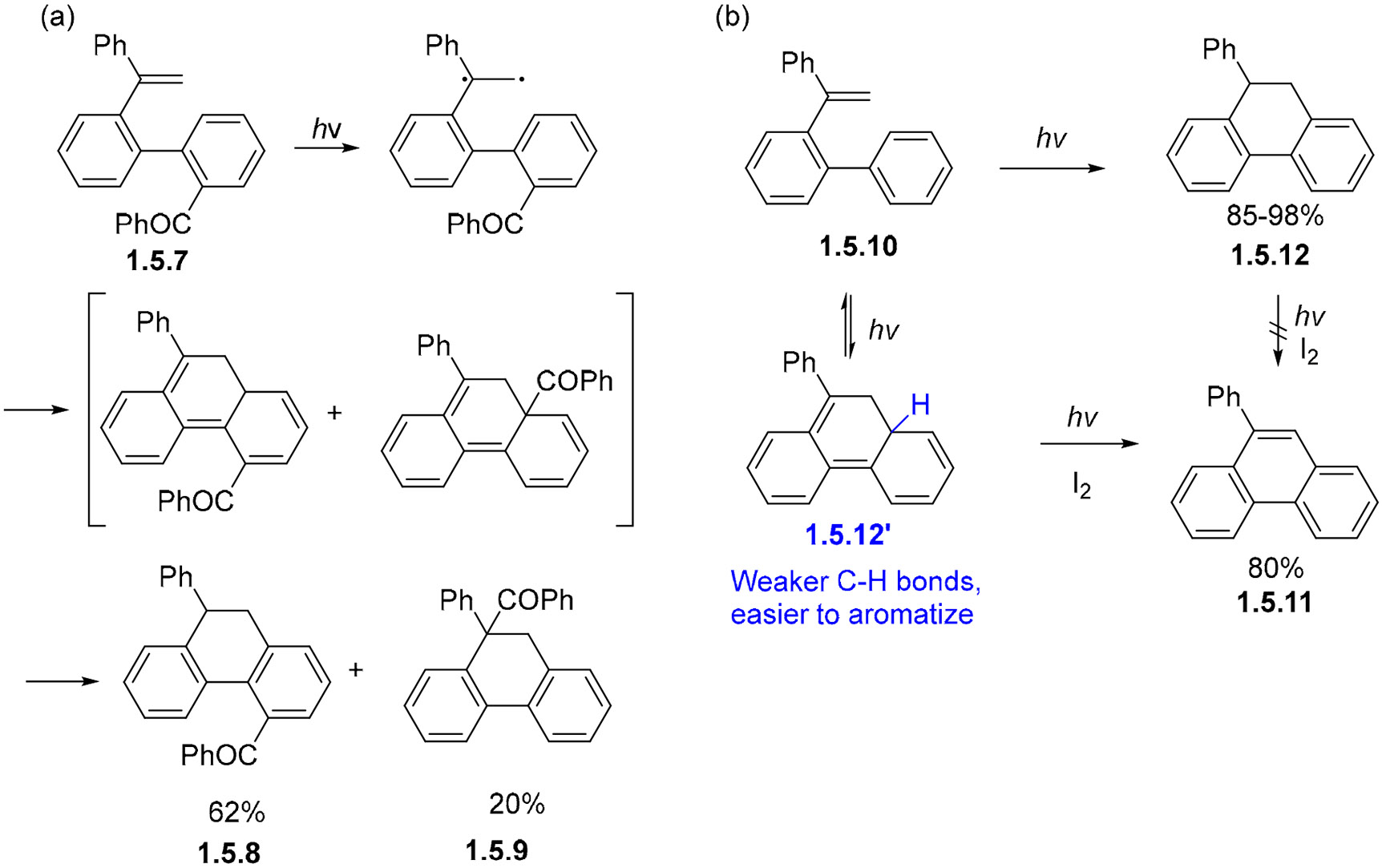
The photocyclization of 1-styryl biphenyls can lead to either partially (**a**) or fully (**b**) aromatized products.

If the phenyl group in styryl biphenyl substrates moves to the internal alkene carbon, such as **1.5.7**, non-oxidative photocyclization gives isomeric 9,10-dihydrophenanthrenes **1.5.8** and **1.5.9** (Scheme 32a) [96]. These products are believed to be formed via triplet excitation. Interestingly, although the oxidative photocyclization of **1.5.10** leads to phenanthrene product **1.5.11**, an attempt to aromatize the dihydro-phenanthrene **1.5.12** product of the non-oxidative cyclizations failed to proceed to fully oxidized products (Scheme 32b) [97-99]. This observation suggests that the C–H bonds in the pre-aromatized intermediate **1.5.12**′ are weaker than in the isomeric dihydro-phenanthrene **1.5.12**.

**Scheme 33.**
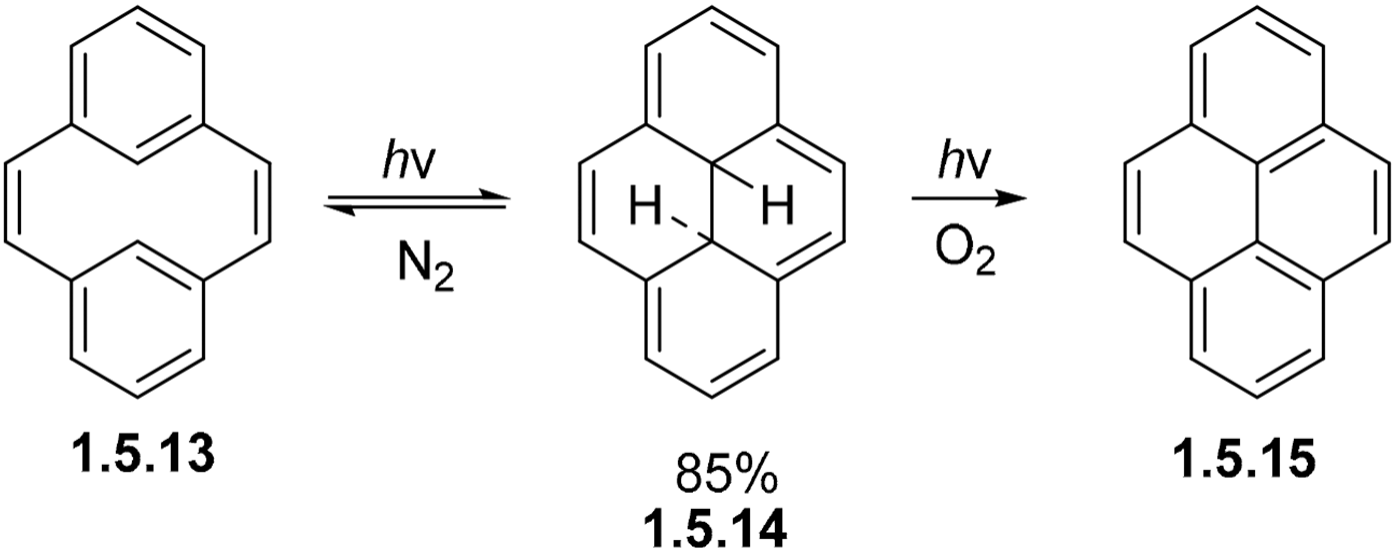
Pyrene ring formation via the photocyclization of [2,2]metacyclophane.

One of the few de novo ways to synthesize pyrenes is shown below. By irradiating metacyclophane **1.5.13** in the absence of oxidants, it is transformed into dihydropyrene **1.5.14**, which can then be purified and subjected to oxidative conditions to quantitatively give pyrene **1.5.15** in overall good yields (Scheme 33) [100]. This cyclization is mechanistically interesting due to its electronic duality: it can be considered as either a constrained version of the stilbene cyclization or an electrocyclization of a larger annulene. Theoretical analysis of the reactive excited state is needed to distinguish between these possibilities. We also would like to recognize the work of professor Michell, for his immense contributions in the field of photochemistry and the cyclizations of metacyclophanes [101].

**Scheme 34.**
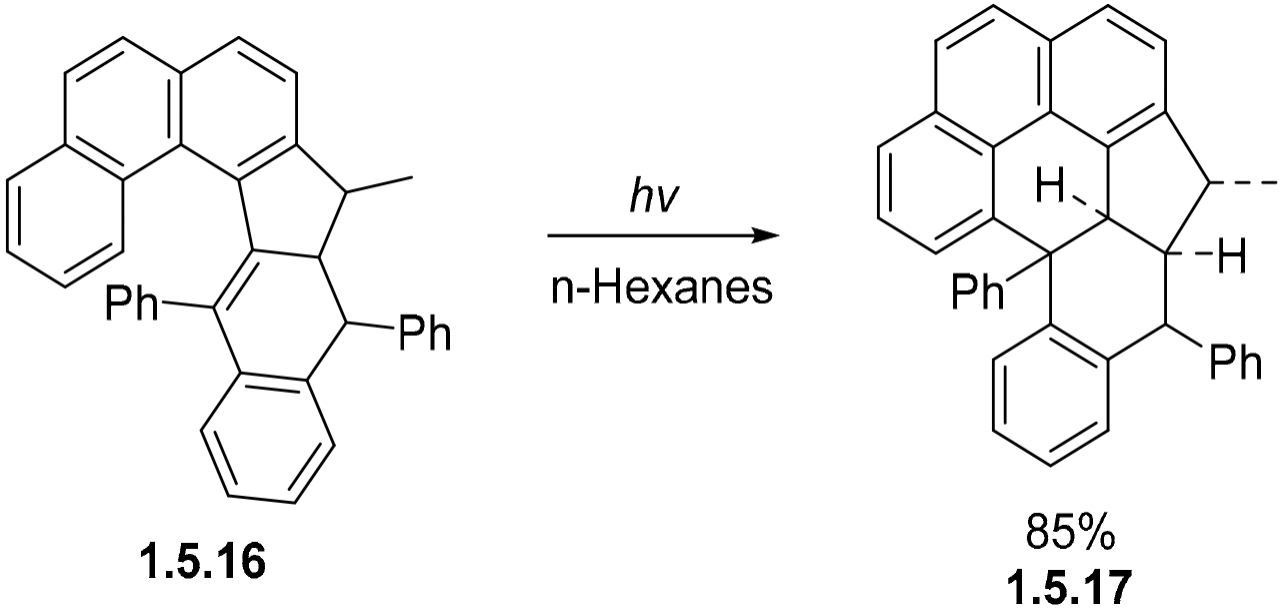
Non-oxidative photocyclization at the bay region of phenanthrene in the absence of aromatization.

The photochemical cyclization of 4-styrylphenanthrene provides another example of alkene addition to the bay region of phenanthrene. The reaction proceeds upon exposure to 310 nm light in 85% yield [102]. In this example, even a strained polycyclic system undergoes photocyclization. As with other examples (Schemes 30 and 33), the photocyclization of **1.5.16** to pyrene core **1.5.17** succeeds in forming a fully cyclized product only under non-oxidative conditions (Scheme 34). Oxidation to a fully aromatized pyrene is blocked by the strain and the Ph group at the site of cyclization.

Intrigued by the synthetic potential of cyclizations of alkenes in the bay region of phenanthrene as a potential synthetic approach to pyrenes, Dos Santos et al. more deeply explored this cyclization with the goal of expanding the list of reactions capable of forming the pyrene core de novo [95,96,103-112].

The photochemistry of the parent version of this system has been explored by Laarhoven et al. [76] and Morgan et al. [113], who found that the main product in this system was chrysene, produced via a sequence of two Mallory cyclizations. Interestingly, a pyrene product was also detected, but only in a trace amount.

**Scheme 35.**
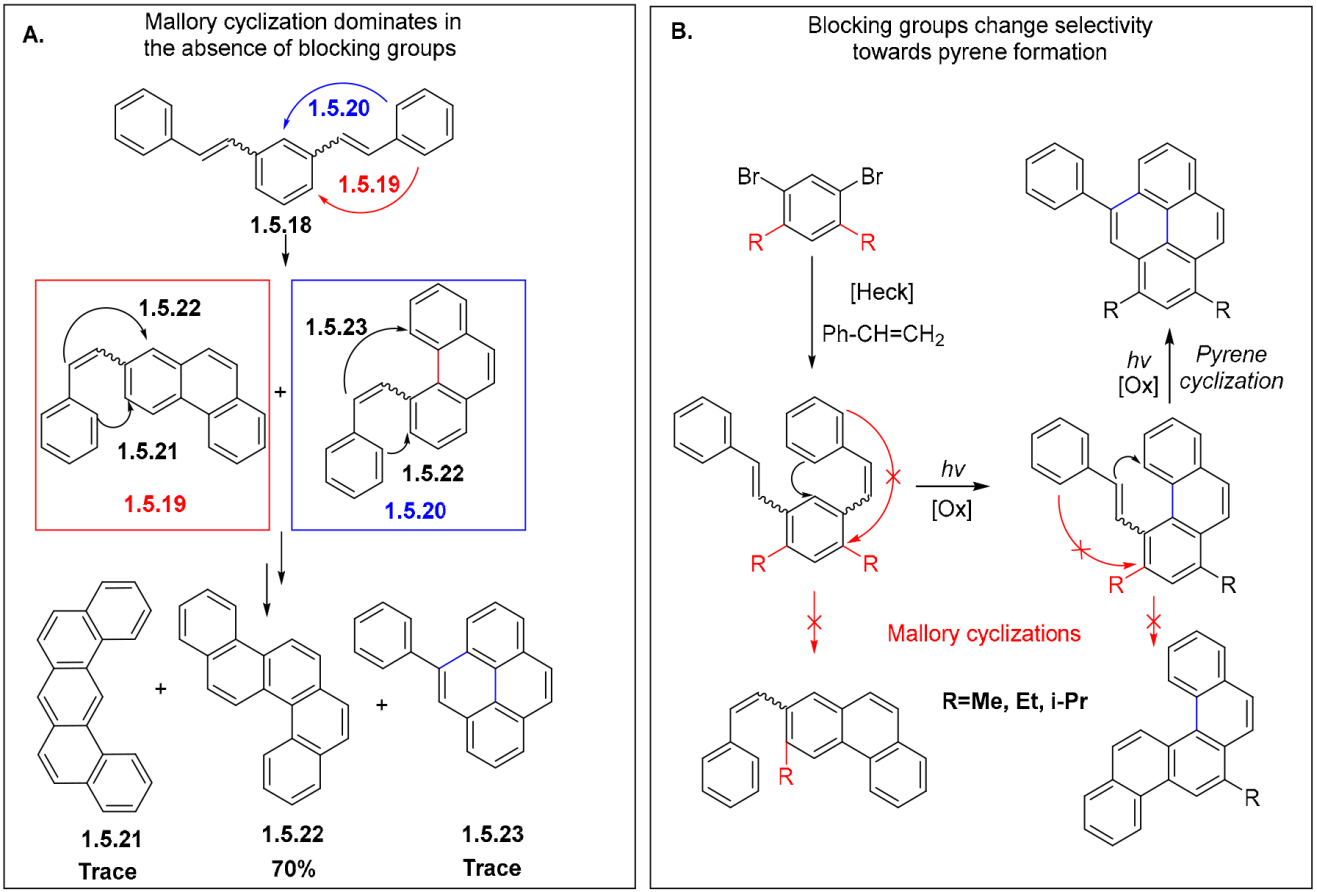
(**A**) Possible pathways of the photocyclization of bis-stilbenes reported by Laarhoven et al. [76,113] and Morgan et al. [19]. (**B**) The blocking group strategy for the selective synthesis of pyrenes. Note the dual role of blocking groups in the control of Mallory cyclization: directing regioselectivity in the 1st step and preventing this cyclization in the 2nd step.

These results illustrate the main challenge in diverting photochemistry of bis-stilbenes from the double Mallory cyclization towards the formation of non-symmetrical pyrenes (Scheme 35).

**Scheme 36.**
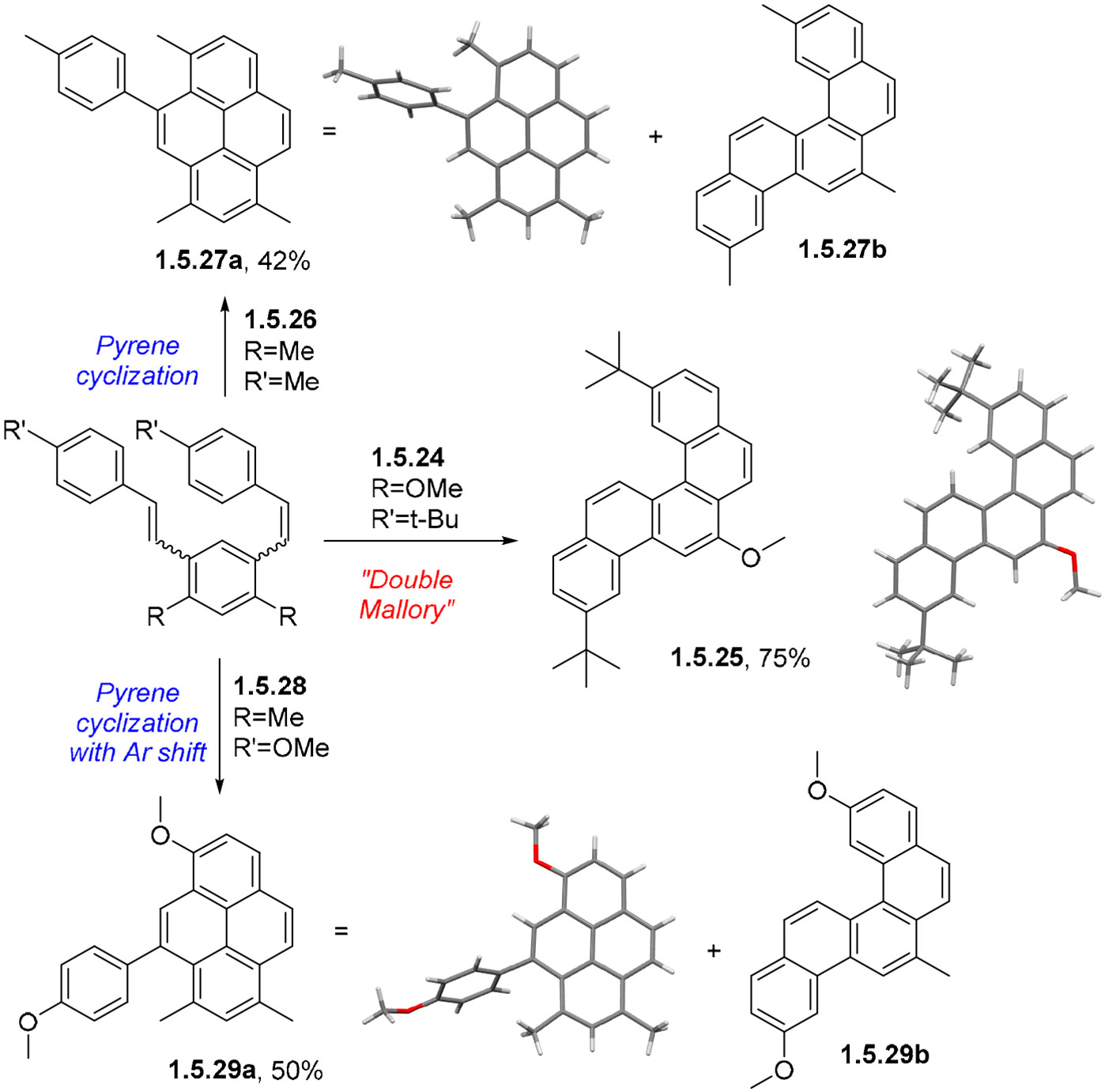
Initial results of bis-stilbene photocyclizations yielding pyrene and chrysene products (adopted with permission from [114]).

The cyclization of 1,3-distyrylbenzene **1.5.18** can follow several pathways. In the first step, there are two possible stilbene photocyclizations (i.e., the Mallory reactions), which could proceed in different directions towards **1.5.19** and **1.5.20** (Scheme 36). The second step can be either another Mallory reaction or cyclization in the bay area of phenanthrene to give products **1.5.21**, **1.5.22**, and **1.5.23**.

Dos Santos et al. [114] found that the competition between the Mallory reaction and the alkene cyclization in the phenanthrene bay region can be controlled by strategically installing alkyl groups to disfavor the Mallory cyclization. This targeted modification at the bay region forces these substituted phenanthrenes to follow the new mechanistic pathway towards pyrenes.

The photoreaction of a bis-OMe-substituted bis-stilbene **1.5.24** (1 equiv [1.5 mM]) in the presence of I2 (3 equiv.) and propylene oxide (100 equiv.) in quartz glassware with 254 nm excitation produces the double Mallory product **1.5.25** in a 75% yield [114]. On the other hand, a dimethyl-substituted stilbene **1.5.26** formed the desired pyrene **1.5.27a** as the major product, isolated in 42% yield (Scheme 36). The double Mallory product **1.5.27b** formed with the loss of one of the Me groups was identified as a minor product. The formation of pyrene was favored in a 5:2 **1.5.27 a:b** ratio.

Another interesting result was observed for the photochemical transformation of a bis-stilbene reactant **1.5.28** with a *p*-OMe-substituted terminal aryl group. In this case, the formation of the final pyrene product **1.5.29a** included a 1,2-shift of the anisole moiety along the pyrene’s K-region. The cascade proceeded in a 50% yield with 5:1 selectivity relative to the double Mallory product (**1.5.29 a:b**) (Scheme 36).

**Scheme 37.**
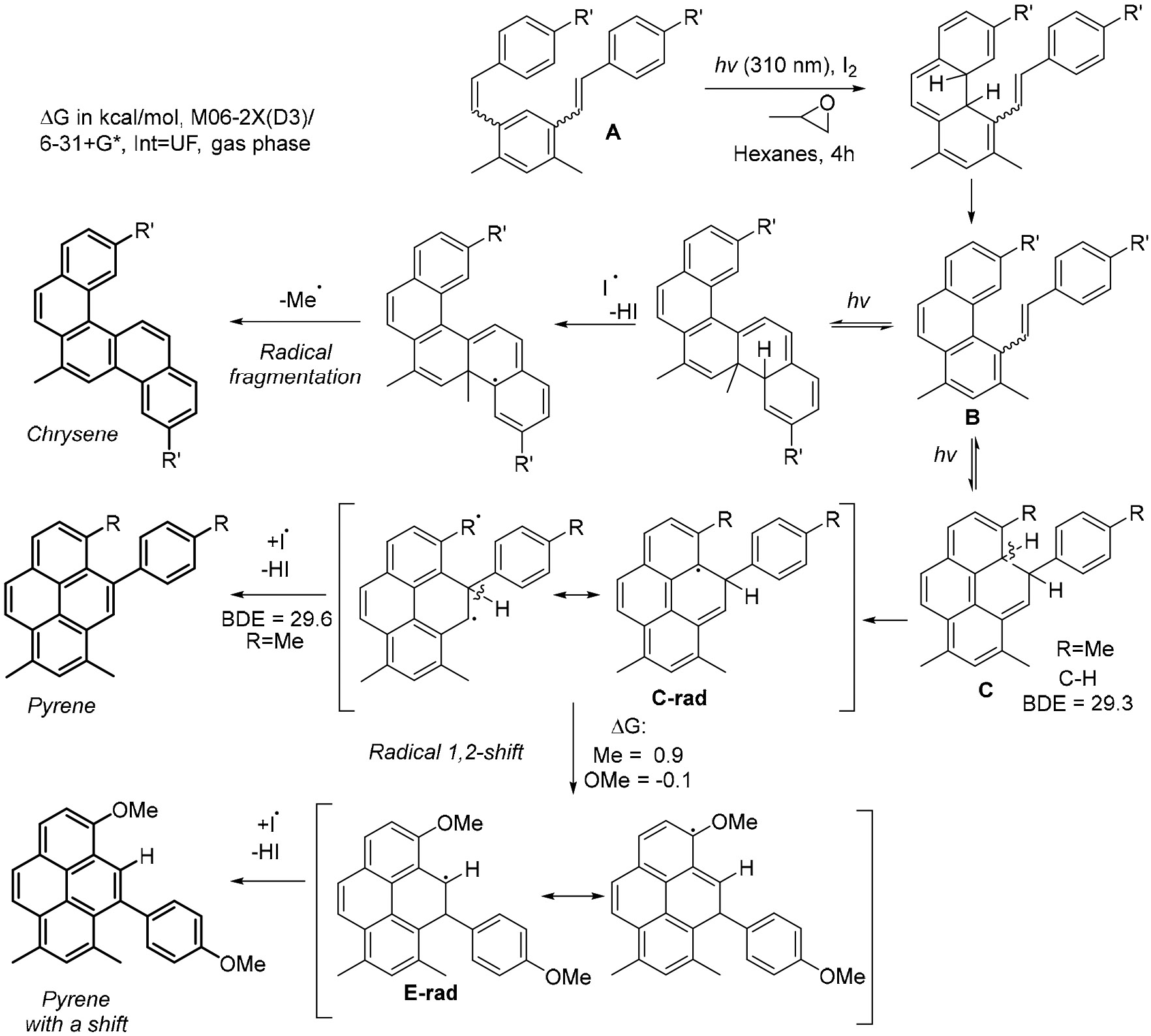
Presence of weak C–H bonds lead to a cross-over from the photochemical to the radical mechanism.

An even more interesting result was observed for the photochemical transformation of a bis-stilbene reactant with a *p*-OMe-substituted terminal aryl group. In this case, the formation of the final product **F’** included a 1,2-shift of the anisole moiety along the pyrene’s K-region (Scheme 37). The cascade proceeded with a 50% yield, with even better (5:1) selectivity relative to the double Mallory path. This intriguing result indicates the presence of interesting electronic factors that were absent from stilbenes where R = alkyl groups.

The observation of Ar-migration products revealed an interesting interplay between photochemical and radical processes involved in the oxidative arene-alkene photocyclizations. The assistance by radical species is important because two C–H bonds in the dearomatized intermediates are very weak (BDE = 29 and 51 kcal/mol) and can be abstracted by the I- and O-centered radicals. A refined mechanistic picture emerges from the fusion of the photochemical and radical pathways illustrated in Scheme 37.

Furthermore, radical H-atom transfer at the weakest C–H bond leads to radical **E-rad** with the spin density at the perfect location for promoting the observed Ar migration via a radical 1,2-Ar shift. This scenario provides a logical explanation of why the Ar shift is observed for Ar = *p*-Ph-OMe. Not only does the OMe group in the migrating Ar group provide kinetic stabilization to the migrating TS, but the OMe group in the polycyclic core is at the right position to stabilize the delocalized radical in the product. These two effects support experimental observations of aryl migration when R = OMe vs. the absence of migration when R = Me, Cl, H. For R = *t*-Bu, migration is driven by the release of steric bulk, suggesting that not only product stabilization but reactant destabilization can also be used to promote the 1,2-aryl shift.

Another piece of evidence supporting a radical mechanism is the observed loss of alkyl blocking groups. The driving force for radical C-R fragmentation increases with the stability of radical R (*t*-Bu > *i*-Pr > Et > Me). This step, which renders the second Mallory reaction irreversible, is more favorable for the more substituted radicals. This factor counterbalances the blocking group’s size’s effect on the competition between the Mallory cyclization and the “pyrene” cyclization.

#### 1.6. Heteroaromatic Stilbene Analogs

**Scheme 38.**
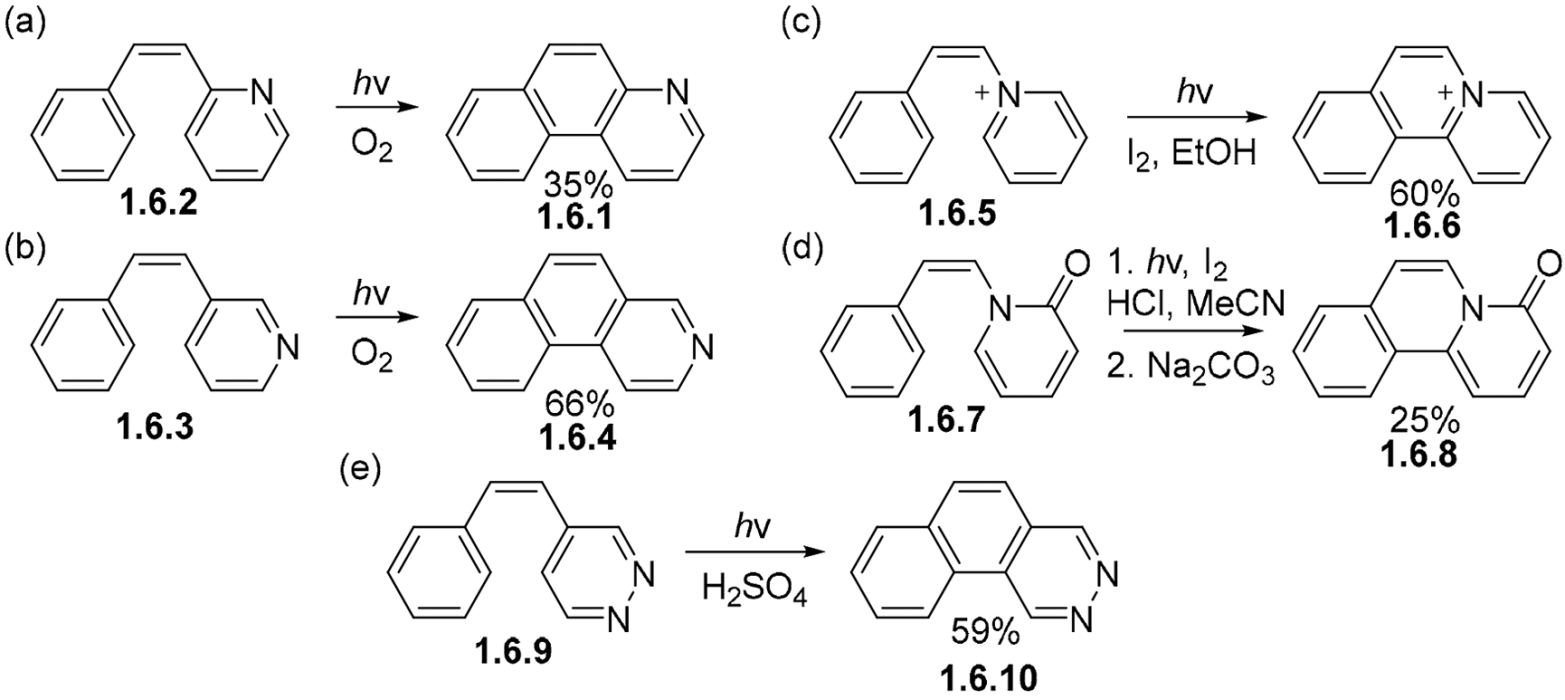
Photocyclizations of N-substituted stilbene analogs. (**a**) photocyclization of 2-azastilbene **1.6.2**. (**b**) photocyclization of 2-azaphenanthrene **1.6.3**. (**c**) photocyclization of 1-styrylpiridinium cation **1.6.5**. (**d**) photocyclization of styryl pyridine **1.6.7**. (**e**) photocyclization of diazastilbene **1.6.9**.

The photocyclizations of azastilbenes proceed similarly to their carbocyclic counterparts. The yields are often moderate and sensitive to the position of the embedded N atom. For example, the formation of 1-azaphenanthrene **1.6.1** from 2-azastilbene **1.6.2** proceeds in a much higher yield (66%, Scheme 38a) [115,116] than the conversion of 3-azastilbene **1.6.3** to 2-azaphenanthrene **1.6.4** (35%, Scheme 38b) [116-119].

Furthermore, nitrogen-containing analogs of stilbene often require a suitable excited state be involved in order to achieve the desired cyclization products. For example, while 1-styrylpiridinium cation **1.6.5** is readily converted to phenanthrinium cation **1.6.6** (Scheme 38c) [120], the styryl pyridone **1.6.7** remains unreactive unless it is converted to its protonated form **1.6.8** with the addition of HCl (Scheme 38d) [121]. Similarly, while the photocyclization of diazastilbene **1.6.9** fails, the desired transformation can be achieved in acceptable yields (59%) by irradiation in sulfuric acid to give diazophenanthrene **1.6.10** (Scheme 38e) [122]. This is attributed to the ^1^n, π* state being lower in energy than the ^1^π,π* state in some of the N-substituted substrates [123]. Protonation of nitrogen deactivates the undesired ^1^n, π* state and directs photoreactivity towards the cyclization path.

**Scheme 39.**
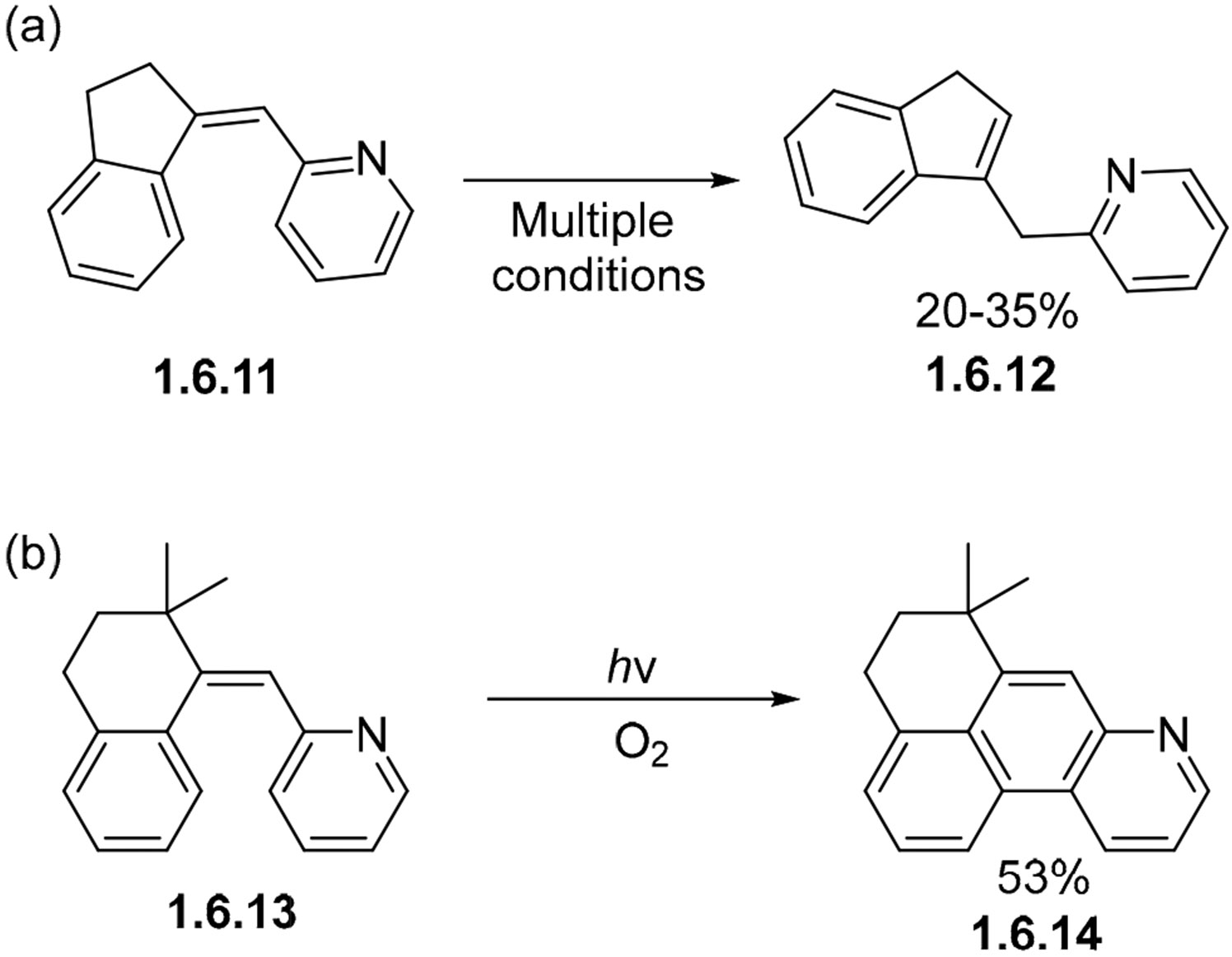
Sufficient strain can impede stilbene photocyclization. (**a**) 5—member ring induces high strain in photocyclization lowering yields. (**b**) 6—member ring does not induce high strain leading to higher yields than 5—member ring counterpart.

It was also found that such oxidative cyclizations fail in constrained systems where there is poor orbital overlap of the opposing aryl rings. For example, constrained exocyclic indane **1.6.11** does not cyclize, but forms an isomerized endocyclic indane **1.6.12** (Scheme 39a) in a 20–35% yield under various photochemical conditions. The strain imposed by the five-member ring prevents the formation of a phenanthrene unit. In contrast, a more flexible six-membered analog **1.6.13** succeeds in forming a fully cyclized product **1.6.14** (Scheme 39b) [115]. The larger ring size in the left aromatic unit allows for favorable orbital overlap during the cyclization, leading to the formation of the desired product.

**Scheme 40.**
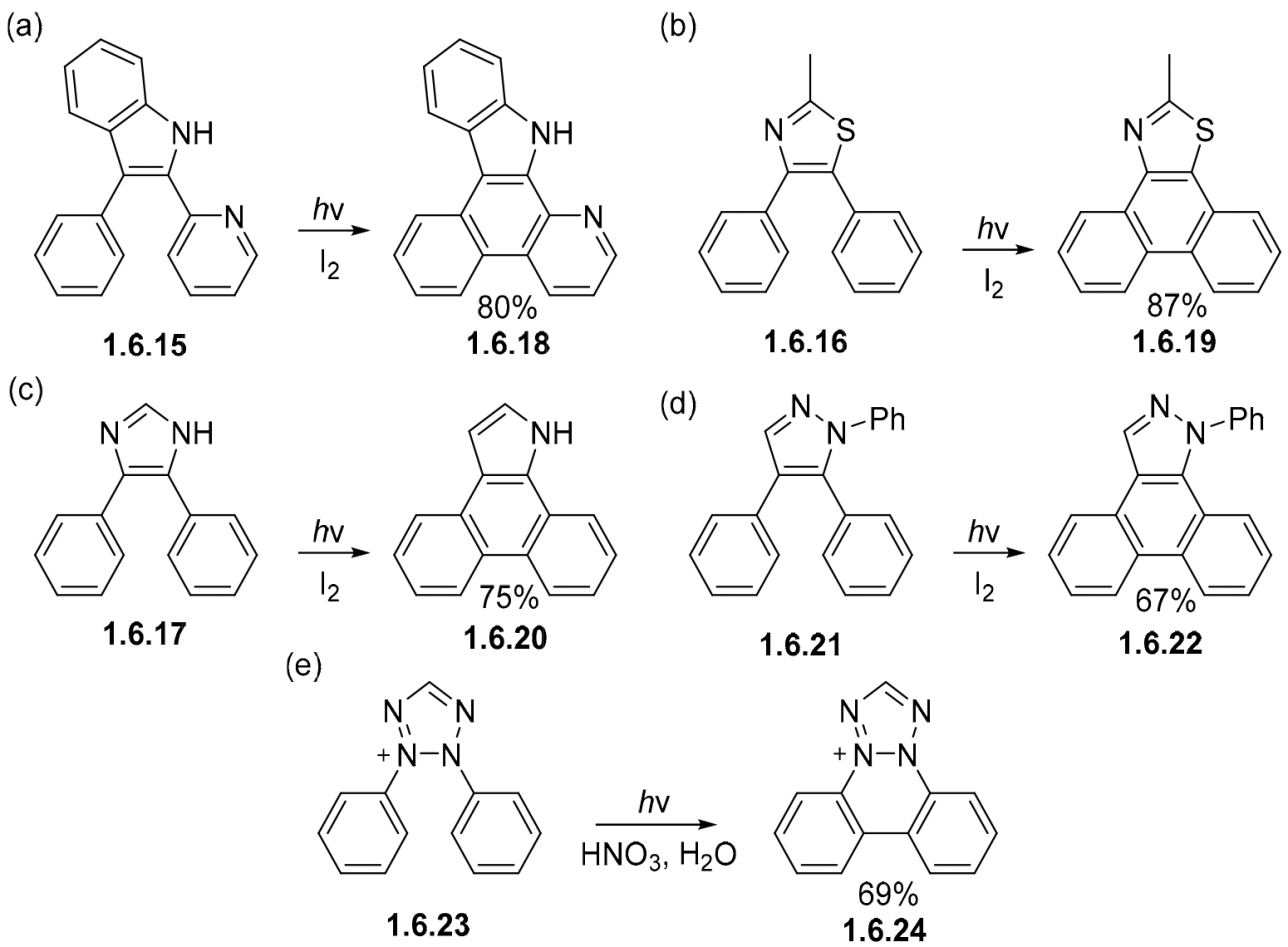
Heteroaromatic rings in the bridge do not impede photocyclization in similar nitrogen containing substrates. (**a**–**e**) Photocyclizations of complex heteroaromatic substrates containing varying numbers of N- and S-atoms.

These oxidative photocyclizations are versatile, and succeed in producing even more complex heteroaromatic substrates with varying numbers of N- and S- atoms. This is illustrated by the high-yielding transformations of **1.6.15** [124], **1.6.16** [125], and **1.6.17** [125] to the cyclic products **1.6.18**, **1.6.19**, and **1.6.20** (Scheme 40a-c). Although 1,3,4-triphenylpyrazoles are not compatible with these conditions, their 1,4,5-counterpart successfully participates in the cyclization of **1.6.21** to **1.6.22** achieved in a 67% yield (Scheme 40d) [126]. Similar to other nitrogen-containing substrates, the photocyclization of diphenyltetrazolium ion **1.6.23** readily proceeds in the presence of acids to **1.6.24** in a 69% yield (Scheme 40e) [127-129].

#### 1.7. Photocyclizations Involving Pyrroles, Indoles, and Other Five-Membered Rings

**Scheme 41.**
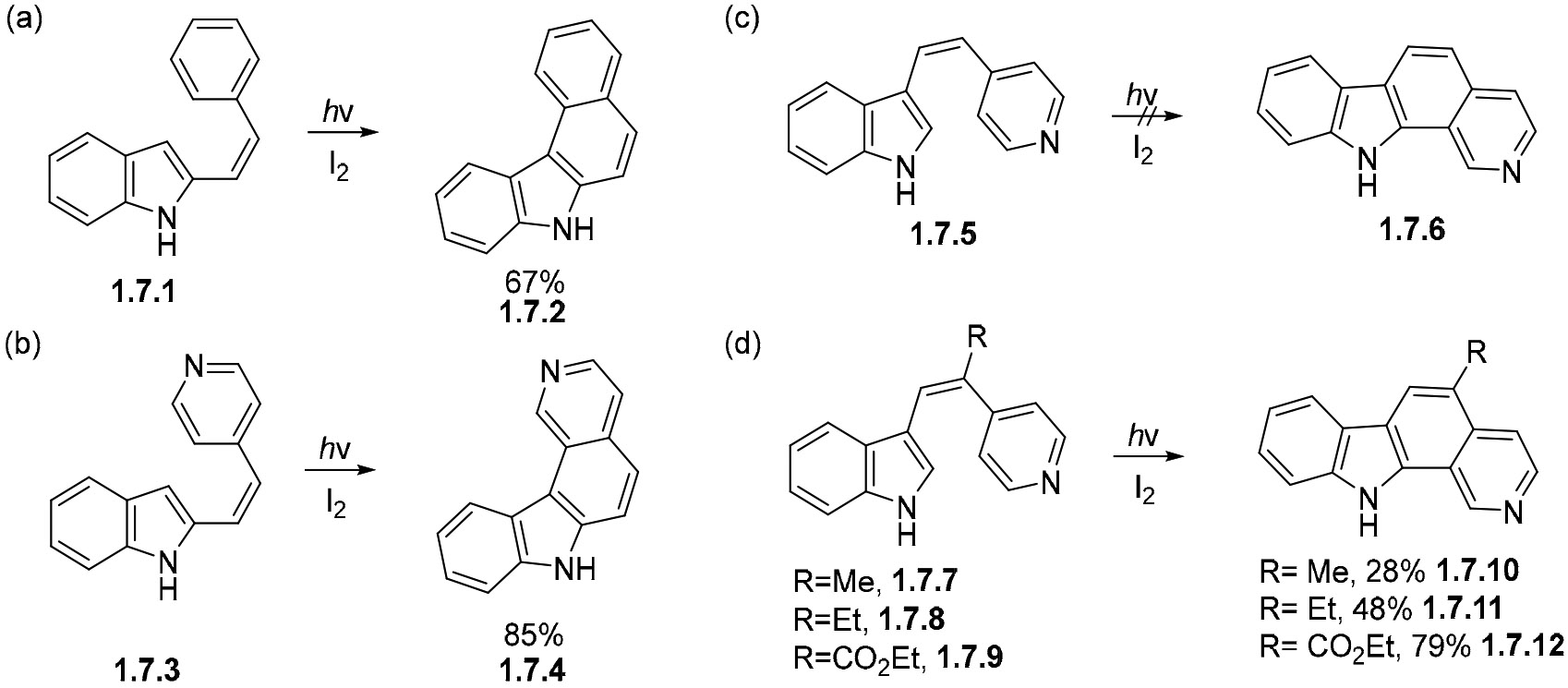
(**a**–**d**) Photocyclizations of selected styrylindoles under oxidative conditions.

The photocyclization of styrylindoles offers an interesting approach to the formation of benzocarbazoles. As an example, the photocyclization of indole **1.7.1** readily forms benzocarbazole **1.7.2** in a 67% yield under oxidative conditions (Scheme 41a) [130]. The formation of pyridylcarbazole is also possible via this path, as illustrated by the conversion of **1.7.3** into carbazole **1.7.4** in an 85% yield (Scheme 41b) [131]. However, the position to the ethenylpyridine group in the carbazole plays an important role, as illustrated by the failed photocyclization of indole **1.7.5** to pyridylcarbazole **1.7.6** (Scheme 41c) [131]. This seemingly inherent inability to cyclize under oxidative conditions is circumvented by using 1-substituted ethenylpyridines. The cyclizations of methyl, ethyl, and carbethoxy derivatives **1.7.7**, **1.7.8**, and **1.7.9** proceed to form their corresponding pyridylcarbazoles **1.7.10**, **1.7.11**, and **1.7.12** in 28%, 48%, and 79%, respectively (Scheme 41d) [131].

**Scheme 42.**
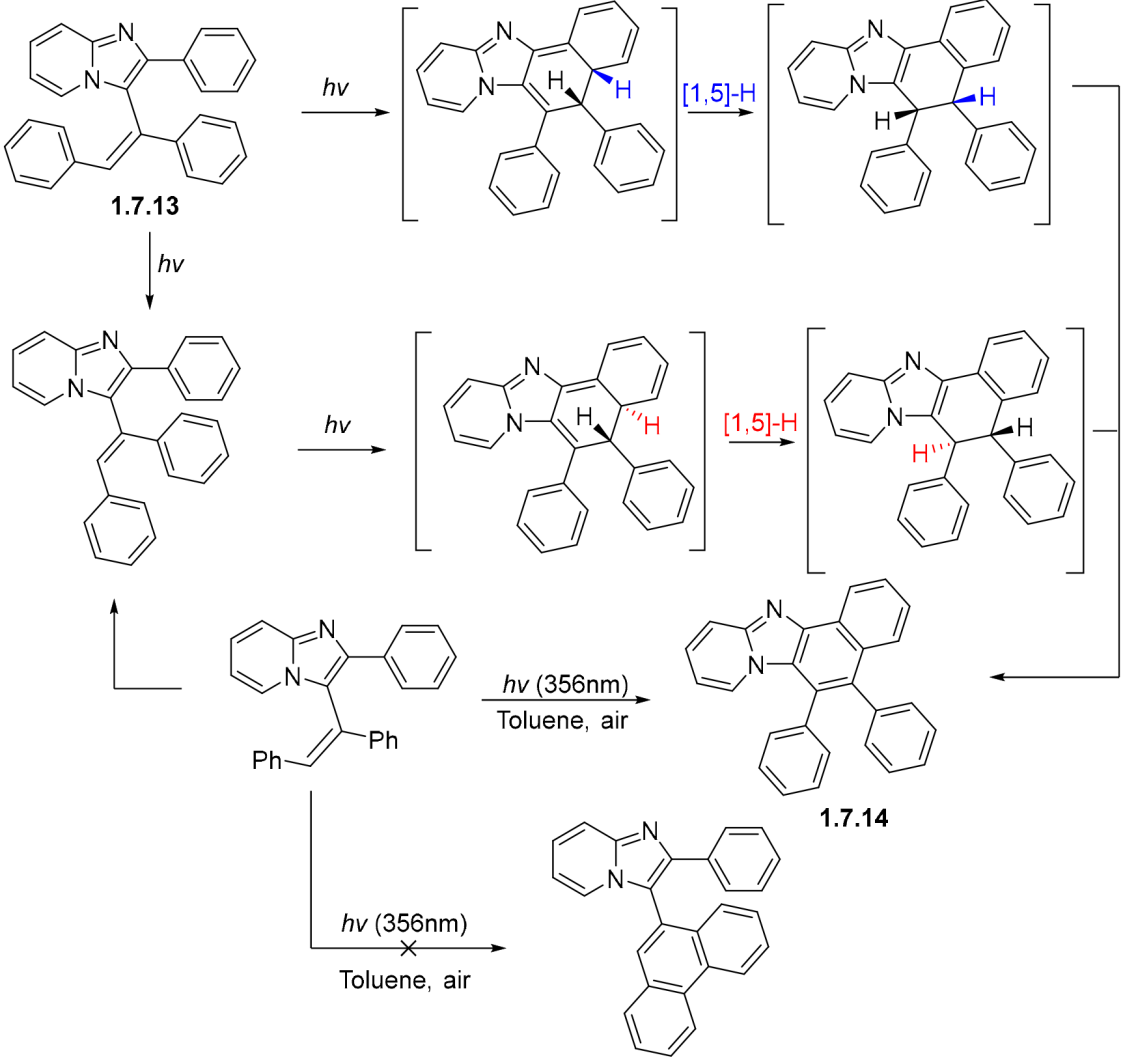
Competition between two modes of 6π-electrocyclization of 3-(1,2-diarylvinyl)-2-arylimidazo [1,2-a]pyridines.

Lvov et al. have explored the interesting cyclization of 3-(1,2-diarylvinyl)-2-arylimidazo [1,2-a]pyridines. In this example, the cyclization of **1.7.13** proceeds selectively between the imidazopyridine moiety and the alkene, instead of the expected cyclization in the stilbene unit. Cyclization proceeds via a 6π-electrocyclization, which disrupts aromaticity in the imidazopyridine ring. At this point, the molecule can either go back to the previous aromatic structure, with which it is in equilibrium, or proceed further via a [1,5]-hydrogen shift to regain aromaticity in both rings. This step happens suprafacially, thus maintaining the relative positions of the two hydrogens. The last step in this cyclization proceeds via oxidation from O_2_ to the fully aromatized product **1.7.14** (Scheme 42). Intermediates for the stillbene cyclization were never detected by the authors in the course of the reaction.

**Scheme 43.**
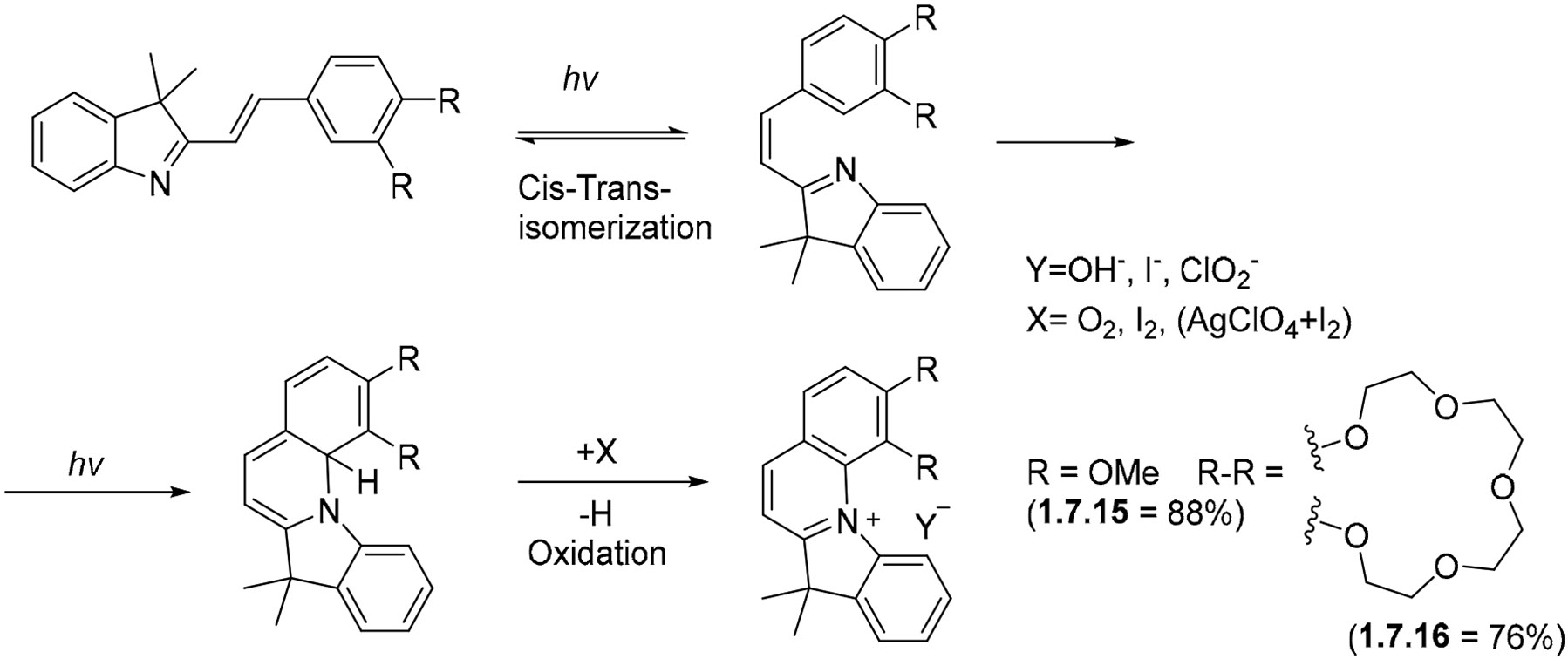
Irradiation of indolylphenylethenes (313 and 365 nm) in the presence of oxidants.

Novel variations of these processes continue to appear. For example, Fedorova et al. [132] found that, if the C-atom is blocked and the five-membered ring of an indole is taken out of conjugation, the photocyclization of indolylphenylethenes can proceed via the formation of a C–N bond. The initial cyclization at the indole nitrogen proceeds upon irradiation with the Hg Lamp (using 313 and 365 nm filters). The final heteroaromatic cations **1.7.15** and **1.7.16** (Scheme 43) are formed with the assistance of an oxidant and isolated as salts upon treatment with HClO4.

**Scheme 44.**
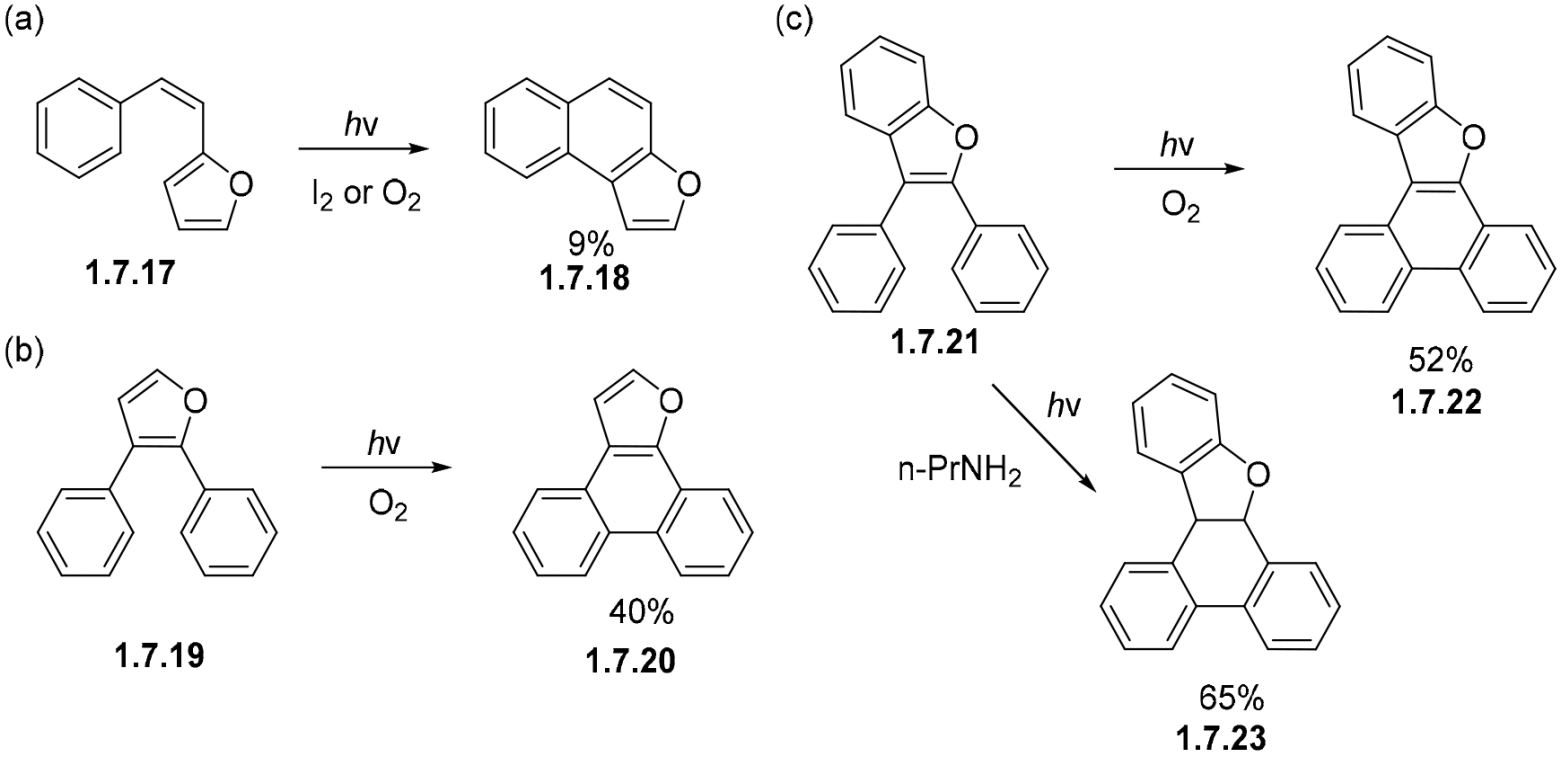
Photocyclizations of furan-containing analogs of stilbenes proceed to fully aromatized phenanthrene analogs in the presence of molecular oxygen or iodine: (**a**) furane at the periphery, (**b**,**c**) furane at the core.

The parent 2-styrylfuran **1.7.17** undergoes oxidative photocyclizations in very poor yields, e.g., only a 9% yield of **1.7.18** was formed with iodine or O_2_ as the oxidant (Scheme 44a) [133]. The relocation of the furan moiety to the center of this chromophore increases the efficiency of photocyclizations. For example, 2,3-diphenylfuran **1.7.19** forms phenanthrene **1.7.20** under oxidative conditions in a 40% yield (Scheme 44b) [134]. Benzofuran **1.7.21** gave the cyclic product **1.7.22** in an even higher (52%) yield under similar conditions (Scheme 44c) [135]. Interestingly, photocyclization in the presence of propylamine gave dihydroaromatic product **1.7.23**, presumably via a series of hydrogen transfers [135,136].

**Scheme 45.**
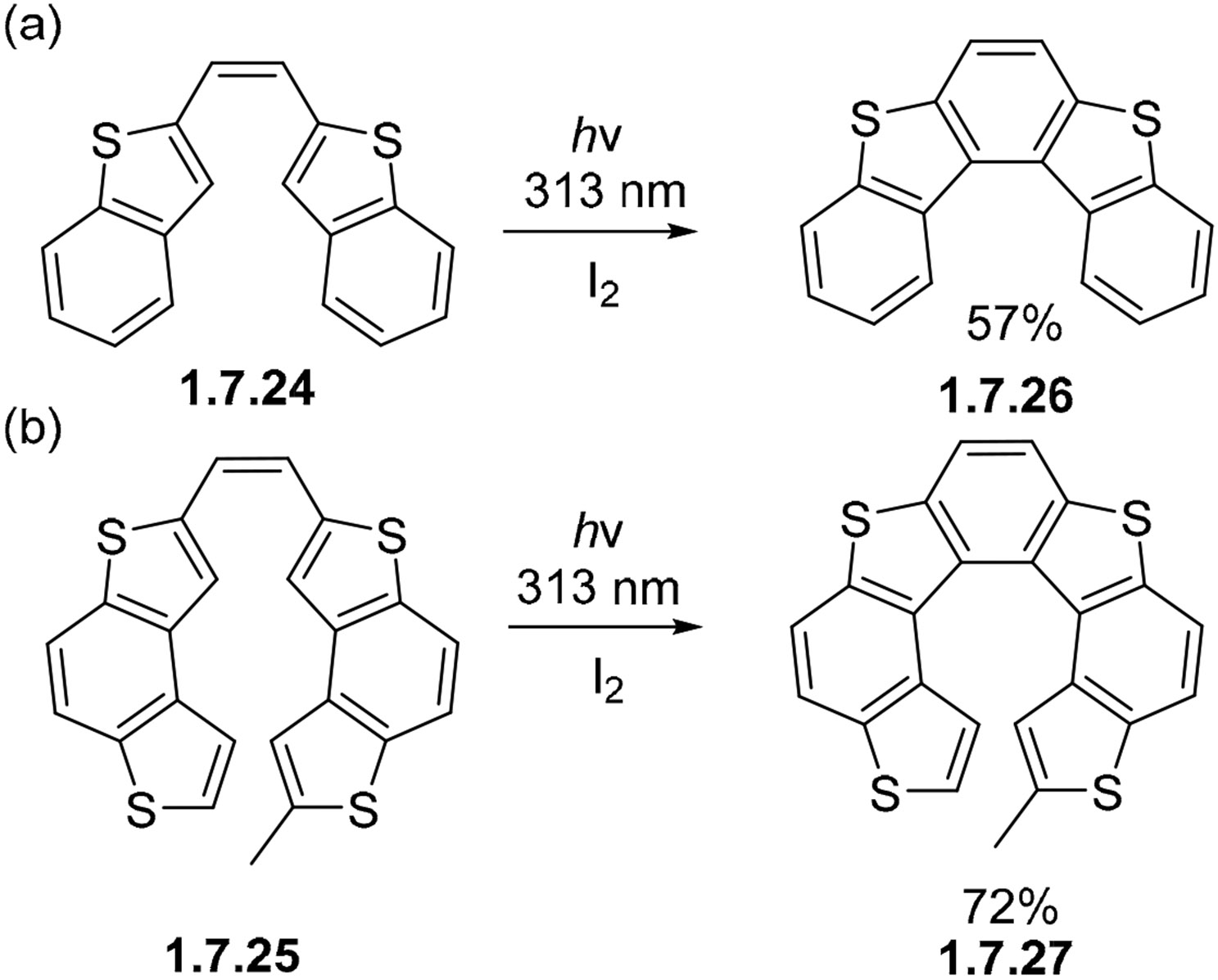
Thiophenes with expanded polyaromatic systems can participate in stilbene-like photocyclizations: (**a**) formation of [5]helicene, (**b**) formation of [7]helicene.

Thiophenes with expanded polyaromatic systems including **1.7.24** and **1.7.25** (Scheme 45a) readily undergo oxidative photocyclizations with iodine to afford **1.7.26** and **1.7.27** (Scheme 45b) in good yields [137,138]. The reaction proceeds in a similar fashion to stilbenes, and with the presence of an oxidant such as I_2_, fully aromatic products are formed.

Overall, the research into thiophene analogs of stilbenes is an active field of research due to their applications as photochromic molecules. The interrupted version of their Mallory cyclization (i.e., photocyclization without aromatization) has been widely used in the design of photochromic molecules and materials [139-141]. In this review, we will only show their simplest representatives, and how they relate to photocyclizations in general.

A particular common motif is the one wherein the phenyl rings of stilbene are substituted by thiophene rings. Because of the lower aromaticity of thiophenes, the driving force for cycloreversion is much lower. Consequently, the dihydro intermediate formed after the electrocyclization is often sufficiently stable to be isolated. The cycloreversion of these molecules can be promoted by light of different color, allowing full photochemical control over both the direct and reverse reactions, and many applications of such molecules in the design of sensors, components of molecular machines, etc.

**Scheme 46.**
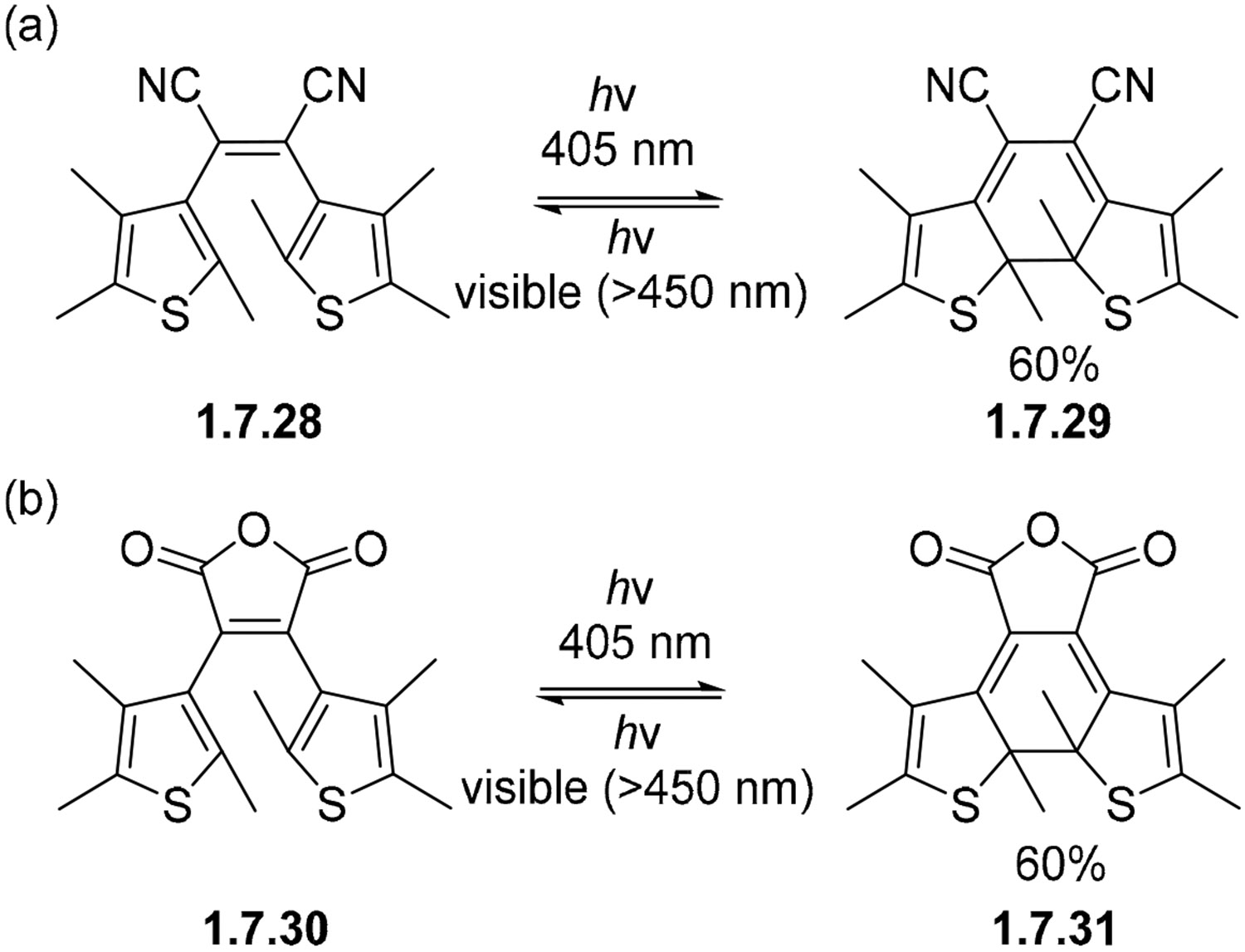
Selected reversible photocyclizations of photochromic bis-thiophenes (**a**,**b**).

**Scheme 47.**
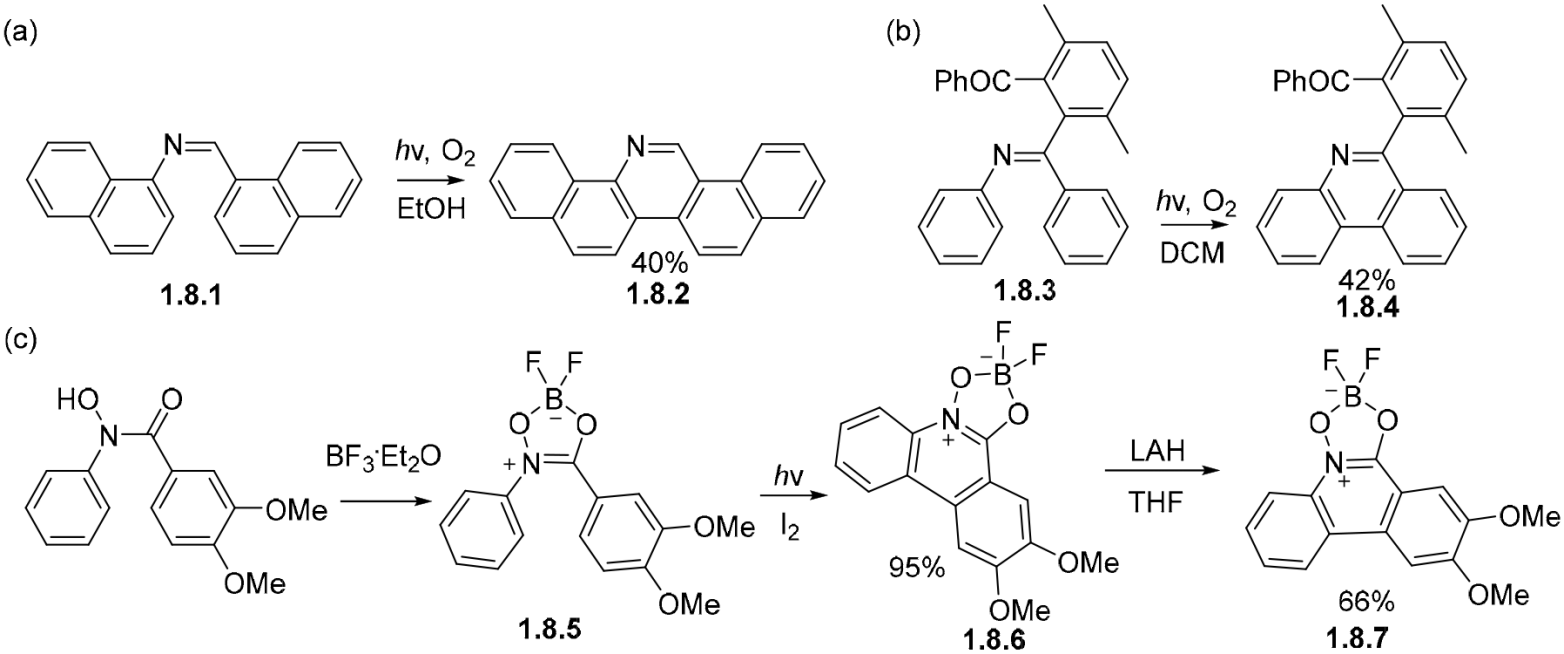
Stilbene-like photocyclizations of imines (**a**,**b**) and imine analog (**c**) lead to fully aromatized products.

Because this field is very large, we will limit ourselves to only the few representative examples shown in Schemes 46 and 47. The electrocyclizations of thiophene analogs **1.7.28** and **1.7.29** (Scheme 46a) into **1.7.30** and **1.7.31** (Scheme 46b) were prepared for use as thermally irreversible photochromic systems. These dihydro intermediates formed were stable for more than 3 months in the dark, yet readily reverted to the starting material when irradiated with visible light (>450 nm) [142].

#### 1.8. Photocyclizations Involving Non-Alkene Linkages

Although imines often need acidic conditions to undergo photocyclizations efficiently, examples can be found in which oxidations proceed without external acid being added. For example, imine **1.8.1** [143] undergoes cyclization in the presence of molecular oxygen in ethanol to produce **1.8.2** in a 40% yield (Scheme 47a). Likewise, imine **1.8.3** [144] undergoes oxidative photocyclization in dichloromethane, affording **1.8.4** (Scheme 47b). Alternatively, Lewis acids can be used to promote the reaction, as shown by the formation of 9-azaphenanthrenes such as **1.8.5** from hydroxamic acid **1.8.6** in the presence of BF_3_ ·Et_2_O [145] in a 95% yield, followed by reduction with lithium aluminum hydride to **1.8.7** (Scheme 47c).

**Scheme 48.**
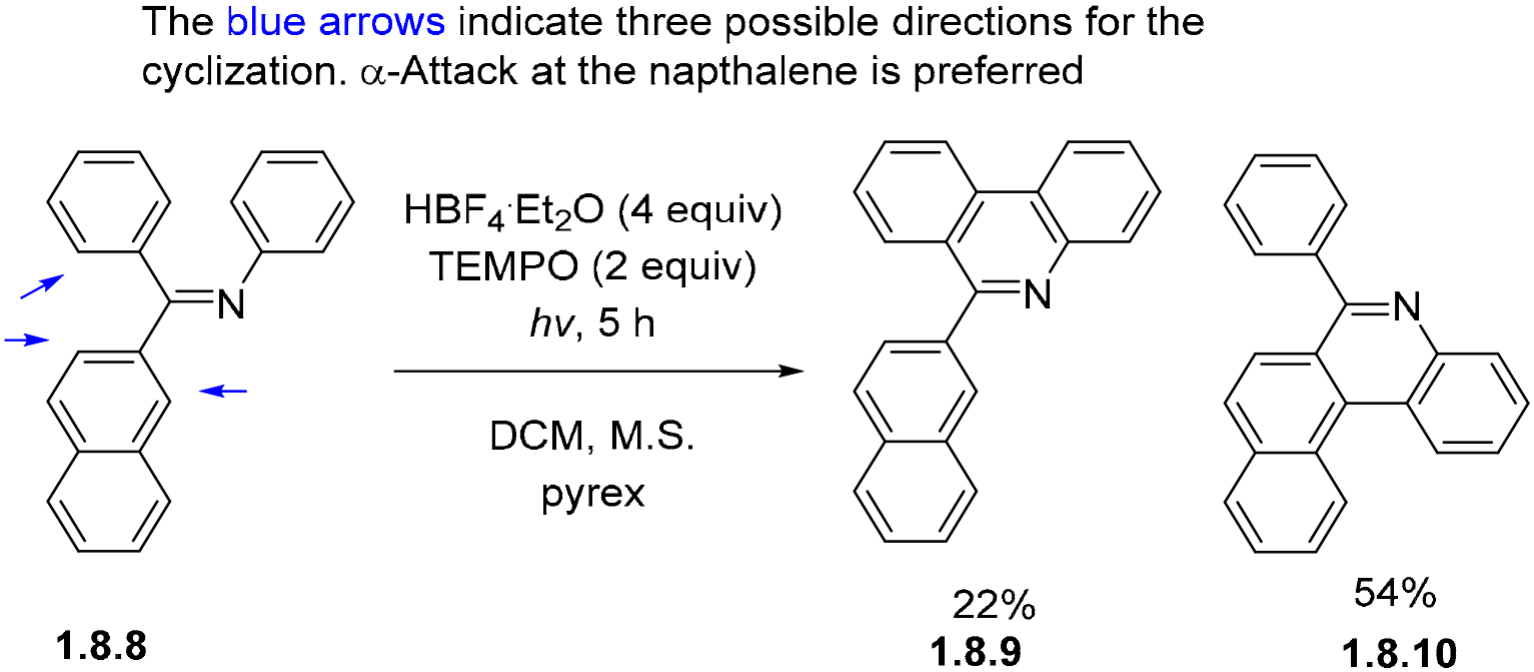
Additives can be used to promote full aromatization in photocyclizations of imine-bridged stilbene analogs.

Aza-PAHs can also be prepared from Schiff bases such as **1.8.8** in the presence of HBF_4_ ·Et_2_O. Interestingly, the reaction preferentially (and regioselectively) occurs at the naphthalene, ring as illustrated by the relative yields of benzophenanthridine **1.8.9** at 54% and phenanthridine **1.8.10** at 22% (Scheme 48) [146]. This example also illustrates the possible use of TEMPO for the aromatization of the initially formed photocyclization products.

**Scheme 49.**
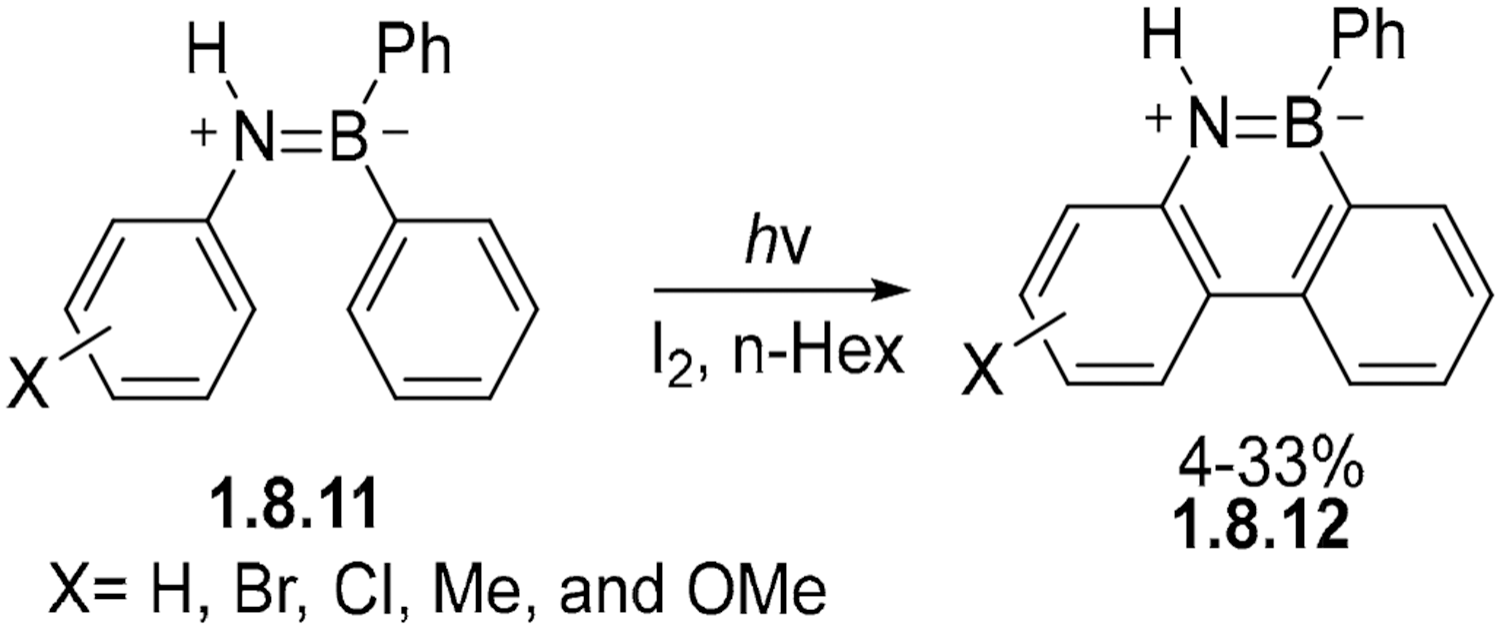
Scope of the stilbene-like photocyclization of B=N analogs.

Anilinoboranes undergo oxidative photocyclization, albeit with low yields. Specific examples come from the cyclization of **1.8.11** carried out under IR and full-spectrum irradiation over 15–24 h in the presence of I_2_ and hexanes to afford **1.8.12** at 4–33% for X=H, Br, Cl, Me, and OMe (Scheme 49) [147,148].

**Scheme 50.**
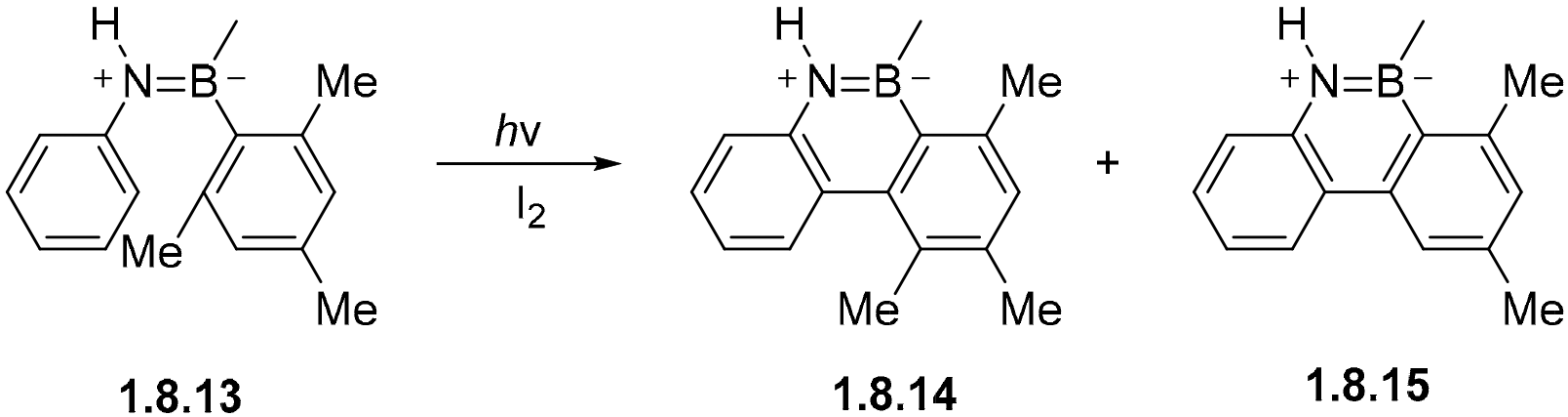
Methyl migration in the photocyclization of B=N analogs of stilbene.

An interesting example of migration was observed in the oxidative photocyclization of *o,o*-dimethyl anilinodiarylborane **1.8.13** carried out in the presence of 0.01 M I_2_ in cyclohexane (Scheme 50). Here, the formation of **1.8.14** by means of migration greatly dominates over the formation of **1.8.15** by means of Me-elimination. This is likely assisted by the higher concentration of I_2_ in solution, since the same reaction performed in a lower concentration of I_2_ (0.0005 M) gives solely the demethylated product **1.8.15** [147].

**Scheme 51.**
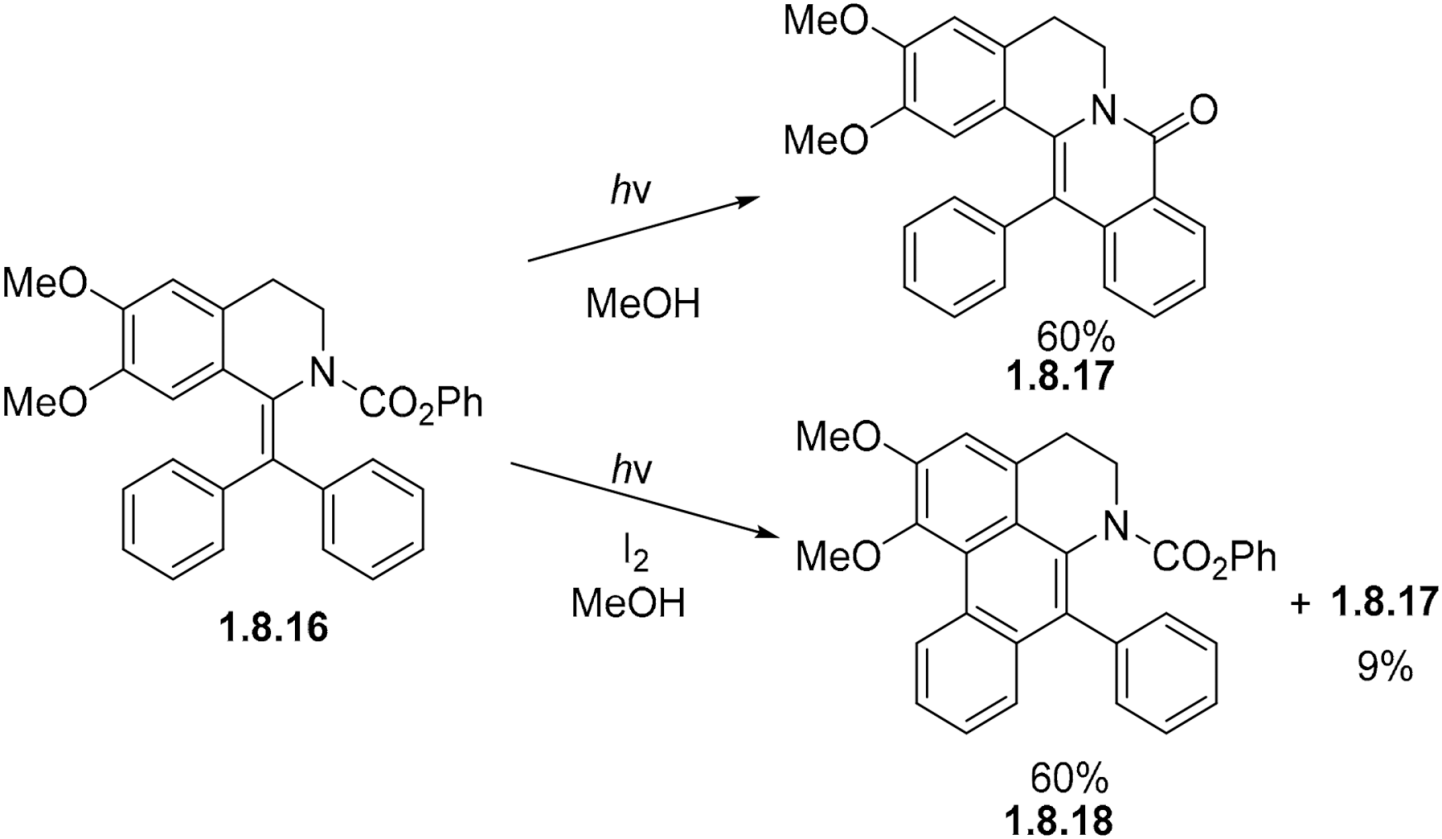
Stilbene carbamate selectivity controlled by photocyclization conditions.

The photocyclization of carbamate **1.8.16** under non-oxidizing conditions proceeds to lactam **1.8.17** in a 60% yield with phenanthrene **1.8.18** as the minor product (Scheme 51). However, under oxidizing conditions, phenanthrene **1.8.18** and lactam **1.8.17** are formed in 60% and 9% yields [149]. In both cases, the photocyclization of lactam was carried out in methanol. The difference in the outcomes again illustrates the reversibility of stilbene closures in the absence of an oxidizing reagent. In contrast, the initial photoproduct formed via carbamate cyclization does not need an oxidant to transform in the final stabilized product via the formal loss of PhOH.

**Scheme 52.**
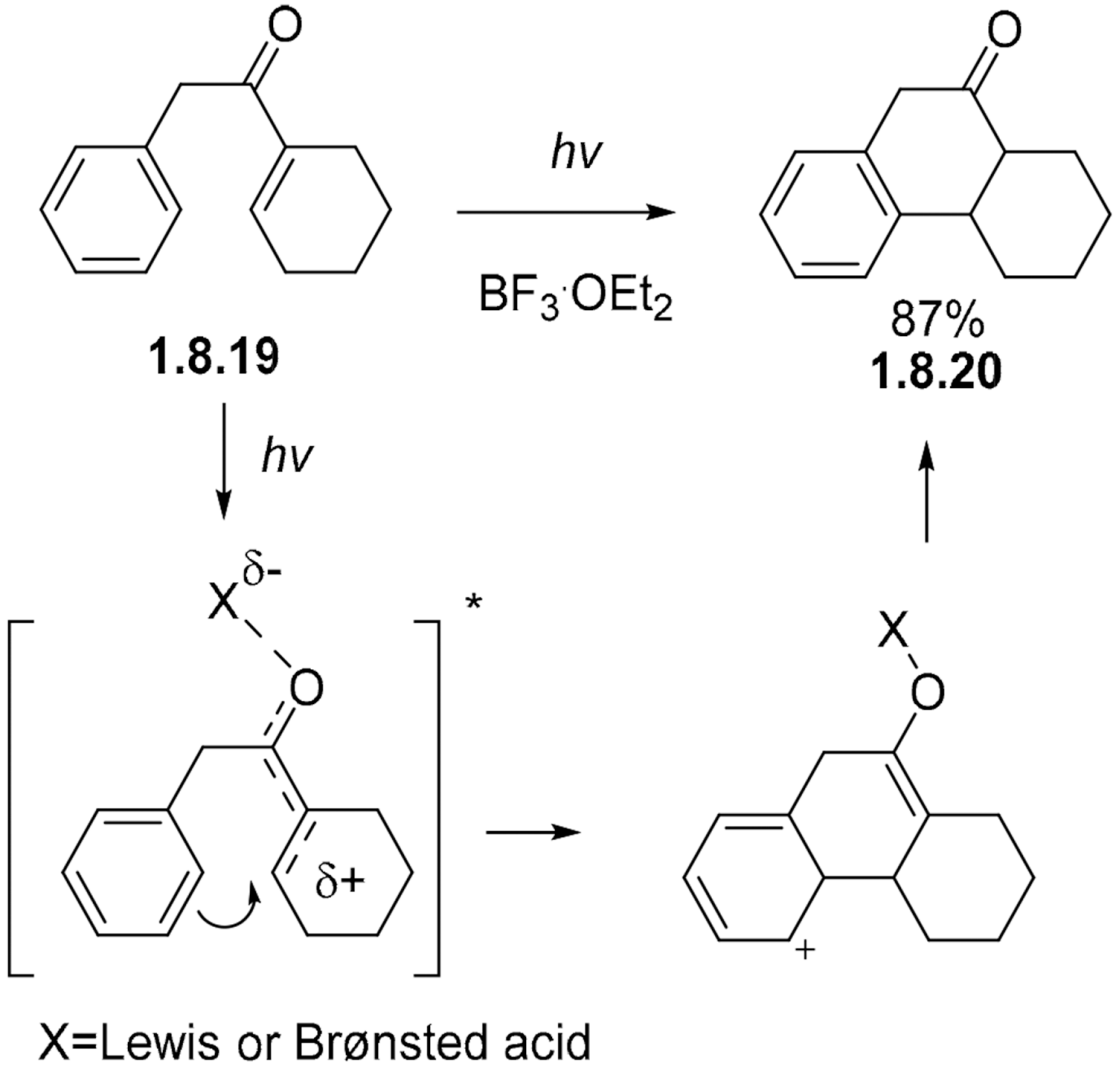
Stilbene–like photocyclization of a ketone in the presence of an acid.

The irradiation of ketone **1.8.19** proceeds to form a cyclic ketone **1.8.20** in an 87% yield. This process is suggested to proceed via an intramolecular Friedel–Crafts pathway with the assistance of light instead of an electrocyclic mechanism (Scheme 52) [150]. Interestingly, this process proceeds via both singlet and triplet states in benzene in the presence of BF_3_·Et_2_O. On other hand, efficient Stern–Volmer quenching with 1,3-pentadiene indicated that the non-catalyzed version of this reaction in methanol proceeds as a triplet process.

**Scheme 53.**
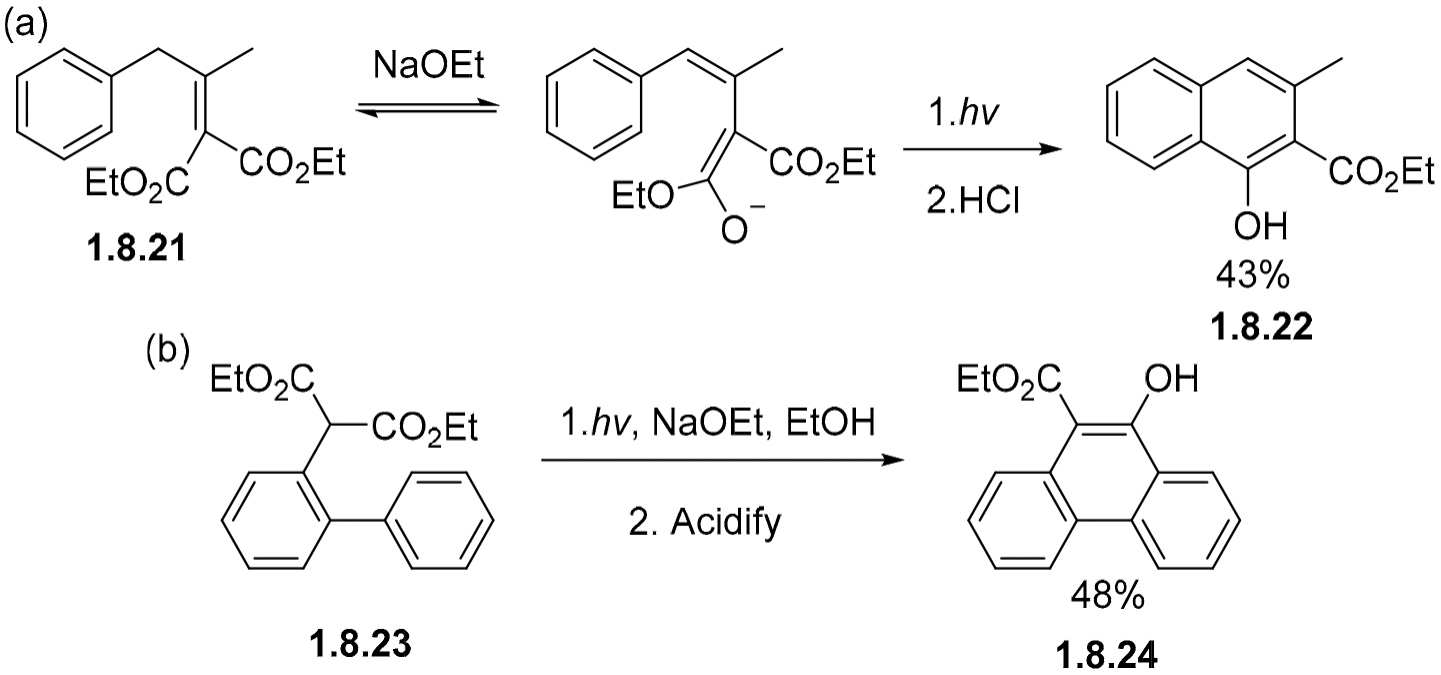
Photocyclization of enolates leads to fully aromatized products from substituted benzenes (**a**) and biphenyls (**b**).

The necessary alkene for photocyclization can come from an ester enolate. For example, the cyclization of ester **1.8.21** in ethanol in the presence of sodium ethoxide gives the naphthalene product **1.8.22** after acidic work up (Scheme 53a). The analogous photocyclization at the biphenyl moiety is reported for the enolate formed from ester **1.8.23**. This reaction also proceeds to form a fully aromatized product, phenanthrene **1.8.24**, in a 48% yield (Scheme 53b) [151]. Because aromatization proceeds through the loss of ethanol from the cyclized hemiacetal intermediate, these processes do not require an oxidizing agent.

**Scheme 54.**
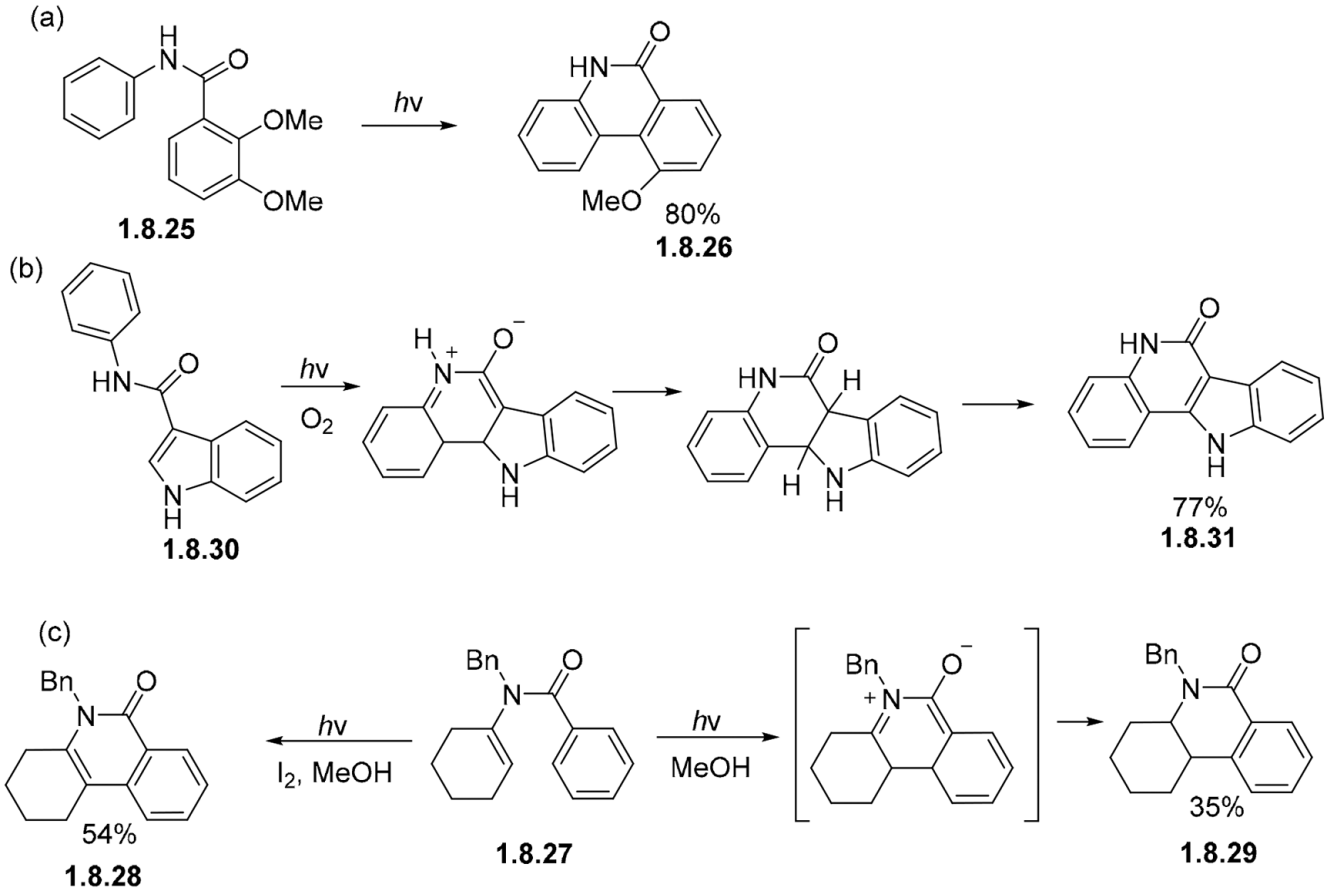
Photocyclizations of amides attached to aromatic systems: (**a**) benzenoid, (**b**) heterocyclic, (**c**) alkene partners.

Amides can also undergo different cyclizations depending on conditions and structure. For instance, the non-oxidative photocyclization of OMe-substituted benzanilide **1.8.25** proceeds at an 80% yield to give **1.8.26** via the elimination of methanol (Scheme 54a) [152]. The indole moiety can also serve as a partner in benzanilide cyclizations, as illustrated by the transformation of benzanilide **1.8.27** to the tetracyclic product **1.8.28** in good yields (Scheme 54c) [152,153]. Furthermore, the photocyclization of enamide **1.8.29** shows that only one aromatic ring is sufficient for the ring closure to occur. In the presence of iodine, the reaction gives either an unsaturated lactam **1.8.30,** while its saturated analog **1.8.31** is formed in the absence of oxidants (Scheme 54b) [154].

**Scheme 55.**
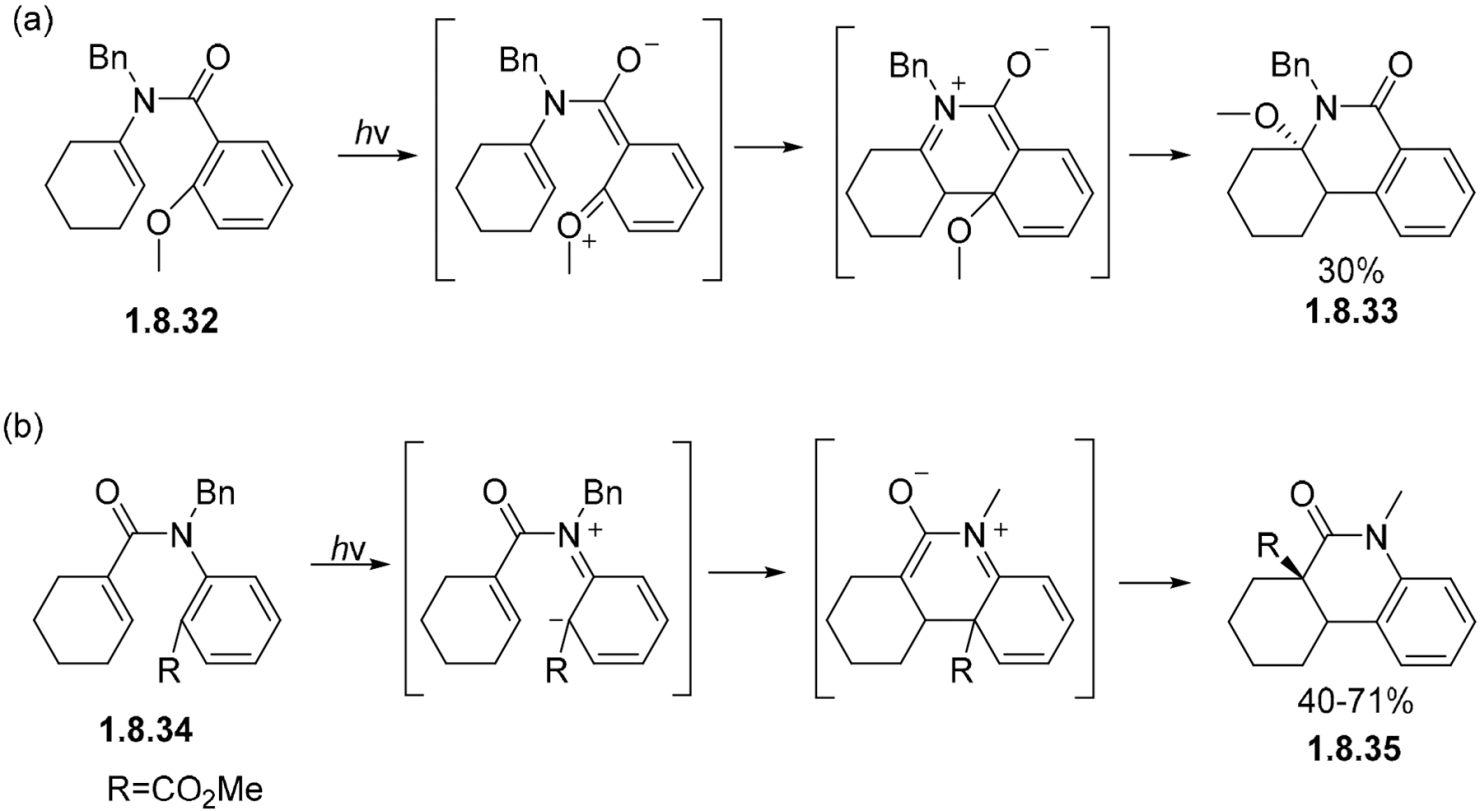
C-O (**a**) and C-C (**b**) bond migration in non-oxidative photocyclizations of amides.

Two interesting examples of stilbene-like cyclizations with amides come from the reaction of **1.8.32** [155,156] (Scheme 55a) and **1.8.33** [156,157] (Scheme 55b) to form aromatized lactams **1.8.34** and **1.8.35**. Interestingly, the cyclization proceeds selectively at the *ortho*-substituted carbon, and the substiuent is translocated during the aromatization step instead of the usual elimination (Scheme 55).

**Scheme 56.**
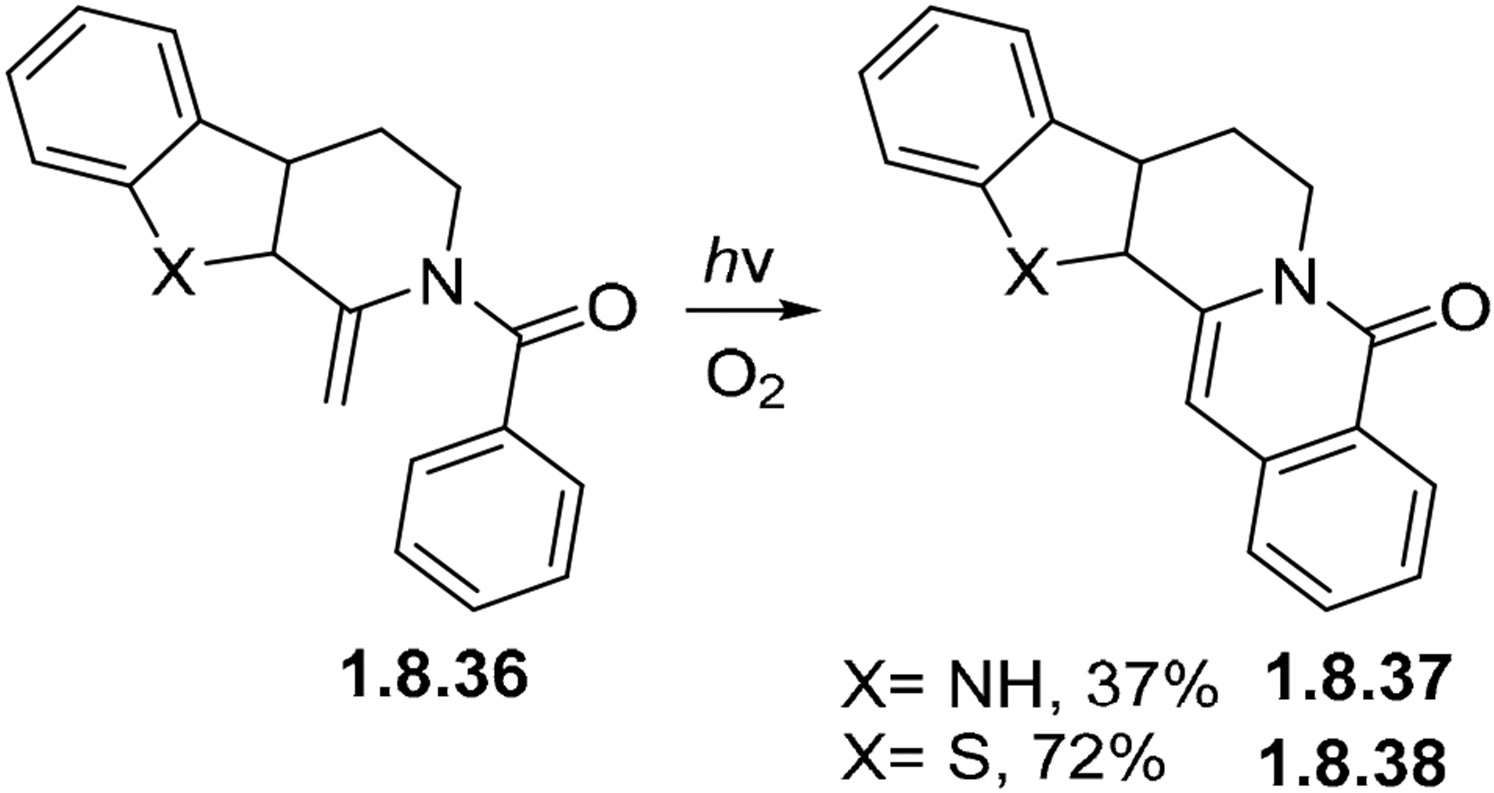
Enamide photocyclizations.

Polycycllc benzamides analogs **1.8.36** undergo oxidative ring closure with the formation of pentacyclic products **1.8.37** (X=NH) and **1.8.38** (X=S) in the presence of oxygen (Scheme 56) [156,158-160].

**Scheme 57.**
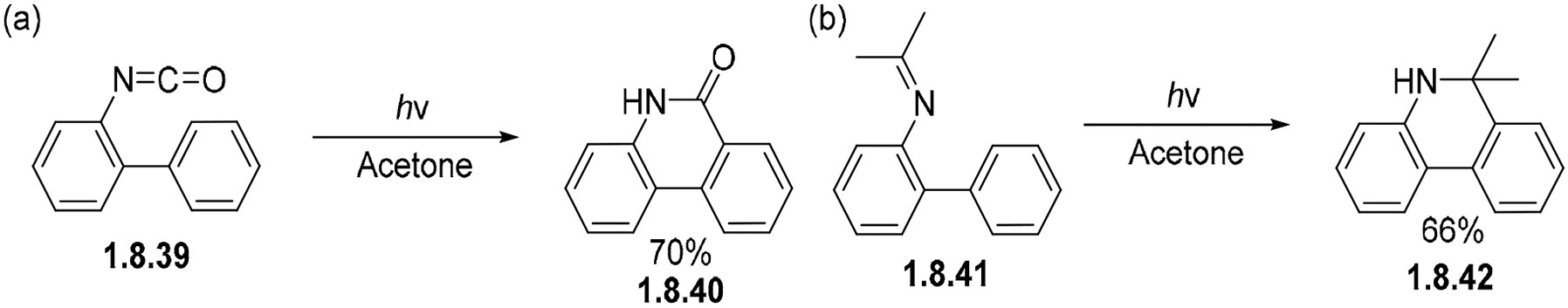
Photocyclizations of biaryls with N=C=O (**a**) and N=C (**b**) moieties.

Isocyanates often require triplet sensitizers to undergo cyclizations. Similar to the imines mentioned before, transformation was suggested to invovle a 1,5-hydrogen shift after the formation of a dihydroaromatic intermediate and, as such, isocyanate **1.8.39** [161] readily undergoes photocyclization in the presence of acetone to afford 6(5H)phenanthridone **1.8.40** in 70% yield (Scheme 57a). However, cyclizations of **1.8.41** [161] require the addition of triplet sensitizers such as acetone to afford the desired dihydrophenanthridine **1.8.42**, essentially forming an aromatized biphenyl unit (Scheme 57b).

#### 1.9. Photocyclizations Involving Single Heteroatom Linkages

**Scheme 58.**
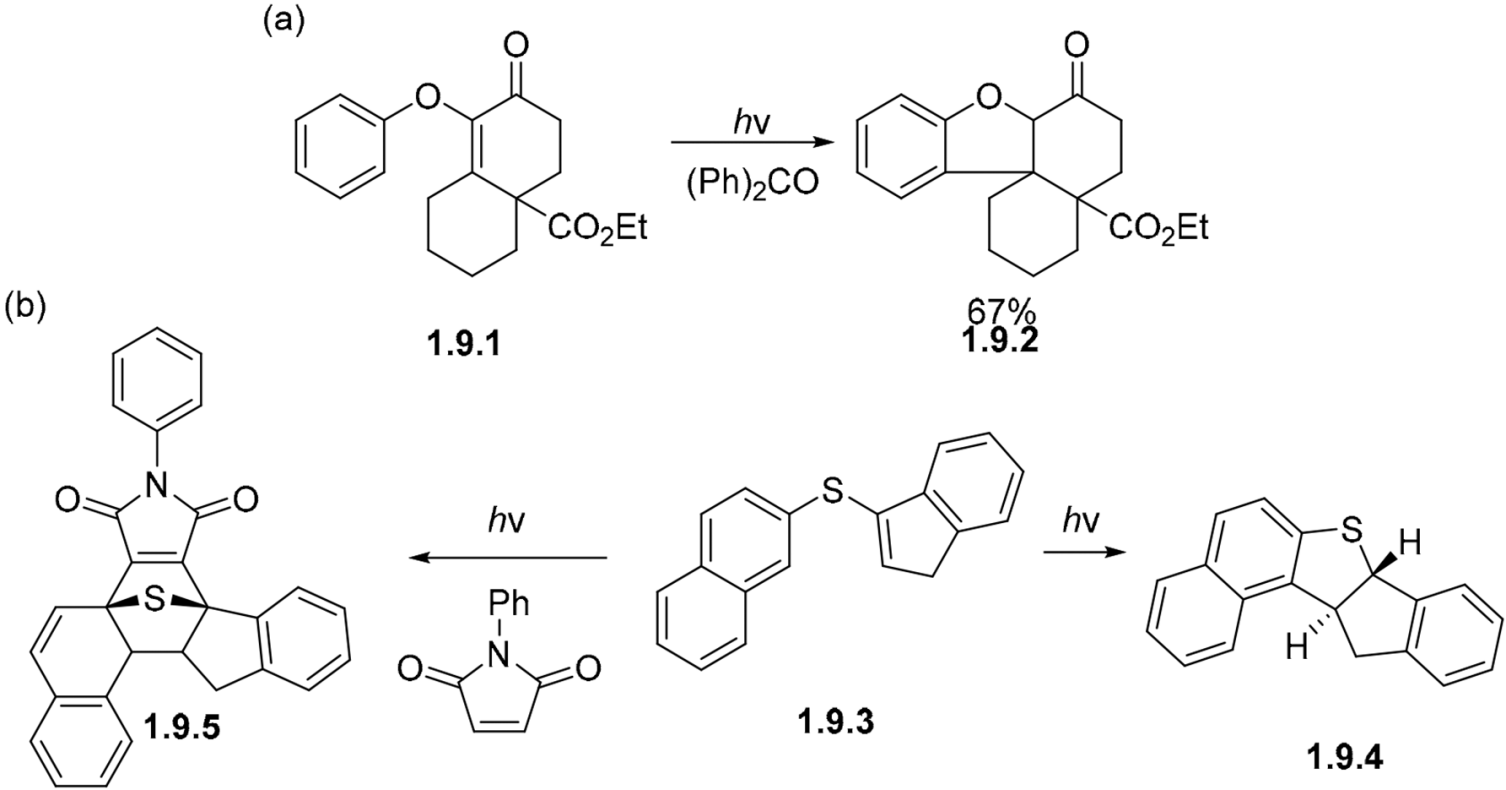
Cyclizations of aryls with a vinyl ether (**a**) and vinyl sulfide (**b**) groups.

Aryl vinyl ethers and aryl vinyl sulfides can undergo photocyclizations in the triplet state. The conversion of **1.9.1** to **1.9.2** in the presence of benzophenone as the triplet sensitizer gives a 67% yield (Scheme 58a) [162]. Sulfides such as **1.9.3** undergo cyclization in non-oxidative conditions yielding dihydro aromatic product **1.9.4** [163], or in the presence of N-phenylmaleimide to give adduct **1.9.5** (Scheme 58b) [162,163].

**Scheme 59.**
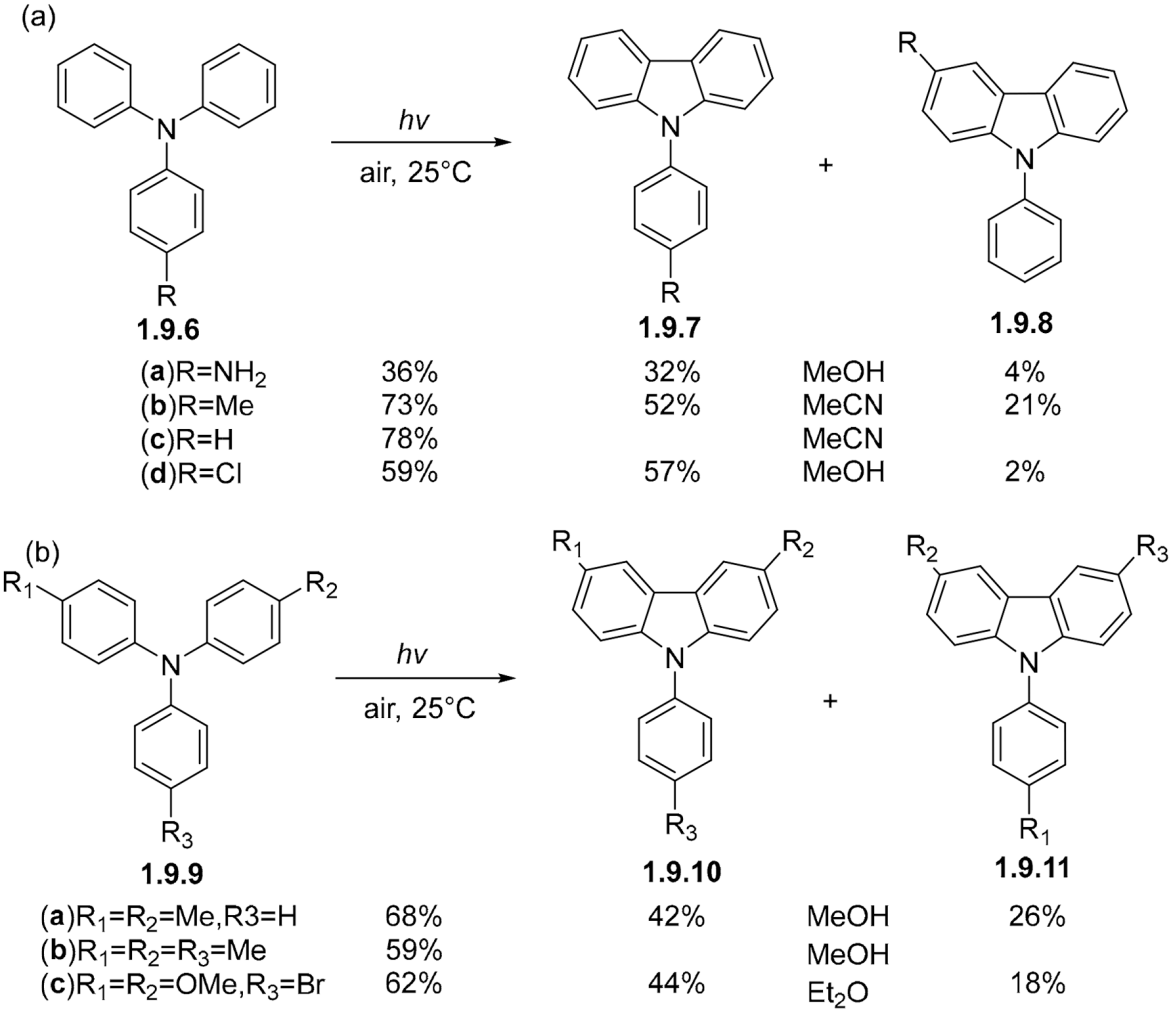
Photocyclization of triarylamines leads to carbazoles. (**a**) Triarylamines containing one substituent. (**b**) Triarylamines containing multiple substituents.

Carbazoles are widely utilized in optoelectronic materials, OLEDs, and conducting polymers, among other applications, due to their electronic properties and structural versatility. Scheme 59 illustrates the selectivity of cyclization in substituted triphenylamines. Mono-substituted triphenylamines **1.9.6a–d**, whether bearing electron-donating or electron-withdrawing groups, preferentially undergo cyclization to form carbazoles in which the R group is excluded from the carbazole core **1.9.7a–d** instead of **1.9.8a–d**. In contrast, di- and tri-substituted reactants **1.9.9a–c** predominantly cyclize to produce carbazoles incorporating donor groups within the carbazole framework, such as **1.9.10a–c**, instead of **1.9.11a–c** [164].

**Scheme 60.**
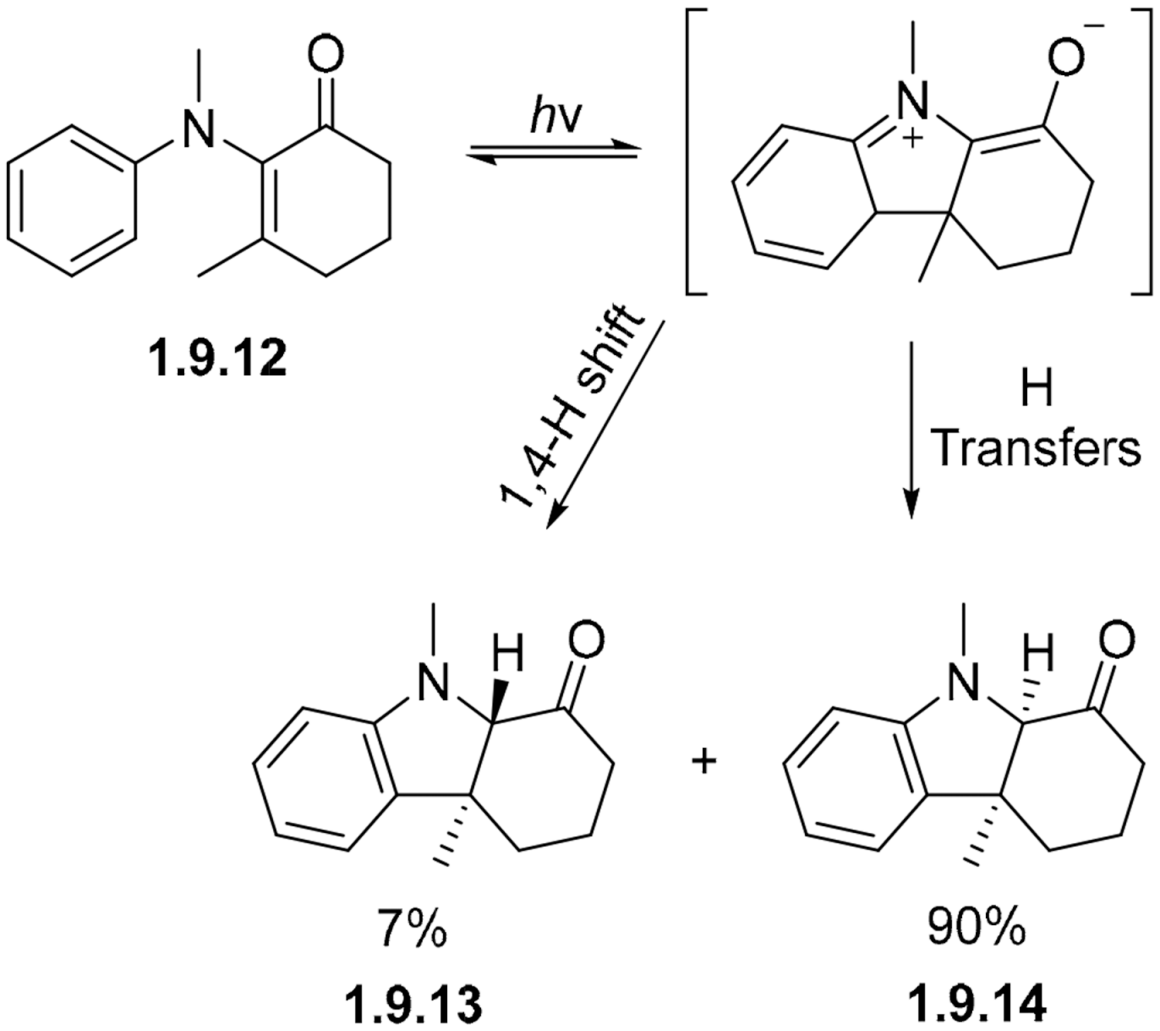
Divergent cyclizations of unsaturated keto analog.

The irradiation of enamine analog **1.9.12** proceeds to a mixture of stereoisomers **1.9.13** and **1.9.14** in 7% and 90% yields, respectively (Scheme 60) [165]. The *trans*-isomer could then be converted into the *cis*-isomer by treatment with methanolic sodium carbonate.

**Scheme 61.**
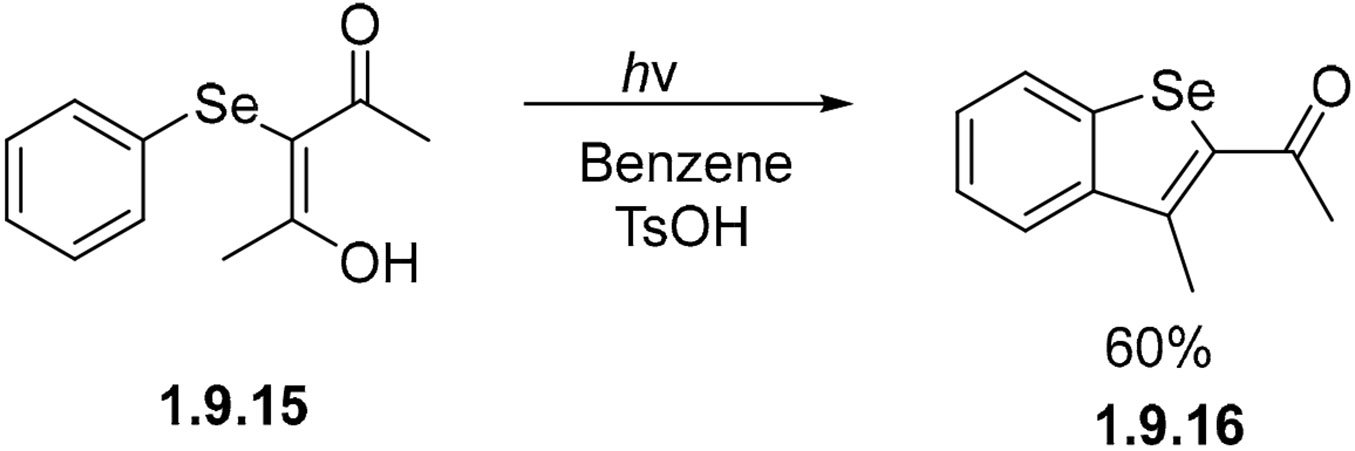
Photocyclization of selenide forming a five-member ring.

The acid-mediated photocyclization of selenide **1.9.15** dissolved in benzene gives benzoselenophene **1.9.16** in a 60% yield (Scheme 61) [166]. The OH group of the enol fragment can serve as a leaving group, making the cyclization irreversible. Acid helps to shift the equilbrium by promoting the dehydration of the dearomatized primary product of the photochemical cyclization. As a consequence, even the weak aromaticity of selenophene is sufficent for promoting the overall process.

**Scheme 62.**
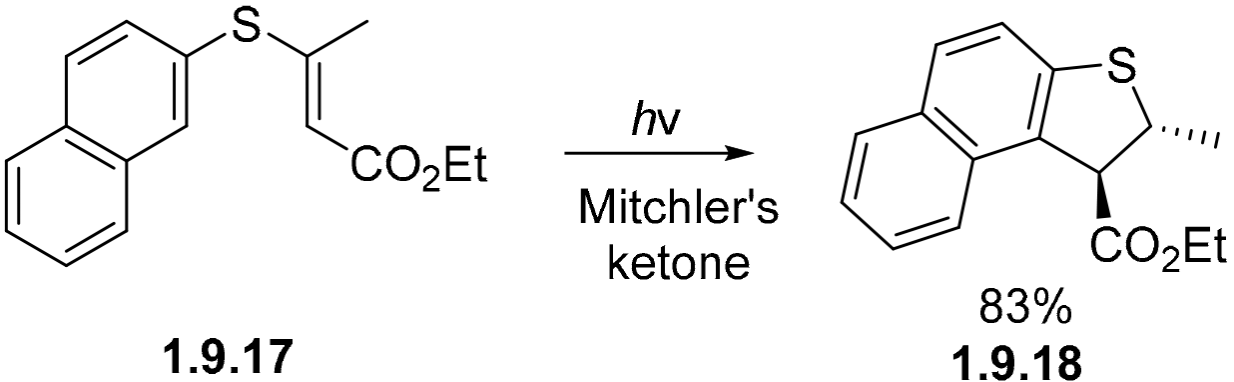
Cyclization of an aryl vinyl sulfide sensitized by mitchler’s ketone.

The photocyclization of aryl vinyl sulfides such as **1.9.17** occurs via triplet state sensitization (360 nm) with Mitchler’s ketone forming **1.9.18** in an 83% yield (Scheme 62) [167].

#### 1.10. Additional Photocyclizations Forming Five-Membered Rings

**Scheme 63.**
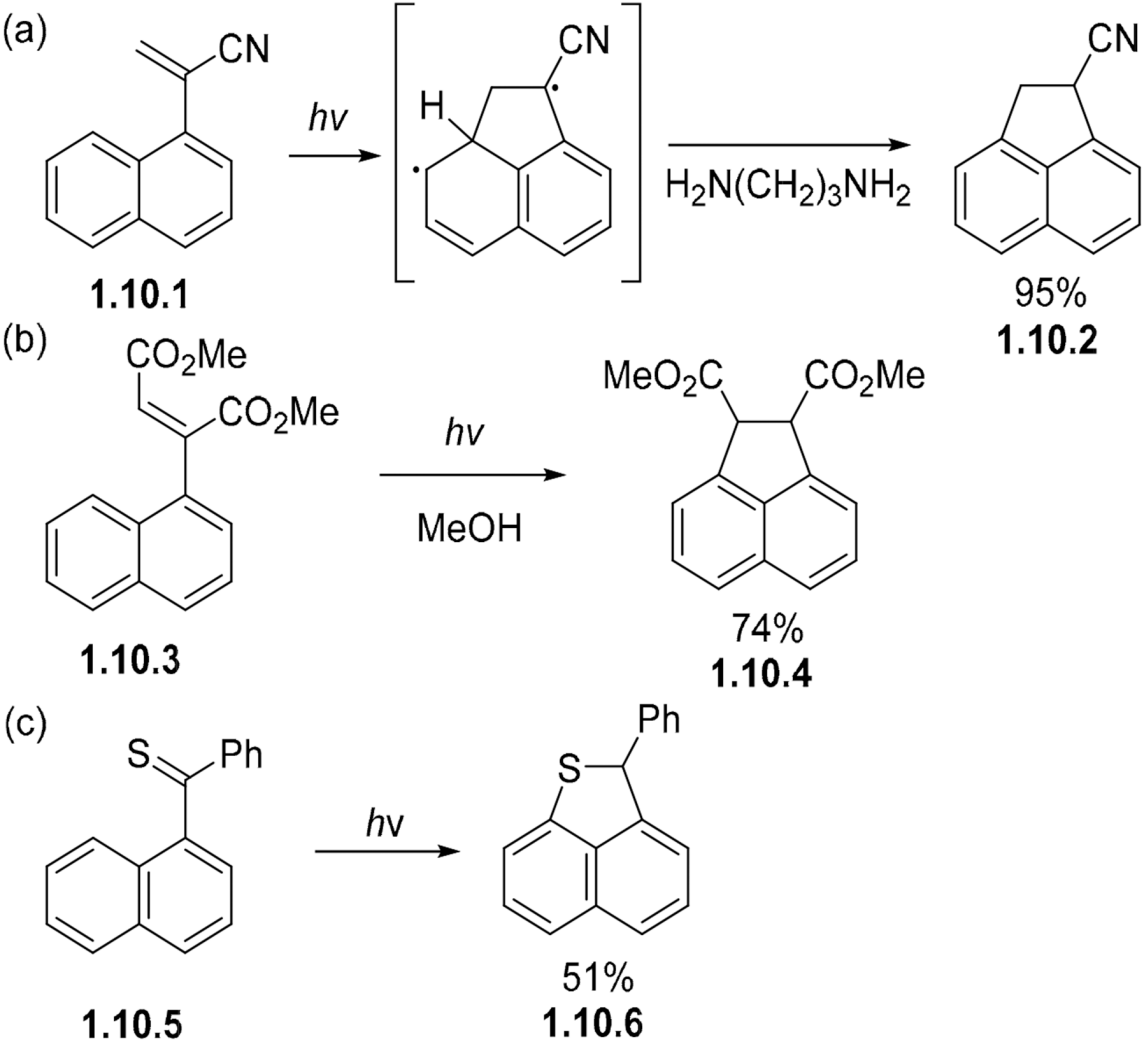
Photocyclizations of naphthalenes with vinyl (**a**,**b**) and thioketone (**c**) substituents.

Photocyclization reactions involving alkenes and arenes are also capable of forming five-membered rings even in the absence of heteroatoms. In the photocyclization of 1-vinylnaphthalene **1.10.1**, regioselective attack at the peri position gives a biradical intermediate (Scheme 63a). This biradical intermediate can then be trapped (via an ion pair) by a proton-transfer sequence with the amine to give cyano-acenaphthene **1.10.2** in a 95% yield [168]. The transformation of **1.10.3** to **1.10.4** occurs via a similar mechanism in a 74% yield (Scheme 63b) [169]. The irradiation of thioketone **1.10.5** proceeds cleanly to a cyclized product **1.10.6** (Scheme 63c) [170]. These cyclizations require a radical stabilizing group in the bridge.

## 2. Conclusions

**Scheme 64.**
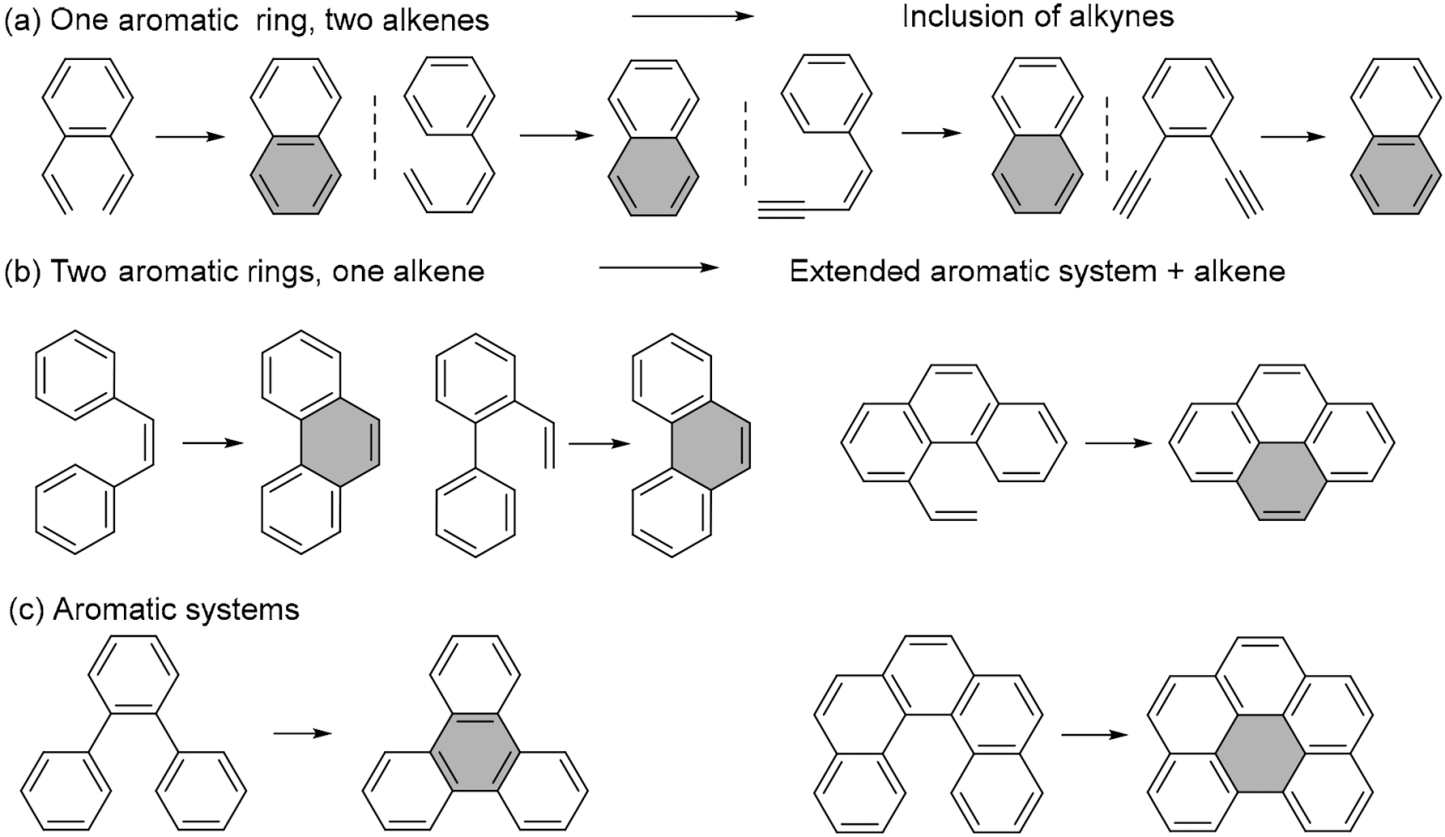
Summmary of photocyclizations with aromatic precursors.

Photocyclization reactions have been integral to synthetic chemistry for decades, offering a valuable route for constructing polyaromatic systems. Photochemical excitation activates both the alkene and arene moieties, converting them into highly reactive species capable of efficient C–C bond formation. As illustrated in Scheme 64, variations in the number of alkene and arene units allow for diverse cyclization patterns, with larger precursor molecules yielding more extended polycyclic systems.

Aromaticity plays an important role in these reactions. Although many of these reactions are formally electrocyclic and involve a thermodynamically favorable bond reorganization that converts one π-bond into a σ-bond, much of this thermodynamic advantage is used to pay for the loss of aromaticity (~20–30 kcal/mol). Here, the energy of visible or near-UV photons (~57 kcal/mol for the green light 500 nm photon or 82 kcal/mol for a near-UV 350 nm photon) plays an important role in assisting such chemical transformations, even when reactions proceed via non-concerted pathways. Interestingly, photochemical excitation can also enable ring opening, making many of these cyclizations reversible—an effect that is well known in the design of photochromic molecules.

This reversibility is much appreciated for stilbenes, in which the didehydrophenanthrene product often reverts to its acyclic precursor. To counter this, chemists employ strategies that promote rearomatization through subsequent exergonic reactions. The main approaches are based on either (1) eliminations/rearrangements or (2) oxidative rearomatization.

The first approach involves using *ortho*-substituted stilbenes where the substituent (X) is a good leaving group, such as Br or OMe. In these cases, photocyclization is followed by the elimination of an H-X molecule, yielding a fully aromatized product. In certain cases, varying the positions of the leaving groups can lead to alternative products. In the absence of properly placed leaving groups, 1,5- or 1,3-H shifts can induce partial aromatization.

The second approach involves the oxidative removal of C–H bonds. Due to the weakness of C–H bonds in the dearomatized cyclization products, even such mild oxidants like iodine or oxygen can remove the hydrogens and rearomatize the dihydrophenanthrene intermediates. This method is particularly popular in the synthesis of polycyclic aromatic compounds. Such transformations cross from a photochemical path to classic radical processes. Although the radical intermediates are often hidden and the radical part of the mechanism is treated as a black box, the cross-over between photochemistry and radical chemistry is mechanistically important and sometimes helpful in explaining the unexpected structural rearrangements and fragmentations.

Adjustments in reaction conditions, such as reaction time or the addition of oxidants, can lead to different products. For instance, the non-oxidative cyclization of 2,2′-distyrylbiphenyl under short irradiation times give the [2 + 2] cycloaddition product, while extended irradiation times yield tetrahydrophenanthrene. The same starting material, when irradiated in the presence of iodine, produces benzochrysene.

Heteroaromatic substrates also undergo efficient photocyclization. Protic solvents and/or acidic additives can enhance reaction rates, particularly for N-containing stilbene analogs. Substrates containing single heteroatom bridges (e.g., S, N, O, Se) can generate fused five-membered heterocycles.

In summary, photocyclization remains a powerful tool for accessing carbon-rich, π-conjugated structures that are challenging to synthesize via thermal activation. Its applicability to extended polyaromatics provides promising avenues for developing novel materials.

Despite extensive studies, many mechanistic aspects of these transformations remain unclear. While traditionally described as electrocyclic, a substantial fraction likely proceeds via non-concerted pathways involving high-energy diradical and zwitterionic intermediates. Under such conditions, even symmetry-forbidden reactions become feasible, highlighting the need for the further exploration of this dynamic field.

## Data Availability

No supporting data are available for this review. The information contained herein includes all necessary data used in the writing and analysis of these works.

## Abbreviations

The following abbreviations are used in this manuscript:

HMO: Hückel molecular orbital
LUMO: Lowest Unoccupied molecular orbital
HOMO: Highest occupied molecular orbital
PAH: Polyaromatic hydrocarbon
DHP: Dihydrophenanthrene
UV: Ultra-violet
OLED: Organic light emitting diode
